# Transcriptome Profiling of IL-17A Preactivated Mesenchymal Stem Cells: A Comparative Study to Unmodified and IFN-*γ* Modified Mesenchymal Stem Cells

**DOI:** 10.1155/2017/1025820

**Published:** 2017-02-15

**Authors:** Kisha Nandini Sivanathan, Darling Rojas-Canales, Shane T. Grey, Stan Gronthos, Patrick T. Coates

**Affiliations:** ^1^School of Medicine, Faculty of Health Sciences, University of Adelaide, Adelaide, SA, Australia; ^2^Centre for Clinical and Experimental Transplantation, Royal Adelaide Hospital, Adelaide, SA, Australia; ^3^Transplantation Immunology Group, Garvan Institute of Medical Research, Sydney, NSW, Australia; ^4^South Australian Health and Medical Research Institute, Adelaide, SA, Australia; ^5^Mesenchymal Stem Cell Laboratory, School of Medicine, Faculty of Health Sciences, University of Adelaide, Adelaide, SA, Australia; ^6^Central Northern Adelaide Renal Transplantation Service, Royal Adelaide Hospital, Adelaide, SA, Australia

## Abstract

Human mesenchymal stem cells pretreatment with IL-17A (MSC-17) potently enhances T cell immunosuppression but not their immunogenicity, in addition to avidly promoting the induction of suppressive regulatory T cells. The aim of this study was to identify potential mechanisms by which human MSC-17 mediate their superior immunomodulatory function. Untreated-MSC (UT-MSC), IFN-*γ* treated MSC (MSC-*γ*), and MSC-17 were assessed for their gene expression profile by microarray. Significantly regulated genes were identified for their biological functions (Database for Annotation, Visualisation and Integrated Discovery, DAVID). Microarray analyses identified 1278 differentially regulated genes between MSC-*γ* and UT-MSC and 67 genes between MSC-17 and UT-MSC. MSC-*γ* were enriched for genes involved in immune response, antigen processing and presentation, humoral response, and complement activation, consistent with increased MSC-*γ* immunogenicity. MSC-17 genes were associated with chemotaxis response, which may be involved in T cell recruitment for MSC-17 immunosuppression. MMP1, MMP13, and CXCL6 were highly and specifically expressed in MSC-17, which was further validated by real-time PCR. Thus, MMPs and chemokines may play a key role in mediating MSC-17 superior immunomodulatory function. MSC-17 represent a potential cellular therapy to suppress immunological T cell responses mediated by expression of an array of immunoregulatory molecules.

## 1. Introduction 

Human bone marrow derived mesenchymal stem cells (MSC) pretreated with interleukin-17A (IL-17A) represent a novel immunomodulatory strategy and an alternative to interferon-gamma (IFN-*γ*) treatment of MSC in enhancing MSC immunosuppression of T cells [1]. We have previously demonstrated that human MSC-17 potently suppresses human T cell proliferation and activation. In cocultures of MSC with purified human CD4^+^CD25^−^ T cells, MSC-17 induced high numbers of functionally suppressive iTregs [1]. Whilst MSC-17 appeared to be superior modulators of T cells, mechanisms exclusive to MSC-17 mediated immunomodulation warrant further investigation.

IL-17A is a member of the family of IL-17 cytokines secreted predominantly by the T helper 17 (Th17) subset of CD4^+^ T cells. IL-17A is a potent proinflammatory mediator and is involved in the pathogenesis of autoimmune diseases, allergic responses, and other immune cell mediated diseases including allograft rejection, sepsis, and graft versus host disease (GvHD) [2, 3]. Apart from the pathogenic roles of IL-17A, this cytokine is important for host defense response against fungal and bacterial infections [3, 4]. The IL-17A homodimer signals through the IL-17RA and IL-17RC dimeric receptor complex, where binding of IL-17A homodimer to the IL-17RA/RC complex recruits the key cytosolic adaptor molecule Act1 (NF-kappa B-activating protein), that is known to be the master mediator of downstream IL-17 signaling [3, 5]. Act1 binds to the IL-17RA/RC complex via its SEFIR (SEF/IL-17R) domains and this complex then recruits TRAF6 (TNF receptor-associated factor 6), leading to the activation of several downstream signaling pathways including the MAPKs-AP-1 (mitogen-activated protein kinases, MAPKs; activator protein-1, AP-1), C/EBPs (CCAAT/enhancer-binding proteins) and NF*κ*B (nuclear factor kappa B). Activation of these signaling cascades induces the gene expression of antimicrobial peptides, chemokines, MMPs, and proinflammatory cytokines as shown in other cell types such as endothelial cells, epithelial cells, and fibroblasts [3, 4]. IL-17A has emerged to be a growth factor for MSC by activating the Akt-Erk-MEK-p38 transduction molecules involved in MAPK signaling cascades [6–8]. Published work from our laboratory, demonstrated for the first time that IL-17A also enhances the immunomodulatory capacity of human MSC [1].

IFN-*γ* is produced predominantly by CD8^+^ T cells and NK cells and at lower levels by CD4^+^ T cells [9]. IFN-*γ* binds to a heterodimeric cell surface receptor complex consisting of the interferon-gamma receptor 1 (IFNGR1) and IFGR2, activating the classical JAK-STAT (signal transducer and activator of transcription) signaling pathways [10]. Activation of this pathway regulates several downstream cascades and induces expression of many genes, thereby contributing to the diverse biological effects of IFN-*γ* in different cell types [10–12]. IFN-*γ* activates macrophages to induce antitumor [13] and antimicrobial activities [14]. It is also well established that IFN-*γ* induces antigen processing and presentation pathways in different cell types for MHC antigen presentation to T cells [9, 15–17]. In B cells, IFN-*γ* regulates immunoglobulin production and class switching [16, 18]. IFN-*γ* also attracts leukocytes and favours the growth, differentiation, and maturation of many cells types [11, 16]. IFN-*γ* is classically known as a cytokine that favours Th1 cell development [16, 19]. In an allotransplantation setting, IFN-*γ* promotes antigen-specific Th1 differentiation that drives cell mediated allograft rejection [20]. Together, these findings suggest the potent proinflammatory role of IFN-*γ*.

The role of IFN-*γ* in MSC immunomodulation, reparative properties, and homing potential has been extensively reviewed as previously published [21]. IFN-*γ* treated MSC (MSC-*γ*) have enhanced immunomodulatory properties but are potentially immunogenic when administered in allogeneic or third-party hosts [1]. In this study, microarray and bioinformatics approaches were used to further identify novel candidate molecules expressed by MSC-*γ* and MSC-17 that enhance the immunomodulatory properties of MSC. Genes and biological processes that may contribute to MSC-*γ* immunogenicity in allogeneic or third-party hosts were also explored.

## 2. Materials and Methods

### 2.1. MSC Culture and Characterisation

Human bone marrow aspirates were obtained from the posterior iliac crest of normal adults volunteers (subjects with informed consent; age 20–35 yr) according to guidelines approved by the Human Ethics Committee of the Royal Adelaide Hospital, Australia (Protocol 940911a). Bone marrow derived MSC cultures were established and maintained as previously described [22, 23]. Cryopreserved MSC were cultured to log-phase and used at passage 6 in experiments. The immunophenotype of culture expanded MSC and their ability to differentiate into adipocytes, osteocytes, or chondrocytes have been confirmed and published [1].

### 2.2. Cytokine Treatment of MSC

MSC were seeded in tissue culture flasks at a density of 4000 cells/cm^2^ and were allowed to adhere overnight. Fresh MSC media containing either no cytokines or recombinant human cytokines, 500 U/ml IFN-*γ* (eBioscience) or 50 ng/ml IL-17A (Peprotech), were added to the MSC cultures to derive UT-MSC, MSC-*γ*, or MSC-17, respectively. At day 5, cytokines were washed out with Hank's Balanced Salt Solution (HBSS, Sigma) and modified MSC were used for microarray gene expression profiling and analysis.

### 2.3. Human MSC RNA Isolation

MSC were harvested using 0.25% trypsin/EDTA (Sigma) for 4 min, 37°C, and rinsed with 5% FBS/HBSS and RNA was extracted according to the protocol established by the Adelaide Microarray Centre (http://www.microarray.adelaide.edu.au/protocols/). Briefly, total RNA was extracted by dissolving the cell pellet in 500 *µ*L TRIzol reagent (Invitrogen) and 100 *µ*L chloroform was added to the mixture. The mixture was kept on ice for 15 min followed by centrifugation at 6500 ×g for 30 min, 4°C. The upper aqueous phase was retained and mixed with an equal volume of 70% ethanol in diethylpyrocarbonate H_2_O. Total RNA was further purified using the RNeasy mini kit (Qiagen) with the following modification: DNA was digested using the DNase I from the RNase-free DNase set (Qiagen). The quantity of total RNA was measured using NanoDrop 1000 (Thermo Scientific). Samples were adjusted to a concentration of 100 ng/*µ*L for microarray and were sent to the Adelaide Microarray Centre, University of Adelaide, for microarray gene expression profiling. The RNA sample was determined using the Agilent RNA Bioanalyzer. Only RNA samples with RNA integrity number (RIN) of ≥8 were used for microarray analysis.

### 2.4. Microarray Analysis

RNA extracted from human MSC samples were analysed using the Affymetrix Human Gene 2.0 ST Array (Affymetrix Inc., High Wycombe, UK) for gene expression profiling. Microarray gene expression profiling was performed on UT-MSC, MSC-*γ* and MSC-17 from 3 human MSC donor biological replicates (passage 6). Microarray experiments were conducted by the Adelaide Microarray Centre, University of Adelaide.

### 2.5. Microarray Quality Control and Gene Expression Analysis

Probe cell intensity (CEL) files were obtained from the Adelaide Microarray Centre. The Expression Console Software (Affymetrix) was used for data quality control, normalization, and differential gene level analysis. CEL files of each array showed no major issues or damage with the images. No outlier samples were identified based on the configurable QA/QC metrics. The RMA (robust multiarray analysis) algorithm was used to perform background subtraction, normalization, and summarization of probe sets. CHP files were generated from the Expression Console Software for further Principal Component Analysis (PCA) and gene level summarization using the Transcriptome Analysis Console (TAC) software (Affymetrix). After normalization, UT-MSC, MSC-*γ*, and MSC-17 from 3 donor samples of each treatment group were averaged and an unpaired one-way ANOVA was performed with significantly regulated genes identified by *p* < 0.05 and fold changes <−2 and >2. Gene lists for comparison of MSC-17 versus UT-MSC, MSC-*γ* versus UT-MSC, and MSC-17 versus MSC-*γ* were generated for subsequent bioinformatics analysis.

### 2.6. Functional Enrichment Analysis by DAVID

Gene lists for comparison of MSC-17 versus UT-MSC, MSC-*γ* versus UT-MSC, and MSC-17 versus MSC-*γ* were analysed for their biological functions using the Database for Annotation, Visualisation and Integrated Discovery (DAVID; https://david.ncifcrf.gov/). The gene list was uploaded using the official gene symbol onto DAVID for functional annotation clustering analysis with medium classification stringency, enrichment scores > 1.5, and *p* < 0.05 [24]. Functional annotation clustering analysis based on DAVID's default settings was performed. The gene sets were also subcategorised based on functional annotation of interest such as biological process (GOTERM_BP_FAT), molecular function (GOTERM_MF_FAT), and cellular component (GOTERM_CC_FAT).

### 2.7. Real-Time PCR Gene Validation

Genes of interest identified by microarray were validated by real-time PCR (RT-PCR) as previously described [1]. Gene specific human Taqman® primers MMP1 (Hs00899659_m1), MMP13 (Hs00233992_ml), CCL2 (Hs00234140_m1), CCL8 (Hs04187715_m1), CXCL6 (Hs00605742_m1), C3 (Hs00163811_ml), CH25H (Hs02379634_s1), and LBP (Hs01084621_ml) (Applied Biosystems) were used for gene expression analysis. Samples were run in triplicate and data were presented and normalized to the housekeeping gene hypoxanthine phosphoribosyltransferase-1 (HPRT1) (Hs99999909_ml). Mean normalized expression was calculated using the Qgene Module software as previously described [25].

## 3. Results

### 3.1. Transcriptome Profiling of UT-MSC, MSC-*γ*, and MSC-17

**Figure 1:**
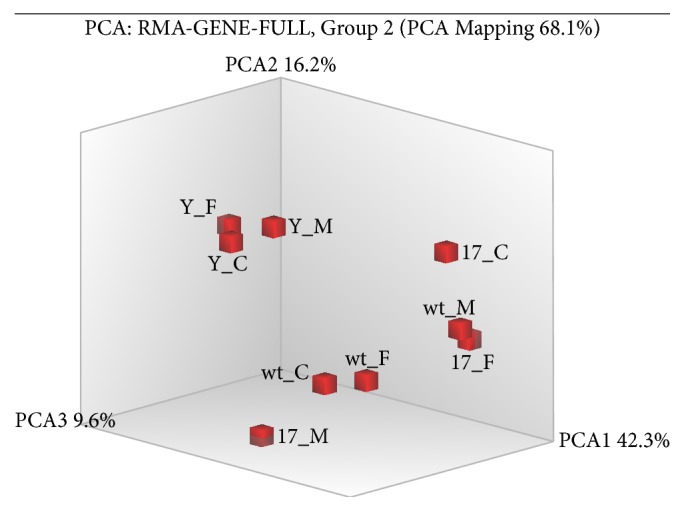
Principal Component Analysis (PCA) of UT-MSC, MSC-*γ*, and MSC-17. This 3-dimensional PCA graph identifies a new set of variables (PCA1, PCA2, and PCA3) that account for majority of the variability among the samples. PCA1 captures as much variability in the data as possible, PCA2 captures as much variability of the remaining variability not accounted by PCA1, and PCA3 captures as much of the remaining variability not accounted by PCA2. The symbols indicate IL-17A treated MSC, 17_; IFN-*γ* treated MSC (Y_); and untreated-MSC (wt_). The 3 different MSC donors are indicated by C, M, and F.

The transcriptome differences between UT-MSC, MSC-*γ*, and MSC-17 from 3 different human MSC donors were compared in this study. Principal Component Analysis (PCA) was performed to visualise variances between the 3 donors and treatment groups. PCA analysis revealed that the 3 donor replicates of MSC-*γ* “clustered” together. The gene expression pattern in the MSC-*γ* groups were clearly distinct from UT-MSC and MSC-17 (Figure 1). Microarray analysis revealed that 1278 genes (902 upregulated; 376 downregulated) were differentially regulated between MSC-*γ* and UT-MSC. The top 30 upregulated and downregulated genes in the MSC-*γ* were shown in Table 1.

There were however donor variances that exist between MSC-17 and UT-MSC. Among the 3 MSC donor samples evaluated, 2 MSC donors (i.e., donor C and F) “clustered” together and were distinct from UT-MSC (Figure 1). It should also be noted that in donor C and F MSC-17 “clusters,” there was less variability in the gene expression profile in MSC-17 versus UT-MSC compared to the MSC-*γ* versus UT-MSC groups. Donor M on the contrary had a different gene expression pattern in both UT-MSC and MSC-17. This clustering analysis in general supports a lesser degree of change in the gene expression profile of MSC with IL-17A than IFN-*γ*. Based on these 3 MSC donors, microarray analysis identified that only 67 genes (39 upregulated; 28 downregulated) were differentially regulated between MSC-17 and UT-MSC (Table 2).

**Figure 2:**
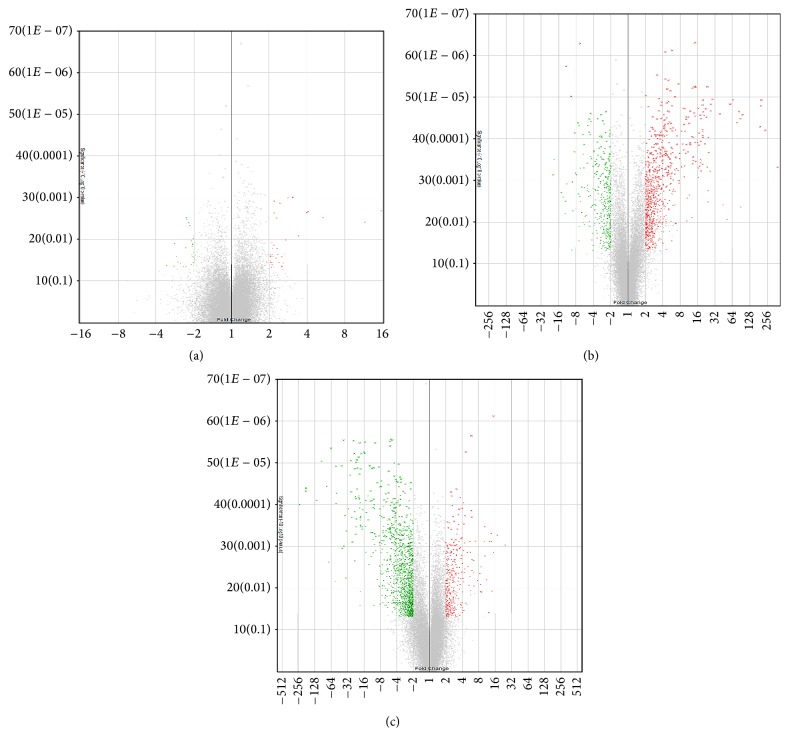
Volcano plots to identify changes in gene expression between (a) MSC-17 versus UT-MSC, (b) MSC-*γ* versus UT-MSC, and (c) MSC-17 versus MSC-*γ*. Axes of these plots represent significance (−10 log10  *p* value of the ANOVA *p* values; *y*-axes) versus fold changes (linear fold change from condition pairing; *x*-axes). Red colour indicates upregulated genes and the green represents downregulated genes. The grey region indicates genes that were not differentially expressed and not statistically significant.

**Figure 3:**
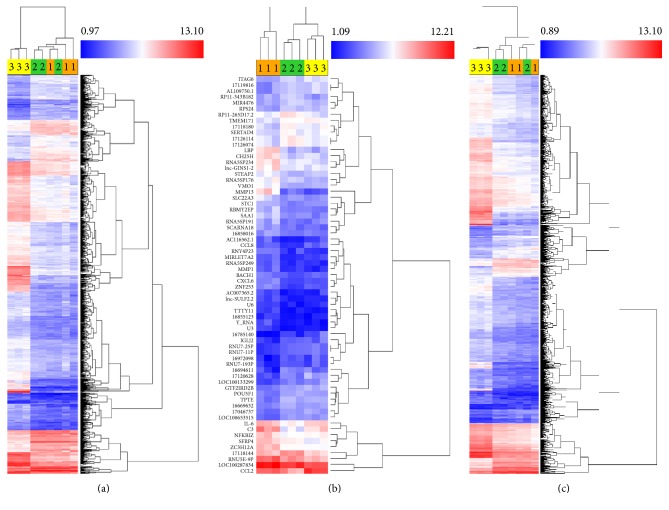
Gene expression profile of MSC-17 (1), UT-MSC (2), and MSC-*γ* (3) from 3 MSC donors determined with Affymetrix Human Gene ST 2.0 microarrays. Supervised hierarchical clustering of genes differentially expressed between (a) MSC-*γ* versus UT-MSC, (b) MSC-17 versus UT-MSC, and (c) MSC-17 versus MSC-*γ* determined by ANOVA *p* value (condition pair) *p* < 0.05 and fold change (linear) <−2 or >2. (a) 1278 and (b) 67 genes were differentially regulated between the treatment groups. The normalized expression value for each gene is visualised by a colour gradient: blue represents low gene expression; red represents high gene expression.

The gene expression profile of MSC-17 versus MSC-*γ* was also evaluated. The clustering of the 3 MSC donors in the MSC-17 and MSC-*γ* comparison groups was more distinct (Figure 1) when compared to MSC-17 and UT-MSC. Microarray analysis revealed that 1806 genes (391 upregulated; 1415 downregulated) were differentially regulated between MSC-17 and MSC-*γ*. The top 30 upregulated and downregulated genes in the MSC-17 versus MSC-*γ* comparison group were shown in Table 3. Volcano plots (Figure 2) and supervised hierarchical clustering of the differentially expressed genes (Figure 3) provided a global visualisation of genes regulated by IL-17 or IFN-*γ* treatment of MSC compared to UT-MSC.

### 3.2. MSC-*γ* Enriched for Genes Associated with Increased Immunogenicity

Upregulated and downregulated gene lists were submitted to DAVID for functional annotation clustering analysis to identify gene sets that were enriched in MSC-*γ*. There were 90 and 62 official gene symbols from the upregulated (see Table S1 in Supplementary Material available online at https://doi.org/10.1155/2017/1025820) and downregulated (Table S2) gene entry lists, respectively, that were unmapped by DAVID. These were mainly noncoding genes including microRNA (miRNA), long noncoding RNA (lncRNA), and small nucleolar RNA (snoRNA). Gene ontology analysis by DAVID functional annotation clustering was performed on the upregulated and downregulated MSC-*γ* versus UT-MSC gene lists to identify enriched gene sets for biological processes (Tables S3, S4), molecular functions (Tables S5, S6), and cellular components (Tables S7, S8).

Gene ontology analysis for biological processes of upregulated MSC-*γ* genes (Table S3) uncovered highest enrichment of genes associated with antigen processing and presentation via MHC class I (annotation cluster 1, enrichment score 8.03). These genes were mainly HLA type genes and have roles in antigen presentation. Enriched genes in annotation cluster 1 also include aminopeptidases that hydrolyse antigenic peptides for MHC class I peptide binding and antigen presentation (e.g., endoplasmic reticulum aminopeptidase ERAP1 and ERAP2), peptide transporter genes (e.g., transported associated with antigen processing, TAP2), and other genes involved in the antigen processing and presentation pathway (e.g., TAP binding protein, TAPBPL; *β*_2_ microglobulin, B2M; CD74). Gene sets involved with antigen processing and presentation via MHC class II were also upregulated in the MSC-*γ* groups (annotation cluster 4, enrichment score 4.45). In annotation cluster 2 (enrichment score 6.06) there were also enriched gene sets involved in immune response activation (innate, adaptive, and lymphocytes mediated immunity), humoral response (immunoglobulin mediated immune response, B cell mediated immunity, and humoral immune response mediated by circulating immunoglobulin), and complement pathways (classical and alternative) activation.

Apart from genes that are involved in increased MSC-*γ* immunogenicity, there were genes with regulatory roles upregulated in MSC-*γ* (Table S3). For example, these gene sets were involved in the regulation of programmed cell death, apoptosis, translation regulation, protein modification, transcription regulation and DNA binding activity, cell-cell communication, and signal transduction as well as the regulation of cytokine production. Moreover, genes upregulated in the MSC-*γ* group were enriched for the TGF-*β* receptor signaling pathway (annotation cluster 19, enrichment score 1.74, e.g., FMOD, CCL2, MAPK3K1, SMAD6, GDF15, and TGFB2). Other genes of interest upregulated in MSC-*γ* include IL-6, toll-like receptor-3 (TLR3), TLR4, and indoleamine 2,3-dioxygenase (IDO), with the gene ontology term for positive regulation of defense response. There was also upregulation of the PD-L1 transcript in MSC-*γ* compared to UT-MSC (3.46-fold, *p* < 0.0104; data not shown), consistent with the observed increase in cell surface protein expression of PD-L1 following IFN-*γ* pretreatment of MSC, as we have previously published [1]. Regulatory genes with nucleotide binding activity and transcription (corepressor, repressor, and cofactor) activity were also enriched and upregulated in MSC-*γ* as identified by DAVID gene ontology analysis for molecular function (Table S5).

MSC-*γ* have enhanced migratory potential to sites of inflammation [21]. Based on DAVID analysis for biological processes, we have identified gene sets in annotation cluster 10 (enrichment score 2.78) that were enriched for the gene ontology terms regulation of cell motion, cell migration, and locomotion (Table S3). These upregulated MSC-*γ* genes include chemokines (CXCL10, CXCL16), intracellular adhesion molecule-1 (ICAM1), IL-6, and VEGFA. The upregulation of chemotactic factors that may increase MSC-*γ* homing potential was also identified when gene ontology analysis for molecular function was performed on DAVID. In annotation cluster 3 (enrichment score 3.10; Table S5), genes were enriched for chemokine receptor binding and chemokine activity. These chemokines include CCL13, CCL2, CXCL16, CXCL9, CCL8, CXCL11, and CXCL10.

Based on the downregulated MSC-*γ* versus UT-MSC gene list, we identified that there were genes highly enriched for the gene ontology terms for biological processes involving extracellular matrix or structure organisation (annotation cluster 1, enrichment score 11.10; Table S4), consistent with our previous observation of changes in MSC-*γ* morphology from fibroblastic-like appearance to a hypertrophic flattened irregular shape [1]. These were mainly collagen type genes (collagenases I, III, IV, V, XI, XII, and XIV). Interestingly, the downregulated gene sets also have enriched terms for biological processes involved in the cell division cycle (annotation cluster 2, enrichment score 8.80; Table S4) These downregulated genes were essential for M phase, nuclear division, mitosis, and cell division. Genes essential for regulation of cell-cycle division were also downregulated in MSC-*γ* (annotation cluster 7, enrichment score 2.03), in coherence with the observation of decreased MSC-*γ* growth kinetics compared to UT-MSC [1].

Gene ontology analysis for cellular components (Tables S7, S8) of the differentially regulated genes in the MSC-*γ* versus UT-MSC groups uncovered that these genes were located in the extracellular space (or) region (annotation cluster 1, enrichment score 2.69, Table S7; enrichment score 12.76, Table S8). Many downregulated genes were located in collagen, the main structural protein in the extracellular region.

### 3.3. MSC-17 Enriched for Genes Associated with Chemotaxis

Differentially regulated genes were submitted to DAVID for functional annotation clustering to identify gene sets that were enriched in MSC-17. Genes that were mapped by DAVID were shown in Table 2. There were 23 genes from the gene entry list that were unmapped by DAVID (Table S9). These include noncoding genes, lncRNA, ribosomal RNA (rRNA), snoRNA, and miRNA.

Functional annotation clustering analysis was first performed using DAVID's default settings (Table S10) to identify overall gene sets that were highly enriched in MSC-17 compared to UT-MSC. Annotation cluster 1 with the highest enrichment score (3.58) had enriched terms for genes residing in the extracellular region, roles in inflammatory response, response to wounding, defense responses, signaling, and disulfide bonds (Table S10). Gene ontology analysis of biological processes also revealed that MSC-17 compared to MSC-*γ* were upregulated and enriched for genes involved in angiogenesis (e.g., angiogenin, CXCL12, tissue plasminogen activator, and collagens), wound healing, and chemotaxis responses (Table S14). Interestingly, some gene sets were enriched for gene ontology terms such as glycosylation and glycoproteins (Table S10), which may relate to posttranslational modification processes [26, 27]. There was also high enrichment of genes involved in chromatin remodelling processes (enrichment score 7.35, Table S14), suggesting the potential gene expression regulatory roles of MSC-17.

Human MSC-17 were shown to be superior at regulating T cell inflammatory responses by suppressing T cell proliferation, activation, and secretion of proinflammatory cytokines [1]. In annotation cluster 3 (enrichment score 2.48, Table S10), genes such as IL-6, C3, serum amyloid A1 (SAA1), and lipopolysaccharide binding protein (LBP) were enriched for regulation of immune responses. IL-6, SAA1, and LBP also have roles in regulation of cytokine production.

Gene ontology analysis by DAVID functional annotation clustering was also performed on the MSC-17 versus UT-MSC and MSC-17 versus MSC-*γ* gene lists to specifically determine enriched gene sets for biological processes (Tables S11, S14, and S15), molecular functions (Tables S12, S16), and cellular components (Tables S13, S17, and S18) in MSC-17. There was no significant enrichment of gene sets for molecular functions in the downregulated gene list of MSC-17 versus MSC-*γ* comparison group.

MSC-17 were previously shown to mediate Treg induction via cell-cell contact dependent mechanisms [1]. To identify potential cell surface candidate molecules that mediate MSC-17 induction of Tregs, the cellular compartments of genes enriched in the MSC-17 were also evaluated. Functional enrichment for biological processes identified a set of upregulated genes (IL-6, CCL8, SLC22A3, STC1, and CXCL6) that were enriched for the gene ontology term cell-cell signaling (fold enrichment: 4.9; *p* < 0.0148; annotation cluster 2, Table S11). Chemokines CCL2, CCL8, and CXCL6 detected by DAVID's functional enrichment for molecular function (Table S12) showed evidence that these gene sets have a different range of binding potential including chemokine receptor, heparin, glycosaminoglycan, pattern, and polysaccharide binding activities. These MSC-17 enriched genes, mainly the chemokines and MMPs, were located in the extracellular space (or) region (Table S13).

Biological processes (GOTERM_BP_FAT; Tables S11, S14) and molecular functions (GOTERM_MF_FAT; Table S12) of MSC-17 enriched genes were mainly associated with cell migration and chemotaxis responses. MMPs were also highly enriched in the MSC-17 groups. Specifically, MMP13 (FC 15.6) and MMP1 (FC 2.4) were induced in the MSC-17 groups as detected by microarray gene expression analysis (Tables 2 and 3). DAVID's bioinformatics analysis revealed that these genes were enriched for gene ontology terms such as secreted, extracellular space, signal, disulfide bond, glycosylation, glycoproteins, and response to stimulus (Table S10). The MSC-17 versus UT-MSC gene list when analysed by DAVID functional annotation chart (default setting) showed that these MMPs where highly enriched for metal ion binding, peptidase, and collagen degradation functions (Table S10).

### 3.4. MSC-17 Express Chemokines and Matrix Metalloproteinases

**Figure 4:**
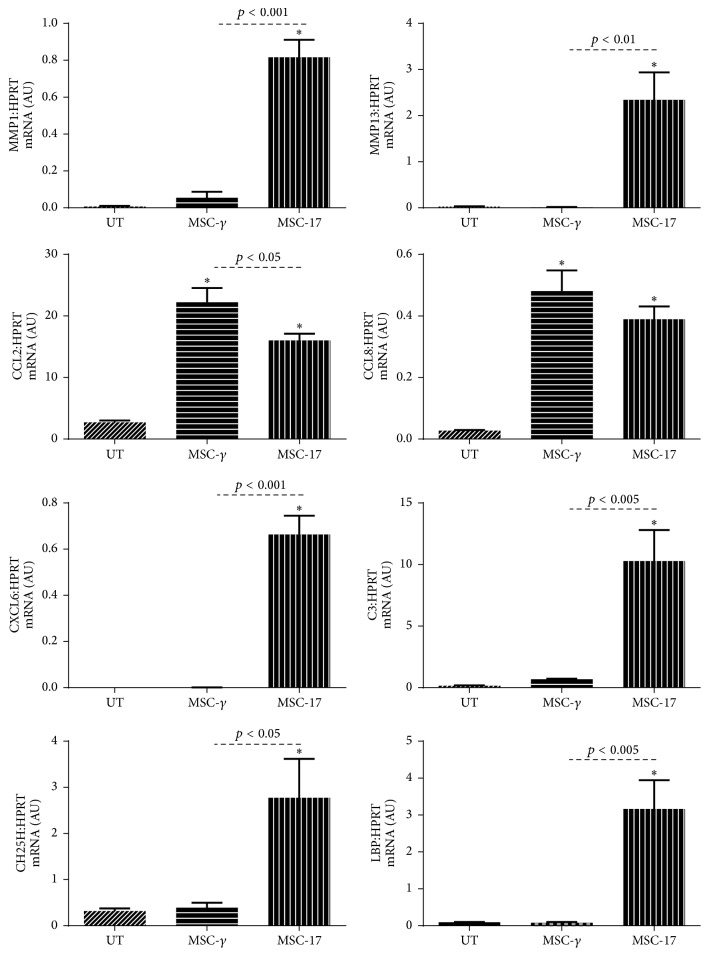
Microarray gene expression validation by RT-PCR. Gene expression of MMP1, MMP13, CCL2, CCL8, CXCL6, C3, LBP, and CH25 in MSC detected by microarray was validated by RT-PCR following 5 days of IL-17A or IFN-*γ* treatment of human MSC. ^*∗*^*p* < 0.05 versus UT-MSC was determined by one-way ANOVA with post-Sidak multiple comparison test. Data are representative of 3 human MSC donors. Error bars depict mean ± SD.

To validate the microarray data of MSC-17, upregulated genes were evaluated for their gene expression by RT-PCR (Figure 4). IL-17A induced the expression of MMP1, MMP13, CXCL6, C3, CH25H, and LBP in MSC as determined by microarray and validated by RT-PCR (*p* < 0.05). CCL2 and CCL8 were highly expressed in both MSC-*γ* and MSC-17 compared to UT-MSC, consistent with the microarray data. CCL2 gene expression increased by 8.2- and 5.9-fold in MSC-*γ* and MSC-17, respectively, relative to UT-MSC. CCL8 expression on the other hand was comparable between MSC-*γ* and MSC-17. Although the gene expression levels varied between the 3 MSC donors, these genes were consistently upregulated relative to UT-MSC in all the MSC donors.

## 4. Discussion

IFN-*γ* preactivation of human MSC induced the expression of various immunoregulatory molecules including IDO, TLR3/4, IL-6, and PD-L1 that may enhance the inhibitory activity of MSC-*γ* to mediate T cell suppression. IDO is a well characterised immunosuppressive molecule expressed by MSC upon induction with IFN-*γ* [1, 28–30]. Administration of IDO deficient MSC (IDO^−/−^MSC) or inhibition of IDO activity resulted in accelerated kidney allograft rejection, decreased intragraft, or circulating Tregs and showed absence of donor-specific tolerance [29]. IDO^−/−^MSC were also incapable of inhibiting donor DC maturation and function, thus enabling DC to stimulate strong recipient T cell proliferative responses [29]. Consistent with previous literature [28, 29, 31], gene expression analysis revealed that IDO was the most highly induced gene in MSC-*γ* and may be the key candidate molecule by which MSC-*γ* mediate enhanced T cell immunosuppression [1].

MSC constitutively express a range of TLRs, including TLR3 and TLR4 [32, 33]. Activation of TLR3 and (or) TLR4 amplifies MSC trophic factors, antimicrobial activity, and immunosuppressive potential, thereby enhancing MSC therapeutic potency [33–36]. Both TLR3 and TLR4 were upregulated in MSC-*γ* compared to UT-MSC. Activation of TLR3 and TLR4 signaling with poly I:C or LPS, respectively, induced IDO expression in MSC [33]. TLR-driven induction of IDO in MSC resulted in the degradation of tryptophan and production of immunosuppressive kynurenines [33]. TLR3 activation has also been linked to expression of IL-6 in MSC [34]. IL-6 mediates the inhibitory effects of MSC on DC differentiation, maturation, and function [37, 38]. Consistent with our previous report, the upregulation of IL-6 transcripts in MSC-*γ* and high protein concentrations of IL-6 in MSC-*γ*-T cell coculture supernatants suggest that MSC-*γ* secreted IL-6 may be involved in suppression of proinflammatory T cell responses [1]. Nevertheless, TLR activation in MSC is known to abrogate their immunosuppressive properties [39, 40]. The effects of TLR signaling in MSC are still not fully understood and remain to be further investigated.

TLR3/4 preactivated MSC have enhanced leukocyte binding activity mediated by the induction of the adhesion molecule ICAM-1, consistent with upregulation of ICAM-1 in MSC-*γ* [35]. ICAM-1 together with TLR3 and TLR4 were among the genes enriched for the gene ontology term positive regulation of immune system process, suggesting a potential biological role of TLR3/4 in MSC-*γ* induction of ICAM-1 [35]. Additionally, the upregulation of chemokines such as CXCL9, CXCL10, CXCL11, CXCL16, CCL2, CCL8, and CCL13 detected by microarray may facilitate T cell recruitment to MSC-*γ*. Mouse MSC preactivated with IFN-*γ* and TNF-*α* induced CXCL9 and CXCL10 [41]. The production of these chemokines was abrogated by IFN-*γ* neutralization [41]. Moreover, the blockade of CXCR3, a T cell receptor for chemokines CXCL9 and CXCL10, eliminated T cell chemotaxis towards MSC and subsequent MSC inhibition of T cell proliferation [41]. These studies concluded that cytokines induce MSC-expression of chemokines to drive T cell recruitment into close proximity with MSC, enabling MSC to suppress T cells through the secretion of immunosuppressive molecules [41, 42]. Chemokines also increased the in vivo migratory properties of human MSC-*γ* to sites of inflammation in colitis mouse models [43]. Studies to validate the functional role of human MSC-*γ* derived chemokines and ICAM-1 in the recruitment and subsequent modulation of T cell responses as well as in MSC-*γ* homing to sites of inflammation in vivo are required. Hence, IFN-*γ* directly induces an array of immunosuppressive molecules in MSC and may further amplify the secretion of other MSC-inhibitory molecules such as IDO, IL-6, and ICAM-1 via TLR3/4 activation. MSC-*γ* with higher proximity to leukocytes may serve as an additional mechanism by which MSC-*γ* increase their modulatory activity on T cells.

Despite being highly immunosuppressive with enhanced homing and reparative capacities, allogeneic MSC-*γ* are ineffective in vivo due to their increased immunogenicity [44–46]. A large number of genes upregulated in MSC-*γ* were involved in the antigen processing and presentation pathways of MHC classes I and II. Antigen processing and presentation occurs via the cytosolic [47–51] or endocytic pathways [51, 52]. In the cytosolic pathway, degraded intracellular proteins are transported to the rough endoplasmic reticulum (RER) via TAP, a heterodimer consisting of TAP1 and TAP2. These peptides are further trimmed by aminopeptidases ERAP to enable optimal peptide loading onto MHC class I molecules. MHC class I components comprise the class I MHC *α*-chain and the B2M chain. This MHC class I molecule associates with the chaperone molecules tapasin, calreticulin, and ERp57. Tapasin (TAPBPL) recruits the MHC I molecules into proximity to TAP, allowing efficient peptide loading onto MHC class I molecules, subsequently stabilizing the peptide-class I molecule complex. The class I MHC-peptide complex is then transported to the plasma membrane for antigen-peptide presentation to CD8^+^ T cells [47–51]. Induction of ERAP, TAP2, TAPBPL, and B2M, genes involved in this cytosolic pathway was evident in MSC-*γ* and correlated with the observed upregulation of MHC class I in these cells [1]. We have also shown that MHC class II is induced in MSC-*γ* [1]. In the endocytic pathway, assembly of MHC class II occurs in RER where the *α*- and *β*-chain associate and this newly synthesised class II MHC complex binds to the invariant chain (Ii, CD74). As MHC class II-Ii complex is translocated into the endosomal compartment, the Ii chain is degraded, leaving the CLIP fragment (class II associated Ii peptide) bound to the MHC II peptide binding cleft. HLA-DM catalyses the exchange of CLIP with the antigenic peptide. The MHC class II peptide complex is then transported to the plasma membrane for antigen presentation to CD4^+^ T cells [51, 52]. We detected high expression of CD74 and HLA-DM in MSC-*γ*, supporting the induction of MHC class II on these cells. Alloimmune responses against UT-MSC are mediated by the recognition of allogeneic MHC molecules by recipient CD4^*+*^ and CD8^*+*^ memory T cells [53]. MHC class II expression on allogeneic MSC is also known to induce alloimmune responses in cocultures with MHC-mismatched responder cells [54]. Therefore, the amplification of this antigen processing and presentation machinery suggests that MSC-*γ* are highly immunogenic and can potently prime proinflammatory T cell responses in allogeneic hosts.

Moreover, MSC-*γ* were enriched for gene sets involved in augmentation of the humoral immunity and complement pathways activation. Our data may explain previously published data, where MSC-*γ* infused mice had higher levels of circulating anti-donor IgM and IgG alloantibodies, which resulted in the rapid induction of antibody-mediated rejection [44]. Although MSC-*γ* lack the expression of costimulatory signals (CD80, CD83, and CD86) to function as APC to mediate direct T cell allorecognition and activation [1, 55–58], we speculate that MSC-*γ* induce allogeneic T cell responses through the indirect or semidirect pathways of allorecognition [21, 59]. Allogeneic MHC-peptide transfer from MSC-*γ* could be more rapid compared to UT-MSC due to high expression of MHC molecules. This enables allogeneic MHC-peptide to be recognised by recipient T cells through the semidirect pathway. Understanding mechanisms of MSC-*γ* immunogenicity may enable the targeting of MSC through different pathways of activation to increase their immunomodulatory function whilst retaining MSC in a nonimmunogenic and inert state.

Human MSC-17 showed superior suppression of T cell responses and were able to induce Tregs with minimal immunogenicity [1]. MSC constitutively express a range of MMPs including MMP2, membrane type 1 MMP (MTI-MMP), tissue inhibitor of MMP1 (TIMP1), and TIMP2 [60–62]. These MMPs are essential for MSC invasion and migration across the extracellular matrix (ECM) as demonstrated in in vitro transendothelial migration assays [62]. Absence of MMPs impairs MSC transmigration capacity across Matrigels [60–62]. In response to IL-1*β* and TNF-*α*, MSC have also been shown to amplify the expression of MMP2, MTI-MMP, and (or) MMP9 in MSC, thereby promoting MSC invasiveness across the basement membrane [60]. Here, we demonstrated that IL-17A induced the gene expression of MMP13 and MMP1 in MSC. These MMPs were highly enriched for collagen degradation and metabolic processes, suggesting that these factors may be essential for MSC-17 to invade the ECM.

MSC-derived MMPs also have proteolytic activity on chemokines [63, 64]. MMP processing of CC chemokines convert the biochemical properties of the chemokine target molecules from an agonist to an antagonist form with anti-inflammatory properties in vivo [65]. MSC-derived MMP1 cleaves CCL2, leading to the generation of CCL2 with suppressive properties on B cell production of immunoglobulins and in CD4^+^ T cell activation [63, 64]. We showed that MMP1, CCL2, and MMP13 were upregulated in human MSC-17. Evaluating the functional role of MMP-processed chemokine derivatives in MSC-17 immunomodulation on T cells in this study remains to be elucidated.

MMP-2 and MMP-9 secreted by MSC are known to cleave and reduce CD25 expression on T cells, thus impairing T cell activation and proliferation [66]. Administration of MMP inhibitors in an islet allotransplant model abrogated the suppressive effect of MSC on alloreactive T cells, resulting in allograft rejection. This study concluded that MMPs are crucial for MSC immunosuppression [66]. We have previously shown that MSC-17 further downregulated CD25 expression on CD4^+^ effector T cells compared to UT-MSC, a process partially mediated by cell contact dependent mechanisms [1]. The involvement of MSC-17-derived MMP13 and MMP1 in downregulating CD25 on T cells has not been previously established. Blocking MMP13 activity using specific inhibitors may provide insights on its role in inhibiting T cell activation.

Apart from the upregulation of chemotactic transcripts, MSC-17 were also enriched for genes involved in wound healing and angiogenesis. Tissue plasminogen activator (PLAT) was upregulated in human bone marrow derived MSC-17 when compared to MSC-*γ*. PLAT was enriched for biological processes involving cell motility, angiogenesis, and responses to wounding. A previous study reported that IL-17A can increase MSC migration in an in vitro wound healing assay [7]. In a latter study, IL-17A was shown to enhance peripheral blood-derived MSC migration in a wound healing assay by inducing the expression of the urokinase type plasminogen activator through the activation of ERK1,2-MAPK signaling pathway [67]. Increased expression of the urokinase type plasminogen activator has been reported to facilitate MSC transendothelial migration, potentially contributing to MSC motility to sites of inflammation for tissue regeneration or immunosuppression [67]. In other studies, tissue plasminogen activators have also shown to support angiogenesis by promoting vascular endothelial cell migration to ischemic regions [68, 69]. These data suggest that MSC-17 in addition to their potent immunosuppressive properties may benefit disease conditions of ischemia injury that require tissue repair and angiogenesis.

In this study, donor to donor variation may have limited the robustness of our microarray data to detect subtle changes in MSC-17 gene expression profile. However, real-time PCR data validated changes detected in the highly regulated genes in MSC-17. More MSC-17 biological replicates may provide further insights into other genes that are differently regulated.

## 5. Conclusions

Enhanced expression of MHC in allogeneic MSC-*γ* increases their immunogenicity and this may negatively impact MSC-*γ* potency in vivo. Nevertheless, we have highlighted novel candidate immunosuppressive molecules and pathways in which MSC-*γ* can be targeted in future studies to increase the immunomodulatory capacity of MSC. We have also identified a few novel candidate molecules that may contribute to the potent MSC-17 regulation of immune responses. These candidate molecules can be explored for their regulatory roles in MSC-17 suppression of T cell responses and in the generation of Tregs in future studies.

## Supplementary Material

Table S1, upregulated genes in MSC-γ vs. UT-MSC.
Table S2, downregulated genes in MSC-γ vs. UT-MSC.
Table S3, gene ontology terms for biological process of upregulated MSC-γ vs. UT-MSC genes.
Table S4, gene ontology terms for biological process of downregulated MSC-γ vs. UT-MSC genes.
Table S5, gene ontology terms for molecular functions of upregulated MSC-γ vs. UT-MSC genes.
Table S6, gene ontology terms for molecular functions of downregulated MSC-γ vs. UT-MSC genes.
Table S7, gene ontology terms for cellular components of upregulated MSC-γ vs. UT-MSC genes.
Table S8, gene ontology terms for cellular components of downregulated MSC-γ vs. UT-MSC genes.
Table S9, unmapped genes from the gene list entry for DAVID: MSC-17 vs. UT-MSC genes.
Table S10, gene enrichment analysis of MSC-17 vs. UT-MSC.
Table S11, gene ontology terms for biological processes of MSC-17 vs. UT-MSC.
Table S12, gene ontology terms for molecular functions: MSC-17 vs. UT-MSC.
Table S13, gene ontology terms for cellular components: MSC-17 vs. UT-MSC.
Table S14, gene ontology terms for biological process of upregulated MSC-17 vs. MSC-γ genes.
Table S15, gene ontology terms for biological process of downregulated MSC-17 vs. MSC-γ genes.
Table S16, gene ontology terms for molecular functions of upregulated MSC-17 vs. MSC-γ genes.
Table S17, gene ontology terms for cellular components of upregulated MSC-17 vs. MSC-γ genes.
Table S18, gene ontology terms for cellular components of downregulated MSC-17 vs. MSC-γ genes.

## Figures and Tables

**Table 1:** Top 30 differentially expressed genes: MSC-*γ* versus UT-MSC.

Gene symbol	Gene name	mRNA Accession	Fold change	*p* value
*Upregulated genes*				
HLA-DRA	Major histocompatibility complex, class II, DR alpha	NM_019111	387.78	0.00049
GBP4	Guanylate binding protein 4	NM_052941	199.41	0.00002
IDO1	Indoleamine 2,3-dioxygenase 1	NM_002164	96.72	0.00003
HLA-DRB	Major histocompatibility complex, class II, DR beta	ENST00000307137	89.67	0.00435
GBP5	Guanylate binding protein 5	NM_052942	88.07	0.00003
CXCL9	Chemokine (C-X-C motif) ligand 9	NM_002416	83.60	0.00002
GBP2	Guanylate binding protein 2, interferon-inducible	ENST00000464839	77.00	0.00004
SECTM1	Secreted and transmembrane 1; NULL	NM_003004	57.59	0.00002
HLA-DRB3	Major histocompatibility complex, class II, DR beta 3	ENST00000426847	51.65	0.00868
CIITA	Class II, major histocompatibility complex, transactivator	NM_00024	38.84	0.00003
GBP1	Guanylate binding protein 1, interferon-inducible	NM_002053	29.09	0.00001
RP11-44K6.2	NULL	ENST00000520185	26.39	0.00060
GCH1	GTP cyclohydrolase 1	NM_000161	24.93	0.00022
USP30-AS1	USP30 antisense RNA 1	ENST00000478808	24.75	0.00009
GBP2	Guanylate binding protein 2, interferon-inducible; NULL	NM_004120	23.60	0.00001
HLA-DOA	Major histocompatibility complex, class II, DO alpha; NULL	NM_002119	22.90	0.00003
IFIT3	Interferon-induced protein with tetratricopeptide repeats 3	NM_001031683	21.49	0.00013
FAM129A	Family with sequence similarity 129, member A	NM_052966	20.89	0.00003
CTSS	Cathepsin S	NM_004079	20.10	0.00002
SLC7A11	Solute carrier family 7 (anionic amino acid transporter light chain, xc- system), member 11	NM_014331	19.70	0.00009
IRF1	Interferon regulatory factor 1	NM_002198	19.55	0.00002
CD74	CD74 molecule, major histocompatibility complex, class II invariant chain; NULL	NM_001025159	18.49	0.00060
ICAM1	Intercellular adhesion molecule 1	NM_000201	18.43	0.00003
HCP5	HLA complex P5 (nonprotein coding); NULL	ENST00000457127	18.15	0.00032
LGALS17A	Charcot-Leyden crystal protein pseudogene	ENST00000412609	18.12	0.00038
PARP14	Poly (ADP-ribose) polymerase family, member 14	NM_017554;	17.05	0.00014
RARRES3	Retinoic acid receptor responder (tazarotene induced) 3	NM_004585	17.00	0.00007
WARS	Tryptophanyl-tRNA synthetase; NULL	NM_004184	16.49	0.00002
IFIT2	Interferon-induced protein with tetratricopeptide repeats 2	NM_001547	16.43	0.00051
TMEM140	transmembrane protein 140	NM_018295	16.08	0.00012
*Downregulated genes*				
LRRC15	Leucine rich repeat containing 15	NM_001135057	−19.91	0.0007
KIAA1199	KIAA1199; NULL	NM_018689	−13.26	0.0025
RNU5A-8P	RNA, U5A small nuclear 8, pseudogene	ENST00000364102	−12.36	0.0061
COL10A1	Collagen, type X, alpha 1	NM_000493;	−12.25	0.0031
COL3A1	Collagen, type III, alpha 1; microRNA 3606	NM_000090	−11.88	0.0000
HIST1H2A	Histone cluster 1, H2ai; histone cluster 1, H2ah; histone cluster 1, H2ag; histone cluster 1, H2am; histone cluster 1, H2al; histone cluster 1, H2ak; histone cluster 1, H3f	NM_003509	−11.67	0.0012
SCD	Stearoyl-CoA desaturase (delta-9-desaturase)	NM_005063	−9.75	0.0000
U2	U2 spliceosomal RNA	ENST00000410792	−9.41	0.0476
HIST1H3	Histone cluster 1, H3b; histone cluster 1, H3f; histone cluster 1, H3h; histone cluster 1, H3j; histone cluster 1, H3g; histone cluster 1, H3i; histone cluster 1, H3e; histone cluster 1, H3c; histone cluster 1, H3d; histone cluster 1, H3a	NM_003537	−9.06	0.0053
HIST1H1B	Histone cluster 1, H1b	NM_00532	−8.53	0.0002
—	—	ENST00000408768	−8.25	0.0001
KDELR3	KDEL (Lys-Asp-Glu-Leu) endoplasmic reticulum protein retention receptor 3	NM_016657	−8.22	0.0007
—	—	BC091525	−7.87	0.0007
SNORD114-11	Small nucleolar RNA, C/D box 114-11	NR_003204	−7.33	0.0021
WISP1	WNT1 inducible signaling pathway protein 1	NM_003882	−7.18	0.0000
U3	Small nucleolar RNA U3	ENST00000390893	−7.14	0.0128
HIST1H2BM	Histone cluster 1, H2bm	NM_003521	−6.92	0.0024
COL1A1	Collagen, type I, alpha 1; NULL	NM_000088	−6.84	0.0000
HIST1H3	histone cluster 1, H3g; histone cluster 1, H3f; histone cluster 1, H3b; histone cluster 1, H3h; histone cluster 1, H3j; histone cluster 1, H3i; histone cluster 1, H3e; histone cluster 1, H3c; histone cluster 1, H3d; histone cluster 1, H3a	NM_003534	−6.68	0.0032
HIST1H3	Histone cluster 1, H3f; histone cluster 1, H3b; histone cluster 1, H3h; histone cluster 1, H3j; histone cluster 1, H3g; histone cluster 1, H3i; histone cluster 1, H3e; histone cluster 1, H3c; histone cluster 1, H3d; histone cluster 1, H3a	NM_021018	−6.41	0.0058
AL732479.1	—	ENST00000459197	−6.38	0.0015
ADAM12	ADAM metallopeptidase domain 12; NULL	NM_003474;	−6.13	0.0001
ENPP1	Ectonucleotide pyrophosphatase/phosphodiesterase 1	NM_006208	−6.06	0.0002
NDNF	Neuron-derived neurotrophic factor	NM_024574	−6.00	0.0100
DHCR7	7-Dehydrocholesterol reductase; NULL	NM_001360	−5.88	0.0005
DHCR24	24-Dehydrocholesterol reductase	NM_014762	−5.84	0.0001
RGS4	Regulator of G-protein signaling 4; NULL	NM_001102445	−5.78	0.0116
CRABP2	Cellular retinoic acid binding protein 2	NM_001878	−5.76	0.0015
KIF20A	Kinesin family member 20A; NULL	NM_005733	−5.60	0.0072
U1	U1 spliceosomal RNA	—	−5.38	0.0069

**Table 2:** Differentially expressed genes (mapped by DAVID): MSC-17 versus UT-MSC.

Gene symbol	Gene name	mRNA Accession	Fold change	*p* value
*Upregulated genes*				
MMP13	Matrix metallopeptidase 13 (collagenase 3)	NM_002427	15.60	0.0021
C3	Complement component 3; NULL	NM_000064	11.56	0.0039
LBP	Lipopolysaccharide binding protein	NM_004139	5.35	0.0031
VMO1	Vitelline membrane outer layer 1 homolog (chicken)	NM_182566	4.07	0.0022
CH25H	Cholesterol 25-hydroxylase	NM_003956	3.99	0.0023
IL6	Interleukin 6 (interferon, beta 2); NULL	NM_000600	3.44	0.0083
ZC3H12A	Zinc finger CCCH-type containing 12A	NM_025079	3.09	0.0010
CCL2	Chemokine (C-C motif) ligand 2	NM_002982	3.08	0.0405
ZNF253	Zinc finger protein 253	NM_021047	2.82	0.0010
SAA1	Serum amyloid A1	NM_000331	2.72	0.0102
CXCL6	Chemokine (C-X-C motif) ligand 6	NM_002993	2.44	0.0014
MMP1	Matrix metallopeptidase 1 (interstitial collagenase)	NM_002421	2.40	0.0356
NFKBIZ	Nuclear factor of kappa light polypeptide gene enhancer in B-cells inhibitor, zeta; NULL	NM_031419	2.36	0.0232
MIRLET7A2	MicroRNA let-7a-2	NR_029477	2.30	0.0031
RBMY2EP	RNA binding motif protein, Y-linked, family 2, member E pseudogene	ENST00000444169	2.27	0.0278
CCL8	Chemokine (C-C motif) ligand 8	NM_005623	2.20	0.0012
STC1	Stanniocalcin 1	NM_003155	2.20	0.0023
SFRP4	Secreted frizzled-related protein 4	NM_003014	2.19	0.0136
SLC22A3	Solute carrier family 22 (extraneuronal monoamine transporter), member 3	NM_021977	2.15	0.0452
TTTY11	Testis-specific transcript, Y-linked 11 (nonprotein coding)	NR_001548	2.15	0.0252
STEAP2	STEAP family member 2, metalloreductase; NULL	NM_001244944	2.12	0.0225
SCARNA18	Small Cajal body-specific RNA 18	NR_003139	2.06	0.0254
LOC100287834	Uncharacterised LOC100287834	NR_028349	2.06	0.0328
*Downregulated genes*				
RPS24	Ribosomal protein S24; NULL	NM_001142285	−2.01	0.0209
LOC100133299	GALI1870	AY358688	−2.03	0.0095
POU5F1	POU class 5 homeobox 1	ENST00000259915	−2.04	0.0129
TMEM171	Transmembrane protein 171	NM_173490	−2.05	0.0133
IGLJ2	Immunoglobulin lambda joining 2	ENST00000390322	−2.07	0.0252
ITGA6	Integrin, alpha 6; NULL	ENST00000264107	−2.11	0.0109
RNU7-25P	RNA, U7 small nuclear 25 pseudogene; RNA, U7 small nuclear 11 pseudogene	ENST00000516544	−2.16	0.0047
GTF2IRD2B	GTF2I repeat domain containing 2B	NM_001003795	−2.20	0.0040
SERTAD4	SERTA domain containing 4	ENST00000367012	−2.29	0.0470
TPTE	Transmembrane phosphatase with tensin homology	ENST00000415664	−2.85	0.0128

**Table 3:** Top 30 differentially expressed genes: MSC-17 versus MSC-*γ*.

Gene symbol	Gene name	mRNA Accession	Fold change	*p* value
*Upregulated genes*				
MMP13	Matrix metallopeptidase 13 (collagenase 3)	NM_002427	24.27	0.0009
HIST1H2AI	Histone cluster 1, H2ai	NM_003509	17.44	0.0005
U3	Small nucleolar RNA U3	ENST00000390893	13.96	0.0118
ZNF25	Zinc finger protein 25	ENSG00000175395	13.66	0.0008
LRRC15	Leucine rich repeat containing 15	NM_001135057	13.57	0.0005
HIST1H3G	Histone cluster 1, H3g	NM_003534	12.68	0.0008
SNORD114-11	Small nucleolar RNA, C/D box 114-11	NR_003204	12.33	0.0386
HIST1H3B	Histone cluster 1, H3b	NM_003537	11.20	0.0064
U1	U1 spliceosomal RNA	NONHSAT054977	10.54	0.0017
HIST1H1B	Histone cluster 1, H1b	NM_005322	10.28	0.0003
SCD	Stearoyl-CoA desaturase (delta-9-desaturase)	NM_005063	10.15	0.0008
KRT16P4	Keratin 16 pseudogene 4	ENST00000453883	9.11	0.0122
ADAM12	ADAM metallopeptidase domain 12	NM_001288973	8.96	0.0031
HIST1H2BM	Histone cluster 1, H2bm	NM_003521	8.87	0.0125
HIST1H3F	Histone cluster 1, H3f	NM_021018	8.77	0.0081
ADAM12	ADAM metallopeptidase domain 12	NM_001288973	8.70	0.0121
KIAA1199	KIAA1199; NULL	NM_018689	8.48	0.0045
COL10A1	Collagen, type X, alpha 1	NM_000493	6.87	0.0136
DHCR7	7-Dehydrocholesterol reductase; NULL	NM_001360	6.51	0.0022
P4HA3	Prolyl 4-hydroxylase, alpha polypeptide III	NM_182904	6.49	0.0043
LBP	Lipopolysaccharide binding protein	NM_004139	6.11	0.0021
HAS1	Hyaluronan synthase 1	NM_001523	6.10	0.0038
COL1A1	Collagen, type I, alpha 1; NULL	NM_000088	5.94	0.000002
NDNF	Neuron-derived neurotrophic factor	NM_024574	5.75	0.0147
ELN	Elastin; NULL	NM_000501	5.65	0.0014
WISP1	WNT1 inducible signaling pathway protein 1	NM_003882	5.61	0.0052
ADAM12	ADAM metallopeptidase domain 12; NULL	NM_003474	5.56	0.0008
KDELR3	KDEL (Lys-Asp-Glu-Leu) endoplasmic reticulum protein retention receptor 3	NM_016657	5.36	0.0085
HIST1H3I	Histone cluster 1, H3i	NM_003533	5.02	0.0014
CNN1	Calponin 1, basic, smooth muscle	NM_001299	4.69	0.00001
*Downregulated genes*				
HLA-DRA	Major histocompatibility complex, class II, DR alpha	NM_019111	−553.64	0.0005
GBP4	Guanylate binding protein 4	NM_052941	−244.27	0.0001
HLA-DRA	Major histocompatibility complex, class II, DR alpha; NULL	ENST00000442960	−188.10	0.00005
CXCL9	Chemokine (C-X-C motif) ligand 9	NM_002416	−96.44	0.00001
IDO1	Indoleamine 2,3-dioxygenase 1	NM_002164	−76.98	0.00004
HLA-DRB3	Major histocompatibility complex, class II, DR beta 3	ENST00000307137	−70.87	0.0024
GBP5	Guanylate binding protein 5	NM_052942	−65.02	0.000004
SECTM1	Secreted and transmembrane 1; NULL	NM_003004	−50.74	0.0001
GBP2	Guanylate binding protein 2, interferon-inducible	ENST00000464839	−41.55	0.00001
HLA-DPA1	Major histocompatibility complex, class II, DP alpha 1	NM_001242524	−40.79	0.0011
IFIT3	Interferon-induced protein with tetratricopeptide repeats 3	NM_001031683	−39.52	0.0011
CIITA	Class II, major histocompatibility complex, transactivator	NM_000246	−37.92	0.000003
GBP1	Guanylate binding protein 1, interferon-inducible	NM_002053	−35.79	0.0002
PSAT1	Phosphoserine aminotransferase 1	NM_021154	−31.20	0.0001
HLA-DPB1	NULL	OTTHUMT00000310634	−28.81	0.00002
GBP1P1	Guanylate binding protein 1, interferon-inducible pseudogene 1	ENST00000513638	−27.87	0.00001
GCH1	GTP cyclohydrolase 1	NM_000161	−26.74	0.0002
PARP14	Poly (ADP-ribose) polymerase family, member 14	NM_017554	−26.49	0.0008
IRF1	Interferon regulatory factor 1	NM_002198	−24.92	0.000003
HLA-DOA	Major histocompatibility complex, class II, DO alpha	NM_002119	−24.00	0.00001
RARRES3	Retinoic acid receptor responder (tazarotene induced) 3	NM_004585	−23.10	0.0001
LGALS17A	Charcot-Leyden crystal protein pseudogene	ENST00000412609	−22.45	0.0003
HLA-DOA	Major histocompatibility complex, class II, DO alpha	NM_002119	−21.85	0.00001
SLC7A11	Solute carrier family 7 (anionic amino acid transporter light chain, xc- system), member 11	NM_014331	−21.62	0.00004
USP30-AS1	USP30 antisense RNA 1	ENST00000478808	−20.00	0.0001
HCP5	HLA complex P5 (non-protein coding); NULL	ENST00000457127	−19.90	0.0001
ICAM1	Intercellular adhesion molecule 1	NM_000201	−19.63	0.0001
WARS	Tryptophanyl-tRNA synthetase; NULL	NM_004184	−19.41	0.0001
APOL1	Apolipoprotein L, 1; NULL	NM_003661	−19.36	0.00002
ERVK-7	Endogenous retrovirus group K, member 7; novel transcript	ENST00000522373	−18.93	0.0267
HLA-DPB2	Major histocompatibility complex, class II, DP beta 2 (pseudogene)	NR_001435	−18.91	0.00009
