# Supplementary material for: Transcriptome Profiling of IL-17A Preactivated Mesenchymal Stem Cells: A Comparative Study to Unmodified and IFN-γ Modified Mesenchymal Stem Cells
Source: Stem Cells Int. 2017 Feb 15;2017:1025820. doi: 10.1155/2017/1025820 (PMC5331321; doi:10.1155/2017/1025820)
Supplement: Supplementary file 1 — Table S1, upregulated genes in MSC-γ vs. UT-MSC. Table S2, downregulated genes in MSC-γ vs. UT-MSC. Table S3, gene ontology terms for biological process of upregulated MSC-γ vs. UT-MSC genes. Table S4, gene ontology terms for biological process of downregulated MSC-γ vs. UT-MSC genes. Table S5, gene ontology terms for molecular functions of upregulated MSC-γ vs. UT-MSC genes. Table S6, gene ontology terms for molecular functions of downregulated MSC-γ vs. UT-MSC genes. Table S7, gene ontology terms for cellular components of upregulated MSC-γ vs. UT-MSC genes. Table S8, gene ontology terms for cellular components of downregulated MSC-γ vs. UT-MSC genes. Table S9, unmapped genes from the gene list entry for DAVID: MSC-17 vs. UT-MSC genes. Table S10, gene enrichment analysis of MSC-17 vs. UT-MSC. Table S11, gene ontology terms for biological processes of MSC-17 vs. UT-MSC. Table S12, gene ontology terms for molecular functions: MSC-17 vs. UT-MSC. Table S13, gene ontology terms for cellular components: MSC-17 vs. UT-MSC. Table S14, gene ontology terms for biological process of upregulated MSC-17 vs. MSC-γ genes. Table S15, gene ontology terms for biological process of downregulated MSC-17 vs. MSC-γ genes. Table S16, gene ontology terms for molecular functions of upregulated MSC-17 vs. MSC-γ genes. Table S17, gene ontology terms for cellular components of upregulated MSC-17 vs. MSC-γ genes. Table S18, gene ontology terms for cellular components of downregulated MSC-17 vs. MSC-γ genes. [file 1025820.f1.docx]

# SUPPLEMENTARY TABLES

Table S1 Upregulated genes (unmapped by DAVID): MSC- γ vs. UT-MSC

| **Upregulated** | **Upregulated (continued)** | **Upregulated (continued)** |
| --- | --- | --- |
| P4-620F22.2 | MIR2909 | LOC100505573 |
| MIR4442 | LOC100653057 | HIF1A-AS2 |
| OTTHUMG00000164188 | ZFAS1 | Y_RNA.512-201 |
| LOC101060720 | AJUBA | LOC101060449 |
| ARL14EP | LOC100505633 | DLGAP1-AS2 |
| RP11-44K6.2 | RNF185-AS1 | FAM86JP |
| HLA-DM | snoU13 | AC089987.2 |
| MIR4751 | LRIF1 | lnc-EXT1-2 |
| RP1-102E24.8 | RP4-791M13.3 | LOC100505794 |
| C2CD5 | OTTHUMG00000169784 | LOC100507419 |
| LY75-CD302 | LOC100507449 |  |
| AK123300 | NPIPA5 |  |
| GBP1P1 | LINC00265 |  |
| SLC35F6 | CERS6 |  |
| MIR3614 | CCP110 |  |
| ARHGEF28 | MIR3689F |  |
| XXbac-BPG116M5.17 | LINC00965 |  |
| RNU7-40P | MIR3189 |  |
| ZFAND4 | FOPNL |  |
| OTTHUMG00000168357 | LURAP1L |  |
| NPIPB3 | RP11-274J7.2 |  |
| LOC100509976 | RP11-638I2.8 |  |
| lnc-GLI3-1 | RP11-468E2.5 |  |
| NPIPA1 | NPIPB5 |  |
| TMEM9B-AS1 | LOC101060503 |  |
| lnc-ADCY9-1 | PS1TP5 |  |
| RNU4-9P | AC108676.1 |  |
| RNU6-336P-201 | RP11-44K6.4 |  |
| Y_RNA | HLA-DRB |  |
| SMG1P1 | SUZ12P1 |  |
| NPIPB11 | USP30-AS1 |  |
| LOC100507516 | linc-SLC9A3-2 |  |
| AC023818.2 | PSMG3-AS1 |  |
| LOC100506544 | EPB41L4A-AS1 |  |
| OTTHUMG00000159600 | lnc-TEKT1-1 |  |
| FOXN3-AS1 | LGALS17A |  |
| PLCE1-AS1 | RP11-841O20.2 |  |
| OTTHUMG00000168751 | AC005161.1 |  |
| GSAP | CTA-384D8.31 |  |
| RNU6-620P-201 | HLA-DQA |  |

Table S2 Downregulated genes (unmapped by DAVID): MSC- γ vs. UT-MSC

| **Downregulated** | **Downregulated (continued)** |
| --- | --- |
| RP11-94A24.1 | RNU5E-1 |
| AC093850.2 | RNU5A-8P |
| U3 | OSTCP2 |
| NDNF | OTTHUMG00000165130 |
| RNA5SP418 | RP13-631K18.5 |
| RP11-283C24.1 | AC003968.1 |
| LEPREL4 | RP11-160E2.6 |
| RN5S475 | 7SK |
| MIR323A | AL596092.1 |
| ANKRD18CP | RP13-582L3.4 |
| SNORD3 | MIR4649 |
| OTTHUMG00000154838 | AC092143.2 |
| Y_RNA | OTTHUMG00000175982 |
| MMP24-AS1 | AL732479.1 |
| MIR4532 | RN5S203 |
| OTTHUMG00000165727 | AC116154.1 |
| U2 | HIST1H3 |
| U6 | OTTHUMG00000170901 |
| ADIRF | U1 |
| U3 | MSMO1 |
| SNORA35 | LOC100506188 |
| KRTAP2 | IFITM10 |
| HIST1H4 | OTTHUMG00000074741 |
| AL121652.2 | HIST1H2A |
| OTTHUMG00000178158 |  |
| AC108865.1 |  |
| RP11-939C17.2 |  |
| MBNL1-AS1 |  |
| LOC100505502 |  |
| AP001422.1 |  |
| AC125238.4 |  |
| snoU13 |  |
| PRADC1 |  |
| MIR4327 |  |
| RP4-541C22.4 |  |
| HIST2H3 |  |
| U1 |  |
| HIST1H2B |  |

Table S3. Functional annotation clustering - Gene Ontology Terms for Biological Processes: MSC-γ vs. UT-MSC (upregulated genes)

| **Term** | **Count** | **Genes** | **P-Value** | **Fold Enrichment** |
| --- | --- | --- | --- | --- |
| **Annotation Cluster 1 Enrichment Score: 8.03** | | | | |
| GO:0019882~antigen processing and presentation | 25 | HLA-DRB3, IFI30, HLA-DMB, CD74, B2M, TAP2, ERAP1, ERAP2, HLA-DPB1, HLA-DOA, ICAM1, HLA-L, HLA-A, HLA-C, HLA-B, HLA-E, HLA-DQA2, HLA-G, HLA-DQA1, PSMB9, HLA-F, PSME1, ULBP1, HLA-DPA1, TAPBPL, HLA-DRA | 5.59E-17 | 9.37 |
| GO:0048002~antigen processing and presentation of peptide antigen | 14 | HLA-A, IFI30, HLA-C, HLA-B, HLA-E, HLA-G, CD74, B2M, HLA-F, TAP2, ERAP1, ERAP2, HLA-DOA, TAPBPL, HLA-DRA | 7.71E-13 | 15.55 |
| GO:0002474~antigen processing and presentation of peptide antigen via MHC class I | 10 | TAP2, HLA-A, ERAP1, HLA-C, ERAP2, HLA-B, HLA-E, TAPBPL, HLA-G, B2M, HLA-F | 6.42E-10 | 18.29 |
| GO:0019883~antigen processing and presentation of endogenous antigen | 5 | TAP2, ERAP1, ERAP2, TAPBPL, CD74 | 6.60E-05 | 19.44 |
| GO:0002483~antigen processing and presentation of endogenous peptide antigen | 4 | TAP2, ERAP1, ERAP2, TAPBPL | 6.10E-04 | 20.73 |
| GO:0019885~antigen processing and presentation of endogenous peptide antigen via MHC class I | 4 | TAP2, ERAP1, ERAP2, TAPBPL | 6.10E-04 | 20.73 |
| **Annotation Cluster 2 Enrichment Score: 6.06** | | | | |
| GO:0006952~defense response | 55 | TLR3, TLR4, CXCL11, CXCL10, HCP5, MX1, MX2, CIITA, SP100, C4A, LY96, C4B, SCUBE1, HLA-C, SERPING1, HLA-B, HLA-G, RIPK2, HLA-DRA, IFIH1, CCL2, NMI, C3, CLU, CXCL9, RSAD2, CCL8, CALCOCO2, C1R, IL32, C1S, CD74, GCH1, LGALS3BP, TAP2, BCL2, TAP1, C2, PTX3, ITK, IL18R1, IL6, CEBPB, CFB, CEBPG, SAMHD1, MALT1, IDO1, APOL2, DDX58, APOL3, CCL13, APOL1, NUPR1, CXCL16, C1RL | 1.32E-11 | 2.78 |
| GO:0045087~innate immune response | 24 | CIITA, IL18R1, IFIH1, SP100, C4A, CFB, C4B, C3, CEBPG, CLU, TLR3, CALCOCO2, SAMHD1, MALT1, SERPING1, C1R, TLR4, C1S, GCH1, DDX58, APOL1, CXCL16, C1RL, C2 | 7.52E-11 | 5.41 |
| GO:0002252~immune effector process | 21 | ICAM1, IL6, NBN, C4A, CFB, C4B, C3, CEBPG, CLU, RSAD2, SAMHD1, SERPING1, C1R, C1S, CD74, TNFSF13B, BCL2, C1RL, C2, PTX3, HLA-DRA | 9.55E-09 | 4.87 |
| GO:0048584~positive regulation of response to stimulus | 28 | C3, IL6ST, CLU, TLR3, C1R, TLR4, C1S, TGFB2, B2M, EDNRA, TAP2, C2, IL6, C4A, CFB, C4B, CEBPG, EDA2R, MALT1, SERPING1, IDO1, TNFSF13B, HIPK2, VEGFA, C1RL, RIPK2, JAK2, HLA-DRA | 9.91E-09 | 3.69 |
| GO:0002684~positive regulation of immune system process | 28 | C3, IL6ST, CLU, TLR3, C1R, TLR4, C1S, CD74, B2M, TGFB2, CD47, TAP2, BCL6, C2, ICAM1, IL6, C4A, CFB, C4B, MALT1, SERPING1, IDO1, CBLB, TNFSF13B, VEGFA, C1RL, RIPK2, HLA-DRA | 1.19E-08 | 3.66 |
| GO:0002250~adaptive immune response | 16 | ICAM1, NBN, C4A, C3, C4B, CLU, SERPING1, C1R, TLR4, C1S, CD74, IL18BP, VEGFA, C1RL, C2, HLA-DRA | 1.76E-08 | 6.46 |
| GO:0002460~adaptive immune response based on somatic recombination of immune receptors built from immunoglobulin superfamily domains | 16 | ICAM1, NBN, C4A, C3, C4B, CLU, SERPING1, C1R, TLR4, C1S, CD74, IL18BP, VEGFA, C1RL, C2, HLA-DRA | 1.76E-08 | 6.46 |
| GO:0050778~positive regulation of immune response | 21 | C4A, CFB, C3, IL6ST, C4B, CLU, TLR3, MALT1, SERPING1, C1R, TLR4, IDO1, C1S, B2M, TGFB2, TNFSF13B, TAP2, C1RL, RIPK2, C2, HLA-DRA | 3.76E-08 | 4.50 |
| GO:0006959~humoral immune response | 15 | PSMB10, IL6, CCL2, BST2, C4A, C3, C4B, CFB, CLU, SERPING1, C1R, C1S, BCL2, C1RL, C2 | 1.84E-07 | 5.90 |
| GO:0002449~lymphocyte mediated immunity | 14 | ICAM1, NBN, C4A, C4B, C3, CEBPG, CLU, C1RL, C1R, SERPING1, C1S, C2, CD74, HLA-DRA | 2.86E-07 | 6.22 |
| GO:0002526~acute inflammatory response | 16 | IL6, CEBPB, C4A, C3, C4B, CFB, CLU, SERPING1, C1R, TLR4, IDO1, C1S, APOL2, NUPR1, C1RL, C2 | 4.92E-07 | 5.08 |
| GO:0002443~leukocyte mediated immunity | 15 | ICAM1, NBN, IL6, C4A, C4B, C3, CEBPG, CLU, SERPING1, C1R, C1S, CD74, C1RL, C2, HLA-DRA | 5.46E-07 | 5.42 |
| GO:0006954~inflammatory response | 30 | NMI, CCL2, C3, CLU, CXCL9, TLR3, CCL8, C1R, TLR4, C1S, CXCL11, CXCL10, C2, PTX3, CIITA, IL6, CEBPB, C4A, C4B, LY96, CFB, SCUBE1, SERPING1, IDO1, APOL2, APOL3, CCL13, NUPR1, C1RL, RIPK2 | 6.59E-07 | 2.87 |
| GO:0016064~immunoglobulin mediated immune response | 12 | NBN, C4A, C3, C4B, CLU, C1RL, C1R, SERPING1, C1S, C2, CD74, HLA-DRA | 9.00E-07 | 6.91 |
| GO:0019724~B cell mediated immunity | 12 | NBN, C4A, C3, C4B, CLU, C1RL, C1R, SERPING1, C1S, C2, CD74, HLA-DRA | 1.32E-06 | 6.66 |
| GO:0006958~complement activation, classical pathway | 9 | C4A, C3, C4B, CLU, C1RL, C1R, SERPING1, C1S, C2 | 2.50E-06 | 9.65 |
| GO:0002455~humoral immune response mediated by circulating immunoglobulin | 9 | C4A, C3, C4B, CLU, C1RL, C1R, SERPING1, C1S, C2 | 4.33E-06 | 9.03 |
| GO:0006956~complement activation | 10 | C4A, C3, CFB, C4B, CLU, C1RL, C1R, SERPING1, C1S, C2 | 5.76E-06 | 7.40 |
| GO:0002541~activation of plasma proteins involved in acute inflammatory response | 10 | C4A, C3, CFB, C4B, CLU, C1RL, C1R, SERPING1, C1S, C2 | 7.08E-06 | 7.23 |
| GO:0002253~activation of immune response | 14 | C4A, C3, C4B, CFB, CLU, TLR3, MALT1, SERPING1, C1R, TLR4, C1S, C1RL, RIPK2, C2 | 9.03E-06 | 4.63 |
| GO:0009611~response to wounding | 37 | CCL2, NMI, C3, CLU, CXCL9, TLR3, CCL8, TLR4, C1R, C1S, CXCL11, MDK, CXCL10, TGFB2, MAP3K1, BCL2, C2, PTX3, FGF2, CIITA, IL6, CEBPB, C4A, LY96, C4B, CFB, SCUBE1, SERPING1, IDO1, APOL2, APOL3, PLSCR1, CCL13, NUPR1, C1RL, RIPK2, JAK2 | 1.78E-05 | 2.17 |
| GO:0051605~protein maturation by peptide bond cleavage | 10 | C4A, C3, CFB, C4B, CLU, C1RL, C1R, SERPING1, C1S, C2 | 0.0017 | 3.62 |
| GO:0016485~protein processing | 10 | C4A, C3, CFB, C4B, CLU, C1RL, C1R, SERPING1, C1S, C2 | 0.0099 | 2.78 |
| GO:0006957~complement activation, alternative pathway | 4 | C4A, C3, CFB, C2 | 0.0112 | 8.29 |
| GO:0051604~protein maturation | 10 | C4A, C3, CFB, C4B, CLU, C1RL, C1R, SERPING1, C1S, C2 | 0.0167 | 2.55 |
| **Annotation Cluster 3 Enrichment Score: 4.97** | | | | |
| GO:0002237~response to molecule of bacterial origin | 16 | IL6, CCL2, PTGS2, LY96, SOCS1, ADH5, MALT1, TIMP4, TLR4, IDO1, STAT1, B2M, GCH1, TAP2, RIPK2, VLDLR | 8.34E-08 | 5.79 |
| GO:0010033~response to organic substance | 45 | ADCY3, CYP1B1, CCL2, PTGS2, IL6ST, ADH5, TLR3, CALCOCO2, TLR4, ASNS, TIMP4, TRIM16, PMAIP1, C1S, GCH1, TGFB2, B2M, EDNRA, EIF4EBP1, GOT1, TAP2, BCL2, DNAJA1, IDH1, TXNIP, CIITA, IRS2, IL6, SP100, LY96, CFB, SOCS1, MALT1, IDO1, STAT1, DDIT3, ID1, CXCL16, ERN1, RIPK2, JAK2, NFE2L2, EIF2AK2, PPP1R15A, VLDLR | 3.03E-05 | 1.94 |
| GO:0032496~response to lipopolysaccharide | 12 | CCL2, PTGS2, LY96, SOCS1, ADH5, RIPK2, TIMP4, TLR4, IDO1, STAT1, VLDLR, GCH1 | 3.24E-05 | 4.85 |
| GO:0009617~response to bacterium | 18 | IL6, CCL2, PTGS2, LY96, SOCS1, ADH5, TLR3, MALT1, TIMP4, TLR4, IDO1, STAT1, B2M, GCH1, TAP2, ERAP1, RIPK2, VLDLR | 1.62E-04 | 2.90 |
| **Annotation Cluster 4 Enrichment Score: 4.45** | | | | |
| GO:0002504~antigen processing and presentation of peptide or polysaccharide antigen via MHC class II | 10 | HLA-DRB3, IFI30, HLA-DPA1, HLA-DPB1, HLA-DMB, HLA-DOA, HLA-DQA2, CD74, HLA-DQA1, HLA-DRA | 6.44E-07 | 9.42 |
| GO:0019884~antigen processing and presentation of exogenous antigen | 7 | PSME1, TAP2, IFI30, HLA-DOA, CD74, HLA-DRA, B2M | 2.54E-06 | 15.55 |
| GO:0002478~antigen processing and presentation of exogenous peptide antigen | 6 | TAP2, IFI30, HLA-DOA, CD74, HLA-DRA, B2M | 1.31E-05 | 16.96 |
| GO:0002495~antigen processing and presentation of peptide antigen via MHC class II | 4 | IFI30, HLA-DOA, CD74, HLA-DRA | 0.0016 | 15.55 |
| GO:0019886~antigen processing and presentation of exogenous peptide antigen via MHC class II | 4 | IFI30, HLA-DOA, CD74, HLA-DRA | 0.0016 | 15.55 |
| **Annotation Cluster 5 Enrichment Score: 3.78** | | | | |
| GO:0043067~regulation of programmed cell death | 50 | HTATIP2, PTGS2, CBX4, TLR4, PMAIP1, TGFB2, NQO1, MX1, FGF2, ALX3, ARHGEF2, AIFM2, IFI16, DDIT3, JMY, DAPK1, TNFRSF10B, TNFSF13B, HIPK2, VEGFA, ERN1, RIPK2, FOXC1, IFIH1, CCL2, CLU, ASNS, RRM2B, CD74, GCH1, SQSTM1, BCL2, MAP3K1, BCL6, HSPA9, TXNIP, CFLAR, IL6, CEBPB, CEBPG, SMAD6, MALT1, STRADB, IDO1, STAT1, TNFSF10, NUPR1, JAK2, IFI6, TP53INP1 | 1.40E-05 | 1.91 |
| GO:0010941~regulation of cell death | 50 | HTATIP2, PTGS2, CBX4, TLR4, PMAIP1, TGFB2, NQO1, MX1, FGF2, ALX3, ARHGEF2, AIFM2, IFI16, DDIT3, JMY, DAPK1, TNFRSF10B, TNFSF13B, HIPK2, VEGFA, ERN1, RIPK2, FOXC1, IFIH1, CCL2, CLU, ASNS, RRM2B, CD74, GCH1, SQSTM1, BCL2, MAP3K1, BCL6, HSPA9, TXNIP, CFLAR, IL6, CEBPB, CEBPG, SMAD6, MALT1, STRADB, IDO1, STAT1, TNFSF10, NUPR1, JAK2, IFI6, TP53INP1 | 1.55E-05 | 1.91 |
| GO:0043065~positive regulation of apoptosis | 32 | HTATIP2, PTGS2, TLR4, RRM2B, PMAIP1, GCH1, TGFB2, SQSTM1, MAP3K1, BCL2, BCL6, MX1, NQO1, TXNIP, CFLAR, ARHGEF2, CEBPB, AIFM2, CEBPG, IFI16, STAT1, DDIT3, JMY, DAPK1, TNFSF10, TNFRSF10B, NUPR1, HIPK2, ERN1, RIPK2, JAK2, TP53INP1 | 2.26E-05 | 2.31 |
| GO:0042981~regulation of apoptosis | 49 | HTATIP2, PTGS2, CBX4, TLR4, PMAIP1, TGFB2, NQO1, MX1, ALX3, ARHGEF2, AIFM2, IFI16, DDIT3, JMY, DAPK1, TNFRSF10B, TNFSF13B, HIPK2, VEGFA, ERN1, RIPK2, FOXC1, IFIH1, CCL2, CLU, ASNS, RRM2B, CD74, GCH1, SQSTM1, BCL2, MAP3K1, BCL6, HSPA9, TXNIP, CFLAR, IL6, CEBPB, CEBPG, SMAD6, MALT1, STRADB, IDO1, STAT1, TNFSF10, NUPR1, JAK2, IFI6, TP53INP1 | 2.29E-05 | 1.90 |
| GO:0043068~positive regulation of programmed cell death | 32 | HTATIP2, PTGS2, TLR4, RRM2B, PMAIP1, GCH1, TGFB2, SQSTM1, MAP3K1, BCL2, BCL6, MX1, NQO1, TXNIP, CFLAR, ARHGEF2, CEBPB, AIFM2, CEBPG, IFI16, STAT1, DDIT3, JMY, DAPK1, TNFSF10, TNFRSF10B, NUPR1, HIPK2, ERN1, RIPK2, JAK2, TP53INP1 | 2.59E-05 | 2.30 |
| GO:0010942~positive regulation of cell death | 32 | HTATIP2, PTGS2, TLR4, RRM2B, PMAIP1, GCH1, TGFB2, SQSTM1, MAP3K1, BCL2, BCL6, MX1, NQO1, TXNIP, CFLAR, ARHGEF2, CEBPB, AIFM2, CEBPG, IFI16, STAT1, DDIT3, JMY, DAPK1, TNFSF10, TNFRSF10B, NUPR1, HIPK2, ERN1, RIPK2, JAK2, TP53INP1 | 2.82E-05 | 2.29 |
| GO:0006916~anti-apoptosis | 17 | CFLAR, HTATIP2, CCL2, CEBPB, CLU, CBX4, MALT1, STRADB, DAPK1, TNFSF13B, SQSTM1, BCL2, VEGFA, RIPK2, FOXC1, IFI6, HSPA9 | 0.0010 | 2.57 |
| GO:0043066~negative regulation of apoptosis | 24 | CFLAR, IL6, HTATIP2, CCL2, CEBPB, SMAD6, CLU, CBX4, MALT1, ASNS, IDO1, STRADB, CD74, DAPK1, TNFSF13B, SQSTM1, BCL2, HIPK2, VEGFA, RIPK2, FOXC1, BCL6, IFI6, HSPA9 | 0.0011 | 2.11 |
| GO:0043069~negative regulation of programmed cell death | 24 | CFLAR, IL6, HTATIP2, CCL2, CEBPB, SMAD6, CLU, CBX4, MALT1, ASNS, IDO1, STRADB, CD74, DAPK1, TNFSF13B, SQSTM1, BCL2, HIPK2, VEGFA, RIPK2, FOXC1, BCL6, IFI6, HSPA9 | 0.0013 | 2.08 |
| GO:0060548~negative regulation of cell death | 24 | CFLAR, IL6, HTATIP2, CCL2, CEBPB, SMAD6, CLU, CBX4, MALT1, ASNS, IDO1, STRADB, CD74, DAPK1, TNFSF13B, SQSTM1, BCL2, HIPK2, VEGFA, RIPK2, FOXC1, BCL6, IFI6, HSPA9 | 0.0014 | 2.07 |
| GO:0006917~induction of apoptosis | 22 | CFLAR, ARHGEF2, HTATIP2, CEBPB, AIFM2, CEBPG, RRM2B, IFI16, PMAIP1, STAT1, GCH1, JMY, DAPK1, TNFSF10, TNFRSF10B, NUPR1, SQSTM1, HIPK2, ERN1, JAK2, MX1, TP53INP1 | 0.0016 | 2.14 |
| GO:0012502~induction of programmed cell death | 22 | CFLAR, ARHGEF2, HTATIP2, CEBPB, AIFM2, CEBPG, RRM2B, IFI16, PMAIP1, STAT1, GCH1, JMY, DAPK1, TNFSF10, TNFRSF10B, NUPR1, SQSTM1, HIPK2, ERN1, JAK2, MX1, TP53INP1 | 0.0016 | 2.13 |
| **Annotation Cluster 6 Enrichment Score: 3.73** | | | | |
| GO:0043039~tRNA aminoacylation | 11 | IARS, WARS, YARS, CARS, NARS, ZNFX1, SARS, LARS, GARS, EPRS, MARS | 1.51E-06 | 7.44 |
| GO:0043038~amino acid activation | 11 | IARS, WARS, YARS, CARS, NARS, ZNFX1, SARS, LARS, GARS, EPRS, MARS | 1.51E-06 | 7.44 |
| GO:0006418~tRNA aminoacylation for protein translation | 11 | IARS, WARS, YARS, CARS, NARS, ZNFX1, SARS, LARS, GARS, EPRS, MARS | 1.51E-06 | 7.44 |
| GO:0006399~tRNA metabolic process | 11 | IARS, WARS, YARS, CARS, NARS, ZNFX1, SARS, LARS, GARS, EPRS, MARS | 0.0046 | 2.90 |
| GO:0006412~translation | 18 | YARS, CARS, NARS, ZNFX1, SARS, GARS, EPRS, RPL37, EIF1B, RPLP0P2, MRRF, IARS, WARS, RPL23AP32, EIF2S2, LARS, EIF2AK2, MARS | 0.0387 | 1.69 |
| **Annotation Cluster 7 Enrichment Score: 3.62** | | | | |
| GO:0043388~positive regulation of DNA binding | 13 | ICAM1, IL6, SP100, NCOA3, CEBPG, HIPK2, TLR3, RIPK2, EDA2R, MALT1, JAK2, TLR4, JMY | 2.01E-06 | 5.78 |
| GO:0051091~positive regulation of transcription factor activity | 12 | ICAM1, IL6, SP100, NCOA3, CEBPG, TLR3, RIPK2, EDA2R, MALT1, JAK2, TLR4, JMY | 2.71E-06 | 6.22 |
| GO:0051090~regulation of transcription factor activity | 15 | ICAM1, IL6, SP100, CEBPG, EDA2R, TLR3, MALT1, EGLN1, TLR4, DDIT3, JMY, NCOA3, ID1, RIPK2, JAK2 | 5.04E-06 | 4.53 |
| GO:0051099~positive regulation of binding | 13 | ICAM1, IL6, SP100, NCOA3, CEBPG, HIPK2, TLR3, RIPK2, EDA2R, MALT1, JAK2, TLR4, JMY | 6.51E-06 | 5.18 |
| GO:0051101~regulation of DNA binding | 16 | ICAM1, IL6, SP100, CEBPG, EDA2R, TLR3, MALT1, EGLN1, TLR4, DDIT3, JMY, NCOA3, ID1, HIPK2, RIPK2, JAK2 | 7.41E-06 | 4.11 |
| GO:0051098~regulation of binding | 17 | ICAM1, IL6, SP100, CEBPG, TLR3, EDA2R, MALT1, EGLN1, TLR4, DDIT3, JMY, NCOA3, ID1, BCL2, HIPK2, RIPK2, JAK2 | 3.25E-05 | 3.46 |
| GO:0051092~positive regulation of NF-kappaB transcription factor activity | 7 | ICAM1, IL6, TLR3, RIPK2, EDA2R, MALT1, TLR4 | 0.0018 | 5.31 |
| GO:0001819~positive regulation of cytokine production | 10 | DDX58, IL6, IL6ST, TLR3, RIPK2, MALT1, JAK2, TRIM16, TLR4, IDO1 | 0.0024 | 3.46 |
| GO:0043410~positive regulation of MAPKKK cascade | 7 | IL6, HIPK2, TLR3, RIPK2, EDA2R, TLR4, TGFB2 | 0.0041 | 4.54 |
| GO:0043392~negative regulation of DNA binding | 6 | SP100, ID1, CEBPG, JAK2, EGLN1, DDIT3 | 0.0233 | 3.66 |
| GO:0051100~negative regulation of binding | 6 | SP100, ID1, CEBPG, JAK2, EGLN1, DDIT3 | 0.0404 | 3.16 |
| **Annotation Cluster 8 Enrichment Score: 3.49** | | | | |
| GO:0010740~positive regulation of protein kinase cascade | 18 | CFLAR, SECTM1, IL6, BST2, IL6ST, TLR3, EDA2R, MALT1, TLR4, LGALS9, TGFB2, TRIM38, APOL3, TNFSF10, TNFRSF10B, HIPK2, RIPK2, JAK2 | 2.63E-05 | 3.35 |
| GO:0010627~regulation of protein kinase cascade | 21 | SECTM1, CFLAR, IL6, BST2, IL6ST, SOCS1, TLR3, EDA2R, MALT1, TLR4, LGALS9, TGFB2, TRIM38, APOL3, TNFSF10, TNFRSF10B, SQSTM1, MAP3K1, HIPK2, RIPK2, JAK2 | 1.57E-04 | 2.62 |
| GO:0010647~positive regulation of cell communication | 25 | CCL2, PTGS2, IL6ST, CSF1, TLR3, TRIM16, TLR4, JAG1, TGFB2, CFLAR, SECTM1, IL6, BST2, EDA2R, MALT1, LGALS9, TRIM38, APOL3, TNFSF10, TNFRSF10B, NCOA3, VEGFA, HIPK2, RIPK2, JAK2 | 1.60E-04 | 2.36 |
| GO:0043122~regulation of I-kappaB kinase/NF-kappaB cascade | 13 | CFLAR, SECTM1, BST2, TLR3, TLR4, MALT1, LGALS9, TRIM38, APOL3, TNFSF10, TNFRSF10B, SQSTM1, RIPK2 | 1.61E-04 | 3.78 |
| GO:0009967~positive regulation of signal transduction | 23 | SECTM1, CFLAR, IL6, BST2, IL6ST, CSF1, TLR3, EDA2R, MALT1, TRIM16, TLR4, JAG1, LGALS9, TGFB2, TRIM38, APOL3, TNFSF10, TNFRSF10B, NCOA3, HIPK2, VEGFA, RIPK2, JAK2 | 2.18E-04 | 2.42 |
| GO:0043123~positive regulation of I-kappaB kinase/NF-kappaB cascade | 12 | TRIM38, APOL3, SECTM1, CFLAR, TNFSF10, TNFRSF10B, BST2, TLR3, RIPK2, MALT1, TLR4, LGALS9 | 2.71E-04 | 3.85 |
| **Annotation Cluster 9 Enrichment Score: 3.27** | | | | |
| GO:0008219~cell death | 41 | HTATIP2, CLU, TRIB3, PMAIP1, TGFB2, RRAGC, UNC5B, TRIM69, SQSTM1, MAP3K1, BCL2, XAF1, FGF2, RNF144B, CFLAR, ARHGEF2, YARS, IL6, AIFM2, KLF11, RYBP, GARS, OPTN, STAT1, ITPR1, DDIT4, DAPK1, TNFSF10, APOL1, TNFRSF10B, HIPK2, CYFIP2, ERN1, RIPK2, JAK2, EIF2AK2, DRAM1, PPP1R15A, GADD45A, IFI6, TP53INP1 | 4.86E-04 | 1.77 |
| GO:0006915~apoptosis | 36 | HTATIP2, CLU, TRIB3, PMAIP1, RRAGC, UNC5B, TRIM69, SQSTM1, MAP3K1, BCL2, XAF1, FGF2, RNF144B, CFLAR, ARHGEF2, YARS, IL6, AIFM2, KLF11, RYBP, STAT1, DDIT4, DAPK1, TNFSF10, TNFRSF10B, HIPK2, CYFIP2, ERN1, RIPK2, JAK2, EIF2AK2, DRAM1, PPP1R15A, GADD45A, IFI6, TP53INP1 | 5.02E-04 | 1.86 |
| GO:0016265~death | 41 | HTATIP2, CLU, TRIB3, PMAIP1, TGFB2, RRAGC, UNC5B, TRIM69, SQSTM1, MAP3K1, BCL2, XAF1, FGF2, RNF144B, CFLAR, ARHGEF2, YARS, IL6, AIFM2, KLF11, RYBP, GARS, OPTN, STAT1, ITPR1, DDIT4, DAPK1, TNFSF10, APOL1, TNFRSF10B, HIPK2, CYFIP2, ERN1, RIPK2, JAK2, EIF2AK2, DRAM1, PPP1R15A, GADD45A, IFI6, TP53INP1 | 5.45E-04 | 1.76 |
| GO:0012501~programmed cell death | 36 | HTATIP2, CLU, TRIB3, PMAIP1, RRAGC, UNC5B, TRIM69, SQSTM1, MAP3K1, BCL2, XAF1, FGF2, RNF144B, CFLAR, ARHGEF2, YARS, IL6, AIFM2, KLF11, RYBP, STAT1, DDIT4, DAPK1, TNFSF10, TNFRSF10B, HIPK2, CYFIP2, ERN1, RIPK2, JAK2, EIF2AK2, DRAM1, PPP1R15A, GADD45A, IFI6, TP53INP1 | 6.53E-04 | 1.83 |
| **Annotation Cluster 10 Enrichment Score: 2.78** | | | | |
| GO:0051270~regulation of cell motion | 19 | ICAM1, PARD3, IL6, IRS2, SP100, PTPRM, IL6ST, CSF1, JAG1, TGFB2, CXCL10, CXCL16, BCL2, MAP3K1, VEGFA, JAK2, BCL6, FGF2, IGFBP5 | 4.98E-05 | 3.06 |
| GO:0051272~positive regulation of cell motion | 13 | ICAM1, IRS2, IL6, IL6ST, CSF1, TGFB2, CXCL10, CXCL16, BCL2, VEGFA, BCL6, JAK2, FGF2 | 6.83E-05 | 4.13 |
| GO:0030335~positive regulation of cell migration | 12 | ICAM1, IRS2, IL6, IL6ST, CXCL16, BCL2, CSF1, VEGFA, JAK2, FGF2, TGFB2, CXCL10 | 1.25E-04 | 4.19 |
| GO:0040017~positive regulation of locomotion | 12 | ICAM1, IRS2, IL6, IL6ST, CXCL16, BCL2, CSF1, VEGFA, JAK2, FGF2, TGFB2, CXCL10 | 2.97E-04 | 3.81 |
| GO:0030334~regulation of cell migration | 16 | ICAM1, IRS2, IL6, PTPRM, IL6ST, CSF1, JAG1, TGFB2, CXCL10, CXCL16, BCL2, MAP3K1, VEGFA, JAK2, FGF2, IGFBP5 | 3.57E-04 | 2.94 |
| GO:0040012~regulation of locomotion | 16 | ICAM1, IRS2, IL6, PTPRM, IL6ST, CSF1, JAG1, TGFB2, CXCL10, CXCL16, BCL2, MAP3K1, VEGFA, JAK2, FGF2, IGFBP5 | 0.0013 | 2.59 |
| **Annotation Cluster 11 Enrichment Score: 2.51** | | | | |
| GO:0051092~positive regulation of NF-kappaB transcription factor activity | 7 | ICAM1, IL6, TLR3, RIPK2, EDA2R, MALT1, TLR4 | 0.0018 | 5.31 |
| GO:0001817~regulation of cytokine production | 15 | IL6, CEBPB, IL6ST, CEBPG, TLR3, MALT1, TLR4, TRIM16, IDO1, TGFB2, DDX58, IRF1, RIPK2, JAK2, BCL6 | 0.0021 | 2.58 |
| GO:0031349~positive regulation of defense response | 9 | EDNRA, IL6, C3, IL6ST, TLR3, RIPK2, JAK2, TLR4, IDO1 | 0.0023 | 3.83 |
| GO:0001819~positive regulation of cytokine production | 10 | DDX58, IL6, IL6ST, TLR3, RIPK2, MALT1, JAK2, TRIM16, TLR4, IDO1 | 0.0024 | 3.46 |
| GO:0050865~regulation of cell activation | 14 | CD47, IL6, CBLB, TNFSF13B, IL6ST, CD274, RIPK2, MALT1, JAK2, TLR4, BCL6, IDO1, HLA-DOA, CD74 | 0.0042 | 2.49 |
| GO:0050867~positive regulation of cell activation | 10 | CD47, IL6, TNFSF13B, IL6ST, RIPK2, MALT1, BCL6, JAK2, TLR4, CD74 | 0.0094 | 2.80 |
| **Annotation Cluster 12 Enrichment Score: 2.38** | | | | |
| GO:0009069~serine family amino acid metabolic process | 7 | CTH, SEPHS2, SHMT2, PHGDH, DMGDH, PSAT1, PSPH | 1.40E-04 | 8.37 |
| GO:0009070~serine family amino acid biosynthetic process | 5 | CTH, SEPHS2, PHGDH, PSAT1, PSPH | 2.88E-04 | 14.14 |
| GO:0009309~amine biosynthetic process | 10 | CTH, SEPHS2, GOT1, ASS1, PHGDH, ASNS, PSAT1, PSPH, GCH1, TGFB2 | 0.0011 | 3.84 |
| GO:0008652~cellular amino acid biosynthetic process | 8 | CTH, SEPHS2, GOT1, ASS1, PHGDH, ASNS, PSAT1, PSPH | 0.0011 | 4.88 |
| GO:0006563~L-serine metabolic process | 4 | SHMT2, PHGDH, PSAT1, PSPH | 0.0016 | 15.55 |
| GO:0006564~L-serine biosynthetic process | 3 | PHGDH, PSAT1, PSPH | 0.0030 | 31.10 |
| GO:0016053~organic acid biosynthetic process | 13 | SEPHS2, CTH, GOT1, PTGS2, PTGDS, ASS1, PHGDH, LIAS, ASNS, IDO1, PSAT1, PSPH, CD74 | 0.0042 | 2.61 |
| GO:0046394~carboxylic acid biosynthetic process | 13 | SEPHS2, CTH, GOT1, PTGS2, PTGDS, ASS1, PHGDH, LIAS, ASNS, IDO1, PSAT1, PSPH, CD74 | 0.0042 | 2.61 |
| GO:0044271~nitrogen compound biosynthetic process | 18 | ADCY3, MOCOS, NAMPT, SEPHS2, PRTFDC1, ASS1, ASNS, RRM2B, ATP6V1B2, PSPH, GCH1, CMPK2, TGFB2, CTH, GOT1, PHGDH, PSAT1, NQO1 | 0.0334 | 1.72 |
| **Annotation Cluster 13 Enrichment Score: 2.00** | | | | |
| GO:0034620~cellular response to unfolded protein | 5 | ERN1, NFE2L2, EIF2AK2, PPP1R15A, DDIT3 | 0.0041 | 7.40 |
| GO:0030968~endoplasmic reticulum unfolded protein response | 5 | ERN1, NFE2L2, EIF2AK2, PPP1R15A, DDIT3 | 0.0041 | 7.40 |
| GO:0034976~response to endoplasmic reticulum stress | 6 | ERN1, NFE2L2, EIF2AK2, FAM129A, PPP1R15A, DDIT3 | 0.0043 | 5.49 |
| GO:0051789~response to protein stimulus | 9 | ID1, CFB, BCL2, DNAJA1, ERN1, NFE2L2, EIF2AK2, PPP1R15A, DDIT3 | 0.0217 | 2.62 |
| GO:0006984~ER-nuclear signaling pathway | 5 | ERN1, NFE2L2, EIF2AK2, PPP1R15A, DDIT3 | 0.0249 | 4.44 |
| GO:0006986~response to unfolded protein | 7 | CFB, DNAJA1, ERN1, NFE2L2, EIF2AK2, PPP1R15A, DDIT3 | 0.0261 | 3.07 |
| **Annotation Cluster 14 Enrichment Score: 1.99** | | | | |
| GO:0008637~apoptotic mitochondrial changes | 6 | AIFM2, MAP3K1, BCL2, CLU, PMAIP1, IFI6 | 0.0028 | 6.02 |
| GO:0007005~mitochondrion organization | 11 | GRPEL2, LONP1, AIFM2, EPAS1, MTX3, MAP3K1, BCL2, CLU, RRM2B, PMAIP1, IFI6 | 0.0134 | 2.48 |
| GO:0001836~release of cytochrome c from mitochondria | 4 | BCL2, CLU, PMAIP1, IFI6 | 0.0284 | 5.92 |
| **Annotation Cluster 15 Enrichment Score: 1.94** | | | | |
| GO:0044093~positive regulation of molecular function | 35 | PSMB10, ADCY3, PARD3, CSF1, TLR3, KITLG, TLR4, PMAIP1, CD74, GCH1, TGFB2, EDNRA, MAP3K1, BCL2, FGF2, ICAM1, IL6, SP100, CEBPG, EDA2R, MALT1, STRADB, STAT1, JMY, PSMB9, TNFRSF10B, PSME1, NCOA3, PSME2, PSMA4, HIPK2, ERN1, RIPK2, JAK2, VLDLR | 6.22E-04 | 1.86 |
| GO:0032147~activation of protein kinase activity | 11 | EDNRA, ADCY3, PARD3, TNFRSF10B, MAP3K1, TLR3, MALT1, JAK2, STRADB, FGF2, TGFB2 | 0.0036 | 3.00 |
| GO:0045860~positive regulation of protein kinase activity | 16 | ADCY3, PARD3, CSF1, TLR3, KITLG, MALT1, STRADB, CD74, TGFB2, EDNRA, TNFRSF10B, MAP3K1, ERN1, JAK2, FGF2, VLDLR | 0.0056 | 2.23 |
| GO:0033674~positive regulation of kinase activity | 16 | ADCY3, PARD3, CSF1, TLR3, KITLG, MALT1, STRADB, CD74, TGFB2, EDNRA, TNFRSF10B, MAP3K1, ERN1, JAK2, FGF2, VLDLR | 0.0077 | 2.15 |
| GO:0042325~regulation of phosphorylation | 26 | ADCY3, PARD3, IL6ST, CSF1, TLR3, TRIB3, KITLG, TLR4, CD74, TGFB2, EDNRA, BCL2, MAP3K1, FAM129A, FGF2, IL6, SMAD6, SOCS1, MALT1, STRADB, TNFRSF10B, ERN1, JAK2, GADD45A, VLDLR, DUSP6 | 0.0085 | 1.74 |
| GO:0051347~positive regulation of transferase activity | 16 | ADCY3, PARD3, CSF1, TLR3, KITLG, MALT1, STRADB, CD74, TGFB2, EDNRA, TNFRSF10B, MAP3K1, ERN1, JAK2, FGF2, VLDLR | 0.0107 | 2.07 |
| GO:0019220~regulation of phosphate metabolic process | 26 | ADCY3, PARD3, IL6ST, CSF1, TLR3, TRIB3, KITLG, TLR4, CD74, TGFB2, EDNRA, BCL2, MAP3K1, FAM129A, FGF2, IL6, SMAD6, SOCS1, MALT1, STRADB, TNFRSF10B, ERN1, JAK2, GADD45A, VLDLR, DUSP6 | 0.0138 | 1.67 |
| GO:0051174~regulation of phosphorus metabolic process | 26 | ADCY3, PARD3, IL6ST, CSF1, TLR3, TRIB3, KITLG, TLR4, CD74, TGFB2, EDNRA, BCL2, MAP3K1, FAM129A, FGF2, IL6, SMAD6, SOCS1, MALT1, STRADB, TNFRSF10B, ERN1, JAK2, GADD45A, VLDLR, DUSP6 | 0.0138 | 1.67 |
| GO:0045859~regulation of protein kinase activity | 19 | ADCY3, PARD3, CSF1, TLR3, TRIB3, KITLG, MALT1, STRADB, CD74, TGFB2, EDNRA, TNFRSF10B, MAP3K1, ERN1, JAK2, FGF2, GADD45A, VLDLR, DUSP6 | 0.0299 | 1.71 |
| GO:0043549~regulation of kinase activity | 19 | ADCY3, PARD3, CSF1, TLR3, TRIB3, KITLG, MALT1, STRADB, CD74, TGFB2, EDNRA, TNFRSF10B, MAP3K1, ERN1, JAK2, FGF2, GADD45A, VLDLR, DUSP6 | 0.0398 | 1.66 |
| GO:0043085~positive regulation of catalytic activity | 25 | PSMB10, ADCY3, PARD3, CSF1, TLR3, KITLG, MALT1, PMAIP1, STRADB, STAT1, CD74, GCH1, TGFB2, PSMB9, EDNRA, TNFRSF10B, PSME1, PSME2, BCL2, PSMA4, MAP3K1, ERN1, JAK2, FGF2, VLDLR | 0.0485 | 1.50 |
| **Annotation Cluster 16 Enrichment Score: 1.91** | | | | |
| GO:0048872~homeostasis of number of cells | 11 | IL6, TNFSF13B, EPAS1, BCL2, CSF1, CEBPG, VPS54, VEGFA, DYRK3, BCL6, TCEA1 | 0.0014 | 3.42 |
| GO:0030097~hemopoiesis | 17 | EPAS1, CEBPG, CSF1, KITLG, MALT1, IFI16, JAG1, CD74, TGFB2, FLT3LG, BCL2, VEGFA, IRF1, JAK2, TCEA1, BCL6, DYRK3 | 0.0040 | 2.24 |
| GO:0002520~immune system development | 18 | NBN, EPAS1, CEBPG, CSF1, KITLG, MALT1, IFI16, JAG1, CD74, TGFB2, FLT3LG, BCL2, VEGFA, IRF1, JAK2, TCEA1, BCL6, DYRK3 | 0.0079 | 2.03 |
| GO:0048534~hemopoietic or lymphoid organ development | 17 | EPAS1, CEBPG, CSF1, KITLG, MALT1, IFI16, JAG1, CD74, TGFB2, FLT3LG, BCL2, VEGFA, IRF1, JAK2, TCEA1, BCL6, DYRK3 | 0.0099 | 2.03 |
| GO:0030099~myeloid cell differentiation | 9 | EPAS1, CSF1, CEBPG, VEGFA, DYRK3, BCL6, JAK2, TCEA1, IFI16 | 0.0099 | 3.01 |
| GO:0030218~erythrocyte differentiation | 6 | EPAS1, CEBPG, VEGFA, DYRK3, BCL6, TCEA1 | 0.0117 | 4.34 |
| GO:0034101~erythrocyte homeostasis | 6 | EPAS1, CEBPG, VEGFA, DYRK3, BCL6, TCEA1 | 0.0199 | 3.81 |
| **Annotation Cluster 17 Enrichment Score: 1.82** | | | | |
| GO:0043388~positive regulation of DNA binding | 13 | ICAM1, IL6, SP100, NCOA3, CEBPG, HIPK2, TLR3, RIPK2, EDA2R, MALT1, JAK2, TLR4, JMY | 2.01E-06 | 5.78 |
| GO:0051091~positive regulation of transcription factor activity | 12 | ICAM1, IL6, SP100, NCOA3, CEBPG, TLR3, RIPK2, EDA2R, MALT1, JAK2, TLR4, JMY | 2.71E-06 | 6.22 |
| GO:0051099~positive regulation of binding | 13 | ICAM1, IL6, SP100, NCOA3, CEBPG, HIPK2, TLR3, RIPK2, EDA2R, MALT1, JAK2, TLR4, JMY | 6.51E-06 | 5.18 |
| GO:0070304~positive regulation of stress-activated protein kinase signaling pathway | 6 | HIPK2, TLR3, RIPK2, EDA2R, TLR4, TGFB2 | 4.42E-04 | 8.89 |
| GO:0046330~positive regulation of JNK cascade | 5 | HIPK2, TLR3, RIPK2, EDA2R, TLR4 | 0.0018 | 9.15 |
| GO:0051092~positive regulation of NF-kappaB transcription factor activity | 7 | ICAM1, IL6, TLR3, RIPK2, EDA2R, MALT1, TLR4 | 0.0018 | 5.31 |
| GO:0031349~positive regulation of defense response | 9 | EDNRA, IL6, C3, IL6ST, TLR3, RIPK2, JAK2, TLR4, IDO1 | 0.0023 | 3.83 |
| GO:0043410~positive regulation of MAPKKK cascade | 7 | IL6, HIPK2, TLR3, RIPK2, EDA2R, TLR4, TGFB2 | 0.0041 | 4.54 |
| GO:0032755~positive regulation of interleukin-6 production | 4 | IL6, TLR3, RIPK2, TLR4 | 0.0217 | 6.55 |
| GO:0070302~regulation of stress-activated protein kinase signaling pathway | 7 | MAP3K1, HIPK2, TLR3, RIPK2, EDA2R, TLR4, TGFB2 | 0.0231 | 3.15 |
| GO:0032675~regulation of interleukin-6 production | 5 | IL6, CEBPB, TLR3, RIPK2, TLR4 | 0.0274 | 4.32 |
| GO:0045088~regulation of innate immune response | 6 | SAMHD1, ERAP1, TLR3, RIPK2, SERPING1, TLR4 | 0.0290 | 3.46 |
| GO:0032874~positive regulation of stress-activated MAPK cascade | 3 | RIPK2, TLR4, TGFB2 | 0.0318 | 10.37 |
| GO:0032872~regulation of stress-activated MAPK cascade | 3 | RIPK2, TLR4, TGFB2 | 0.0390 | 9.33 |
| GO:0080135~regulation of cellular response to stress | 8 | MAP3K1, CEBPG, HIPK2, TLR3, RIPK2, EDA2R, TLR4, TGFB2 | 0.0477 | 2.42 |
| **Annotation Cluster 18 Enrichment Score: 1.77** | | | | |
| GO:0007259~JAK-STAT cascade | 8 | NMI, CCL2, IL6ST, SOCS1, PKD2, JAK2, STAT1, STAT2 | 2.11E-04 | 6.38 |
| GO:0050729~positive regulation of inflammatory response | 7 | EDNRA, IL6, C3, IL6ST, JAK2, TLR4, IDO1 | 3.25E-04 | 7.26 |
| GO:0050727~regulation of inflammatory response | 10 | EDNRA, IL6, PTGS2, C3, IL6ST, BCL6, SERPING1, JAK2, TLR4, IDO1 | 7.03E-04 | 4.09 |
| GO:0031349~positive regulation of defense response | 9 | EDNRA, IL6, C3, IL6ST, TLR3, RIPK2, JAK2, TLR4, IDO1 | 0.0023 | 3.83 |
| GO:0032103~positive regulation of response to external stimulus | 8 | EDNRA, IL6, C3, IL6ST, VEGFA, JAK2, TLR4, IDO1 | 0.0043 | 3.89 |
| GO:0051247~positive regulation of protein metabolic process | 17 | PSMB10, IL6, IL6ST, CSF1, KITLG, TLR4, PSMB9, EDNRA, CBLB, PSME1, PSME2, BCL2, PSMA4, MDM2, JAK2, FAM129A, FGF2 | 0.0053 | 2.18 |
| GO:0031401~positive regulation of protein modification process | 14 | PSMB10, IL6, IL6ST, KITLG, TLR4, PSMB9, EDNRA, PSME1, PSME2, PSMA4, BCL2, JAK2, FAM129A, FGF2 | 0.0073 | 2.33 |
| GO:0001934~positive regulation of protein amino acid phosphorylation | 9 | EDNRA, IL6, IL6ST, BCL2, KITLG, JAK2, TLR4, FAM129A, FGF2 | 0.0077 | 3.14 |
| GO:0032270~positive regulation of cellular protein metabolic process | 16 | PSMB10, IL6, IL6ST, CSF1, KITLG, TLR4, PSMB9, EDNRA, PSME1, PSME2, BCL2, PSMA4, MDM2, JAK2, FAM129A, FGF2 | 0.0084 | 2.14 |
| GO:0042325~regulation of phosphorylation | 26 | ADCY3, PARD3, IL6ST, CSF1, TLR3, TRIB3, KITLG, TLR4, CD74, TGFB2, EDNRA, BCL2, MAP3K1, FAM129A, FGF2, IL6, SMAD6, SOCS1, MALT1, STRADB, TNFRSF10B, ERN1, JAK2, GADD45A, VLDLR, DUSP6 | 0.0085 | 1.74 |
| GO:0042327~positive regulation of phosphorylation | 9 | EDNRA, IL6, IL6ST, BCL2, KITLG, JAK2, TLR4, FAM129A, FGF2 | 0.0126 | 2.89 |
| GO:0019220~regulation of phosphate metabolic process | 26 | ADCY3, PARD3, IL6ST, CSF1, TLR3, TRIB3, KITLG, TLR4, CD74, TGFB2, EDNRA, BCL2, MAP3K1, FAM129A, FGF2, IL6, SMAD6, SOCS1, MALT1, STRADB, TNFRSF10B, ERN1, JAK2, GADD45A, VLDLR, DUSP6 | 0.0138 | 1.67 |
| GO:0051174~regulation of phosphorus metabolic process | 26 | ADCY3, PARD3, IL6ST, CSF1, TLR3, TRIB3, KITLG, TLR4, CD74, TGFB2, EDNRA, BCL2, MAP3K1, FAM129A, FGF2, IL6, SMAD6, SOCS1, MALT1, STRADB, TNFRSF10B, ERN1, JAK2, GADD45A, VLDLR, DUSP6 | 0.0138 | 1.67 |
| GO:0045937~positive regulation of phosphate metabolic process | 9 | EDNRA, IL6, IL6ST, BCL2, KITLG, JAK2, TLR4, FAM129A, FGF2 | 0.0150 | 2.80 |
| GO:0010562~positive regulation of phosphorus metabolic process | 9 | EDNRA, IL6, IL6ST, BCL2, KITLG, JAK2, TLR4, FAM129A, FGF2 | 0.0150 | 2.80 |
| GO:0042516~regulation of tyrosine phosphorylation of Stat3 protein | 4 | IL6, IL6ST, SOCS1, JAK2 | 0.0160 | 7.32 |
| GO:0032268~regulation of cellular protein metabolic process | 25 | PSMB10, IL6ST, CSF1, MKNK2, KITLG, TLR4, EDNRA, EIF4EBP1, BCL2, MAP3K1, FAM129A, FGF2, IL6, SMAD6, SOCS1, SERPING1, EIF1B, PSMB9, PSME1, PSME2, PSMA4, MDM2, JAK2, PPP1R15A, IGFBP5 | 0.0189 | 1.64 |
| GO:0001932~regulation of protein amino acid phosphorylation | 12 | EDNRA, IL6, IL6ST, MAP3K1, BCL2, SMAD6, SOCS1, KITLG, JAK2, TLR4, FAM129A, FGF2 | 0.0238 | 2.16 |
| GO:0002673~regulation of acute inflammatory response | 4 | IL6, C3, IL6ST, SERPING1 | 0.0284 | 5.92 |
| GO:0031399~regulation of protein modification process | 17 | PSMB10, IL6, IL6ST, SMAD6, SOCS1, KITLG, TLR4, PSMB9, EDNRA, PSME1, PSME2, BCL2, PSMA4, MAP3K1, JAK2, FAM129A, FGF2 | 0.0288 | 1.79 |
| GO:0032101~regulation of response to external stimulus | 11 | EDNRA, IL6, PTGS2, C3, IL6ST, VEGFA, BCL6, SERPING1, JAK2, TLR4, IDO1 | 0.0323 | 2.15 |
| GO:0042517~positive regulation of tyrosine phosphorylation of Stat3 protein | 3 | IL6, IL6ST, JAK2 | 0.0466 | 8.48 |
| **Annotation Cluster 19 Enrichment Score: 1.74** | | | | |
| GO:0007179~transforming growth factor beta receptor signaling pathway | 7 | FMOD, CCL2, ID1, MAP3K1, SMAD6, GDF15, TGFB2 | 0.0122 | 3.63 |
| GO:0007178~transmembrane receptor protein serine/threonine kinase signaling pathway | 9 | FMOD, CCL2, ID1, MAP3K1, SMAD6, HIPK2, FST, GDF15, TGFB2 | 0.0177 | 2.72 |
| GO:0007167~enzyme linked receptor protein signaling pathway | 19 | TXNIP, FMOD, IRS2, CCL2, IL6ST, SMAD6, ARID5B, FST, TGFB2, EPS15, EIF4EBP1, ID1, MAP3K1, HIPK2, VEGFA, JAK2, FOXC1, GDF15, FGF2 | 0.0277 | 1.73 |
| **Annotation Cluster 20 Enrichment Score: 1.66** | | | | |
| GO:0001666~response to hypoxia | 12 | EDNRA, LONP1, CCL2, EPAS1, BCL2, VEGFA, CAMK2D, EGLN1, ITPR1, VLDLR, DDIT4, TGFB2 | 0.0039 | 2.78 |
| GO:0070482~response to oxygen levels | 12 | EDNRA, LONP1, CCL2, EPAS1, BCL2, VEGFA, CAMK2D, EGLN1, ITPR1, VLDLR, DDIT4, TGFB2 | 0.0057 | 2.65 |
| **Annotation Cluster 21 Enrichment Score: 1.61** | | | | |
| GO:0002821~positive regulation of adaptive immune response | 7 | TNFSF13B, C3, IL6ST, TAP2, MALT1, IDO1, B2M | 3.92E-04 | 7.02 |
| GO:0002819~regulation of adaptive immune response | 8 | TNFSF13B, C3, IL6ST, TAP2, MALT1, BCL6, IDO1, B2M | 0.0020 | 4.44 |
| GO:0002824~positive regulation of adaptive immune response based on somatic recombination of immune receptors built from immunoglobulin superfamily domains | 6 | TNFSF13B, C3, TAP2, MALT1, IDO1, B2M | 0.0024 | 6.22 |
| GO:0002822~regulation of adaptive immune response based on somatic recombination of immune receptors built from immunoglobulin superfamily domains | 7 | TNFSF13B, C3, TAP2, MALT1, BCL6, IDO1, B2M | 0.0081 | 3.96 |
| GO:0002697~regulation of immune effector process | 9 | DDX58, ICAM1, IL6, C3, TAP2, MALT1, BCL6, SERPING1, B2M | 0.0158 | 2.77 |

Table S4. Functional annotation clustering-Gene Ontology Terms for Biological Processes: MSC-γ vs. UT-MSC (downregulated genes)

| **Term** | **Count** | **Genes** | **P-Value** | **Fold Enrichment** |
| --- | --- | --- | --- | --- |
| **Annotation Cluster 1 Enrichment Score: 11.10** | | | |  |
| GO:0030198~extracellular matrix organization | 17 | RECK, LUM, COL3A1, ELN, CCDC80, DCN, COL5A2, COL5A1, EMILIN1, COL14A1, P4HA1, TGFBI, COL1A2, COL12A1, COL1A1, COL11A1, ADAMTS2 | 4.73E-13 | 12.22 |
| GO:0030199~collagen fibril organization | 11 | COL14A1, P4HA1, LUM, COL3A1, COL1A2, COL12A1, COL1A1, COL5A2, COL11A1, ADAMTS2, COL5A1 | 2.18E-12 | 28.35 |
| GO:0043062~extracellular structure organization | 17 | RECK, LUM, COL3A1, ELN, CCDC80, DCN, COL5A2, COL5A1, EMILIN1, COL14A1, P4HA1, TGFBI, COL1A2, COL12A1, COL1A1, COL11A1, ADAMTS2 | 5.00E-10 | 7.80 |
| **Annotation Cluster 2 Enrichment Score: 8.80** | | | |  |
| GO:0000279~M phase | 25 | KIFC1, PRC1, TUBB2A, ANLN, AURKB, SPC24, CDCA8, NCAPH, HSPA2, CCNA2, TRIP13, CDC6, KIF11, MKI67, DLGAP5, CCNF, TPX2, BIRC5, CDC20, PBK, UBE2C, CDK2, RAD51, CCNB1, PLK1 | 1.11E-11 | 5.68 |
| GO:0000280~nuclear division | 20 | CDC6, KIFC1, KIF11, TUBB2A, DLGAP5, CCNF, TPX2, CDC20, ANLN, BIRC5, AURKB, PBK, UBE2C, CDK2, SPC24, CCNB1, NCAPH, CDCA8, PLK1, CCNA2 | 1.02E-10 | 6.79 |
| GO:0007067~mitosis | 20 | CDC6, KIFC1, KIF11, TUBB2A, DLGAP5, CCNF, TPX2, CDC20, ANLN, BIRC5, AURKB, PBK, UBE2C, CDK2, SPC24, CCNB1, NCAPH, CDCA8, PLK1, CCNA2 | 1.02E-10 | 6.79 |
| GO:0000087~M phase of mitotic cell cycle | 20 | CDC6, KIFC1, KIF11, TUBB2A, DLGAP5, CCNF, TPX2, CDC20, ANLN, BIRC5, AURKB, PBK, UBE2C, CDK2, SPC24, CCNB1, NCAPH, CDCA8, PLK1, CCNA2 | 1.40E-10 | 6.67 |
| GO:0048285~organelle fission | 20 | CDC6, KIFC1, KIF11, TUBB2A, DLGAP5, CCNF, TPX2, CDC20, ANLN, BIRC5, AURKB, PBK, UBE2C, CDK2, SPC24, CCNB1, NCAPH, CDCA8, PLK1, CCNA2 | 2.04E-10 | 6.53 |
| GO:0022403~cell cycle phase | 26 | KIFC1, PRC1, TUBB2A, ANLN, AURKB, GTSE1, SPC24, NCAPH, CDCA8, HSPA2, CCNA2, TRIP13, CDC6, KIF11, MKI67, DLGAP5, CCNF, TPX2, BIRC5, CDC20, PBK, UBE2C, CDK2, RAD51, CCNB1, PLK1 | 2.35E-10 | 4.69 |
| GO:0022402~cell cycle process | 28 | KIFC1, PRC1, TUBB2A, ANLN, AURKB, CALR, GTSE1, SPC24, CDCA8, NCAPH, HSPA2, CCNA2, TRIP13, DHCR24, CDC6, KIF11, MKI67, DLGAP5, CCNF, TPX2, BIRC5, CDC20, PBK, UBE2C, CDK2, RAD51, CCNB1, PLK1 | 7.19E-09 | 3.70 |
| GO:0000278~mitotic cell cycle | 22 | CDC6, KIFC1, KIF11, PRC1, TUBB2A, DLGAP5, CCNF, TPX2, CDC20, ANLN, BIRC5, AURKB, PBK, UBE2C, CDK2, GTSE1, SPC24, CCNB1, NCAPH, CDCA8, PLK1, CCNA2 | 2.14E-08 | 4.44 |
| GO:0007049~cell cycle | 30 | KIFC1, PRC1, TUBB2A, FOXM1, ANLN, AURKB, CALR, GTSE1, SPC24, CDCA8, NCAPH, HSPA2, HJURP, CCNA2, DHCR24, TRIP13, CDC6, KIF11, MKI67, DLGAP5, CCNF, TPX2, BIRC5, CDC20, PBK, UBE2C, CDK2, RAD51, CCNB1, PLK1 | 3.98E-07 | 2.89 |
| GO:0051301~cell division | 17 | KIFC1, CDC6, KIF11, PRC1, CCNF, CDC20, ANLN, BIRC5, AURKB, UBE2C, CDK2, SPC24, CCNB1, NCAPH, CDCA8, PLK1, CCNA2 | 2.04E-06 | 4.31 |
| **Annotation Cluster 3 Enrichment Score: 4.20** | | | |  |
| GO:0030199~collagen fibril organization | 11 | COL14A1, P4HA1, LUM, COL3A1, COL1A2, COL12A1, COL1A1, COL5A2, COL11A1, ADAMTS2, COL5A1 | 2.18E-12 | 28.35 |
| GO:0043588~skin development | 7 | COL3A1, COL1A2, COL1A1, COL5A2, ADAMTS2, COL5A1, DHCR24 | 1.88E-06 | 18.04 |
| GO:0008544~epidermis development | 11 | FLG, CRABP2, KRT14, COL3A1, COL1A2, COL1A1, COL5A2, SNAI1, ADAMTS2, COL5A1, DHCR24 | 1.75E-04 | 4.47 |
| GO:0007398~ectoderm development | 11 | FLG, CRABP2, KRT14, COL3A1, COL1A2, COL1A1, COL5A2, SNAI1, ADAMTS2, COL5A1, DHCR24 | 3.29E-04 | 4.13 |
| GO:0032964~collagen biosynthetic process | 3 | COL3A1, COL1A1, COL5A1 | 0.001715 | 44.84 |
| GO:0032963~collagen metabolic process | 4 | COL3A1, COL1A1, ADAMTS2, COL5A1 | 0.005943 | 10.68 |
| GO:0044259~multicellular organismal macromolecule metabolic process | 4 | COL3A1, COL1A1, ADAMTS2, COL5A1 | 0.00792 | 9.64 |
| GO:0044236~multicellular organismal metabolic process | 4 | COL3A1, COL1A1, ADAMTS2, COL5A1 | 0.012918 | 8.08 |
| **Annotation Cluster 4 Enrichment Score: 3.46** | | | |  |
| GO:0016126~sterol biosynthetic process | 7 | EBP, MVD, HMGCR, SQLE, DHCR7, HMGCS1, DHCR24 | 6.01E-06 | 14.95 |
| GO:0006695~cholesterol biosynthetic process | 6 | EBP, MVD, HMGCR, DHCR7, HMGCS1, DHCR24 | 2.07E-05 | 17.25 |
| GO:0008203~cholesterol metabolic process | 9 | SREBF1, EBP, LDLR, MVD, HMGCR, SQLE, DHCR7, HMGCS1, DHCR24 | 3.02E-05 | 7.31 |
| GO:0016125~sterol metabolic process | 9 | SREBF1, EBP, LDLR, MVD, HMGCR, SQLE, DHCR7, HMGCS1, DHCR24 | 5.93E-05 | 6.66 |
| GO:0008610~lipid biosynthetic process | 15 | EBP, MVD, HMGCR, SCD, HMGCS1, FADS2, ACLY, LPCAT3, PLAUR, SQLE, ANG, DHCR7, FASN, DHCR24, PC | 1.07E-04 | 3.47 |
| GO:0006694~steroid biosynthetic process | 7 | EBP, MVD, HMGCR, SQLE, DHCR7, HMGCS1, DHCR24 | 9.36E-04 | 6.16 |
| GO:0008202~steroid metabolic process | 9 | SREBF1, EBP, LDLR, MVD, HMGCR, SQLE, DHCR7, HMGCS1, DHCR24 | 0.005649 | 3.33 |
| GO:0006720~isoprenoid metabolic process | 4 | MVD, HMGCR, CRABP2, HMGCS1 | 0.020584 | 6.79 |
| GO:0008299~isoprenoid biosynthetic process | 3 | MVD, HMGCR, HMGCS1 | 0.028588 | 11.21 |
| **Annotation Cluster 5 Enrichment Score: 2.76** | | | |  |
| GO:0006323~DNA packaging | 10 | HIST1H2BO, HIST2H2AB, NCAPH, HIST1H2BM, HIST1H1C, HJURP, HIST1H1B, HIST1H2AJ, ASF1B, TOP2A | 2.58E-05 | 6.39 |
| GO:0006334~nucleosome assembly | 8 | HIST1H2BO, HIST2H2AB, HIST1H2BM, HIST1H1C, HJURP, HIST1H1B, HIST1H2AJ, ASF1B | 1.26E-04 | 7.12 |
| GO:0031497~chromatin assembly | 8 | HIST1H2BO, HIST2H2AB, HIST1H2BM, HIST1H1C, HJURP, HIST1H1B, HIST1H2AJ, ASF1B | 1.57E-04 | 6.87 |
| GO:0065004~protein-DNA complex assembly | 8 | HIST1H2BO, HIST2H2AB, HIST1H2BM, HIST1H1C, HJURP, HIST1H1B, HIST1H2AJ, ASF1B | 2.08E-04 | 6.57 |
| GO:0034728~nucleosome organization | 8 | HIST1H2BO, HIST2H2AB, HIST1H2BM, HIST1H1C, HJURP, HIST1H1B, HIST1H2AJ, ASF1B | 2.38E-04 | 6.43 |
| GO:0034622~cellular macromolecular complex assembly | 14 | HIST1H2BO, HIST2H2AB, HIST1H2BM, HIST1H1C, ANG, TUBB2A, HJURP, HIST1H1B, ANLN, HIST1H2AJ, CALR, ASF1B, TUBA1A, TUBA1B | 3.34E-04 | 3.29 |
| GO:0034621~cellular macromolecular complex subunit organization | 14 | HIST1H2BO, HIST2H2AB, HIST1H2BM, HIST1H1C, ANG, TUBB2A, HJURP, HIST1H1B, ANLN, HIST1H2AJ, CALR, ASF1B, TUBA1A, TUBA1B | 9.85E-04 | 2.93 |
| GO:0065003~macromolecular complex assembly | 20 | HIST1H1C, HIST1H1B, TUBB2A, ANLN, CALR, SOD2, RAD51, JUP, HIST1H2BO, HIST2H2AB, HIST1H2BM, ANG, HJURP, RRM2, CDA, QPRT, HIST1H2AJ, TUBA1A, ASF1B, TUBA1B | 0.001388 | 2.25 |
| GO:0006333~chromatin assembly or disassembly | 8 | HIST1H2BO, HIST2H2AB, HIST1H2BM, HIST1H1C, HJURP, HIST1H1B, HIST1H2AJ, ASF1B | 0.001537 | 4.71 |
| GO:0043933~macromolecular complex subunit organization | 20 | HIST1H1C, HIST1H1B, TUBB2A, ANLN, CALR, SOD2, RAD51, JUP, HIST1H2BO, HIST2H2AB, HIST1H2BM, ANG, HJURP, RRM2, CDA, QPRT, HIST1H2AJ, TUBA1A, ASF1B, TUBA1B | 0.002907 | 2.11 |
| GO:0051276~chromosome organization | 13 | KIFC1, HIST1H1C, HIST1H1B, DLGAP5, HIST1H2BO, HIST2H2AB, CDCA8, HIST1H2BM, NCAPH, HJURP, HIST1H2AJ, ASF1B, TOP2A | 0.028839 | 2.00 |
| **Annotation Cluster 6 Enrichment Score: 2.38** | | | |  |
| GO:0001568~blood vessel development | 12 | PLAT, RECK, ANG, DHCR7, ITGAV, COL3A1, ITGA7, COL1A2, RHOB, COL1A1, COL5A1, THY1 | 4.41E-04 | 3.66 |
| GO:0001944~vasculature development | 12 | PLAT, RECK, ANG, DHCR7, ITGAV, COL3A1, ITGA7, COL1A2, RHOB, COL1A1, COL5A1, THY1 | 5.39E-04 | 3.57 |
| **Annotation Cluster 7 Enrichment Score: 2.03** | | | |  |
| GO:0007346~regulation of mitotic cell cycle | 8 | CDC6, DLGAP5, BIRC5, ANLN, UBE2C, CCNA2, GTSE1, CDK2 | 0.004219 | 3.93 |
| GO:0007096~regulation of exit from mitosis | 3 | BIRC5, ANLN, UBE2C | 0.010645 | 18.69 |
| GO:0010564~regulation of cell cycle process | 6 | DLGAP5, BIRC5, ANLN, CALR, UBE2C, GTSE1 | 0.018087 | 3.93 |
| **Annotation Cluster 8 Enrichment Score: 1.79** | | | |  |
| GO:0031589~cell-substrate adhesion | 6 | CORO1A, ITGAV, COL3A1, ITGA7, ITGA10, THY1 | 0.009902 | 4.58 |
| GO:0007229~integrin-mediated signaling pathway | 5 | ITGA5, ITGAV, COL3A1, ITGA7, ITGA10 | 0.014062 | 5.34 |
| GO:0007160~cell-matrix adhesion | 5 | ITGAV, COL3A1, ITGA7, ITGA10, THY1 | 0.030887 | 4.20 |

Table S5. Functional annotation clustering - Gene Ontology Terms for Molecular Functions: MSC-γ vs. UT-MSC (upregulated genes)

| **Term** | **Count** | **Genes** | **PValue** | **Fold Enrichment** |
| --- | --- | --- | --- | --- |
| **Annotation Cluster 1 Enrichment Score: 5.75** | | | | |
| GO:0016876~ligase activity, forming aminoacyl-tRNA and related compounds | 11 | IARS, WARS, YARS, CARS, NARS, ZNFX1, SARS, LARS, GARS, EPRS, MARS | 1.79E-06 | 7.30 |
| GO:0016875~ligase activity, forming carbon-oxygen bonds | 11 | IARS, WARS, YARS, CARS, NARS, ZNFX1, SARS, LARS, GARS, EPRS, MARS | 1.79E-06 | 7.30 |
| GO:0004812~aminoacyl-tRNA ligase activity | 11 | IARS, WARS, YARS, CARS, NARS, ZNFX1, SARS, LARS, GARS, EPRS, MARS | 1.79E-06 | 7.30 |
| **Annotation Cluster 2 Enrichment Score: 3.39** | | | | |
| GO:0000166~nucleotide binding | 106 | ADCY3, GRPEL2, ACSS3, RNF213, NLRC5, RAVER2, MLKL, DHX36, RAB27B, MX1, MX2, GTPBP2, CIITA, CARS, GBP6, YARS, GBP5, NAV3, RND3, RENBP, RIPK2, RAB12, EIF2AK2, GBP4, GBP3, SRXN1, GBP2, GBP1, HLA-DRA, IFIH1, ME2, ABCA9, ASS1, OAS3, OAS1, OAS2, RRAGC, CMPK2, SGK223, MOV10, IDH1, TUBE1, SUPV3L1, DYRK3, RHOBTB3, ITK, SMCHD1, RAB8B, MYO1B, DOCK9, GARS, EPRS, YTHDC2, U2AF1L4, DDX58, SCN8A, SLC27A1, SEPHS2, NARS, ZNFX1, CPEB4, SLFN5, HLCS, HLA-DMB, GSR, WARS, LONP1, DDX60, AGAP1, AIFM2, SARS, DAPK1, RPL23AP32, DHRS3, HIPK2, ERN1, TXNRD1, REV3L, MKNK2, ADH5, HK2, TRIB3, ASNS, GCH1, IARS, PDE1C, MAP3K1, TAP2, TAP1, LARS, CAMK2D, DMGDH, GUF1, MARS, ACSL5, HSPA9, ALPK1, ALPK2, STRADB, PCK2, RAB31, ABCC3, PHGDH, SLFN12, ABCC1, JAK2 | 1.87E-05 | 1.47 |
| GO:0017076~purine nucleotide binding | 93 | ADCY3, GRPEL2, SEPHS2, NARS, ZNFX1, HLCS, SLFN5, HLA-DMB, ACSS3, WARS, NLRC5, GSR, LONP1, DDX60, MLKL, DHX36, AGAP1, MX1, RAB27B, MX2, GTPBP2, CIITA, GBP6, CARS, YARS, GBP5, AIFM2, SARS, DAPK1, RND3, RENBP, HIPK2, ERN1, RIPK2, TXNRD1, RAB12, GBP4, EIF2AK2, GBP3, SRXN1, GBP2, GBP1, HLA-DRA, IFIH1, ABCA9, ASS1, MKNK2, HK2, OAS3, TRIB3, OAS1, ASNS, OAS2, CMPK2, RRAGC, GCH1, IARS, SGK223, MOV10, PDE1C, TAP2, MAP3K1, TAP1, LARS, CAMK2D, DMGDH, TUBE1, SUPV3L1, DYRK3, GUF1, MARS, RHOBTB3, ACSL5, HSPA9, ITK, ALPK1, SMCHD1, RAB8B, MYO1B, ALPK2, DOCK9, GARS, EPRS, YTHDC2, STRADB, PCK2, DDX58, RAB31, SLFN12, ABCC3, ABCC1, JAK2, SCN8A | 2.78E-05 | 1.51 |
| GO:0032555~purine ribonucleotide binding | 88 | ADCY3, SEPHS2, NARS, ZNFX1, HLCS, SLFN5, HLA-DMB, ACSS3, WARS, NLRC5, LONP1, DDX60, MLKL, DHX36, AGAP1, MX1, RAB27B, MX2, GTPBP2, CIITA, GBP6, CARS, YARS, GBP5, SARS, DAPK1, RND3, RENBP, HIPK2, ERN1, RIPK2, RAB12, GBP4, EIF2AK2, GBP3, GBP2, SRXN1, GBP1, HLA-DRA, IFIH1, ABCA9, ASS1, MKNK2, HK2, OAS3, TRIB3, OAS1, ASNS, OAS2, CMPK2, RRAGC, GCH1, IARS, SGK223, MOV10, PDE1C, TAP2, MAP3K1, TAP1, LARS, CAMK2D, TUBE1, SUPV3L1, DYRK3, GUF1, MARS, RHOBTB3, ACSL5, HSPA9, ITK, ALPK1, SMCHD1, RAB8B, MYO1B, ALPK2, DOCK9, GARS, EPRS, YTHDC2, STRADB, PCK2, DDX58, RAB31, SLFN12, ABCC3, ABCC1, JAK2, SCN8A | 7.82E-05 | 1.50 |
| GO:0032553~ribonucleotide binding | 88 | ADCY3, SEPHS2, NARS, ZNFX1, HLCS, SLFN5, HLA-DMB, ACSS3, WARS, NLRC5, LONP1, DDX60, MLKL, DHX36, AGAP1, MX1, RAB27B, MX2, GTPBP2, CIITA, GBP6, CARS, YARS, GBP5, SARS, DAPK1, RND3, RENBP, HIPK2, ERN1, RIPK2, RAB12, GBP4, EIF2AK2, GBP3, GBP2, SRXN1, GBP1, HLA-DRA, IFIH1, ABCA9, ASS1, MKNK2, HK2, OAS3, TRIB3, OAS1, ASNS, OAS2, CMPK2, RRAGC, GCH1, IARS, SGK223, MOV10, PDE1C, TAP2, MAP3K1, TAP1, LARS, CAMK2D, TUBE1, SUPV3L1, DYRK3, GUF1, MARS, RHOBTB3, ACSL5, HSPA9, ITK, ALPK1, SMCHD1, RAB8B, MYO1B, ALPK2, DOCK9, GARS, EPRS, YTHDC2, STRADB, PCK2, DDX58, RAB31, SLFN12, ABCC3, ABCC1, JAK2, SCN8A | 7.82E-05 | 1.50 |
| GO:0001883~purine nucleoside binding | 73 | ADCY3, GRPEL2, SEPHS2, NARS, ZNFX1, HLCS, SLFN5, HLA-DMB, ACSS3, WARS, NLRC5, GSR, LONP1, DDX60, DHX36, MLKL, CIITA, CARS, YARS, AIFM2, SARS, DAPK1, RENBP, HIPK2, ERN1, RIPK2, TXNRD1, EIF2AK2, SRXN1, HLA-DRA, IFIH1, ABCA9, ASS1, MKNK2, HK2, OAS3, TRIB3, OAS1, ASNS, OAS2, RRAGC, CMPK2, IARS, SGK223, MOV10, PDE1C, TAP2, MAP3K1, TAP1, LARS, CAMK2D, DMGDH, SUPV3L1, DYRK3, MARS, ACSL5, HSPA9, RHOBTB3, ITK, ALPK1, SMCHD1, MYO1B, ALPK2, GARS, EPRS, YTHDC2, STRADB, DDX58, SLFN12, ABCC3, ABCC1, JAK2, SCN8A | 0.001553 | 1.42 |
| GO:0030554~adenyl nucleotide binding | 72 | ADCY3, GRPEL2, SEPHS2, NARS, ZNFX1, HLCS, SLFN5, HLA-DMB, ACSS3, WARS, NLRC5, GSR, LONP1, DDX60, DHX36, MLKL, CIITA, CARS, YARS, AIFM2, SARS, DAPK1, RENBP, HIPK2, ERN1, RIPK2, TXNRD1, EIF2AK2, SRXN1, HLA-DRA, IFIH1, ABCA9, ASS1, MKNK2, HK2, OAS3, TRIB3, OAS1, ASNS, OAS2, CMPK2, IARS, SGK223, MOV10, PDE1C, TAP2, MAP3K1, TAP1, LARS, CAMK2D, DMGDH, SUPV3L1, DYRK3, MARS, ACSL5, HSPA9, RHOBTB3, ITK, ALPK1, SMCHD1, MYO1B, ALPK2, GARS, EPRS, YTHDC2, STRADB, DDX58, SLFN12, ABCC3, ABCC1, JAK2, SCN8A | 0.001645 | 1.42 |
| GO:0001882~nucleoside binding | 73 | ADCY3, GRPEL2, SEPHS2, NARS, ZNFX1, HLCS, SLFN5, HLA-DMB, ACSS3, WARS, NLRC5, GSR, LONP1, DDX60, DHX36, MLKL, CIITA, CARS, YARS, AIFM2, SARS, DAPK1, RENBP, HIPK2, ERN1, RIPK2, TXNRD1, EIF2AK2, SRXN1, HLA-DRA, IFIH1, ABCA9, ASS1, MKNK2, HK2, OAS3, TRIB3, OAS1, ASNS, OAS2, RRAGC, CMPK2, IARS, SGK223, MOV10, PDE1C, TAP2, MAP3K1, TAP1, LARS, CAMK2D, DMGDH, SUPV3L1, DYRK3, MARS, ACSL5, HSPA9, RHOBTB3, ITK, ALPK1, SMCHD1, MYO1B, ALPK2, GARS, EPRS, YTHDC2, STRADB, DDX58, SLFN12, ABCC3, ABCC1, JAK2, SCN8A | 0.001895 | 1.41 |
| GO:0032559~adenyl ribonucleotide binding | 67 | ADCY3, SEPHS2, NARS, ZNFX1, HLCS, SLFN5, HLA-DMB, ACSS3, WARS, NLRC5, LONP1, DDX60, DHX36, MLKL, CIITA, CARS, YARS, SARS, DAPK1, RENBP, HIPK2, ERN1, RIPK2, EIF2AK2, SRXN1, HLA-DRA, IFIH1, ABCA9, ASS1, MKNK2, HK2, OAS3, TRIB3, OAS1, ASNS, OAS2, CMPK2, IARS, SGK223, MOV10, PDE1C, TAP2, MAP3K1, TAP1, LARS, CAMK2D, SUPV3L1, DYRK3, MARS, ACSL5, HSPA9, RHOBTB3, ITK, ALPK1, SMCHD1, ALPK2, MYO1B, GARS, EPRS, YTHDC2, STRADB, DDX58, SLFN12, ABCC3, ABCC1, JAK2, SCN8A | 0.004036 | 1.40 |
| GO:0005524~ATP binding | 66 | ADCY3, SEPHS2, NARS, ZNFX1, HLCS, SLFN5, HLA-DMB, ACSS3, WARS, NLRC5, LONP1, DDX60, DHX36, MLKL, CIITA, CARS, YARS, SARS, DAPK1, RENBP, HIPK2, ERN1, RIPK2, EIF2AK2, SRXN1, HLA-DRA, IFIH1, ABCA9, ASS1, MKNK2, OAS3, HK2, TRIB3, OAS1, ASNS, OAS2, CMPK2, IARS, SGK223, MOV10, TAP2, MAP3K1, TAP1, LARS, CAMK2D, SUPV3L1, DYRK3, MARS, ACSL5, HSPA9, RHOBTB3, ITK, ALPK1, SMCHD1, ALPK2, MYO1B, GARS, EPRS, YTHDC2, STRADB, DDX58, SLFN12, ABCC3, ABCC1, JAK2, SCN8A | 0.004548 | 1.39 |
| **Annotation Cluster 3 Enrichment Score: 3.10** | | | | |
| GO:0005125~cytokine activity | 18 | SECTM1, NAMPT, IL6, CCL2, CSF1, CXCL9, CCL8, IL32, CXCL11, TGFB2, FLT3LG, CXCL10, TNFSF10, CCL13, TNFSF13B, CXCL16, VEGFA, GDF15 | 1.74E-04 | 2.88 |
| GO:0042379~chemokine receptor binding | 8 | YARS, CCL13, CCL2, CXCL16, CXCL9, CCL8, CXCL11, CXCL10 | 8.73E-04 | 5.10 |
| GO:0008009~chemokine activity | 7 | CCL13, CCL2, CXCL16, CXCL9, CCL8, CXCL11, CXCL10 | 0.0033 | 4.75 |
| **Annotation Cluster 4 Enrichment Score: 1.90** | | | | |
| GO:0003924~GTPase activity | 16 | GTPBP2, GBP6, GBP5, RAB8B, RRAGC, RND3, RAB31, TUBE1, GUF1, GBP4, MX1, RAB27B, MX2, GBP3, GBP2, GBP1 | 0.0033 | 2.37 |
| GO:0005525~GTP binding | 21 | GTPBP2, GBP6, GBP5, RAB8B, DOCK9, PCK2, RRAGC, GCH1, RND3, RAB31, TUBE1, RAB12, GUF1, AGAP1, GBP4, MX1, RAB27B, MX2, GBP3, GBP2, GBP1 | 0.0166 | 1.76 |
| GO:0019001~guanyl nucleotide binding | 21 | GTPBP2, GBP6, GBP5, RAB8B, DOCK9, PCK2, RRAGC, GCH1, RND3, RAB31, TUBE1, RAB12, GUF1, AGAP1, GBP4, MX1, RAB27B, MX2, GBP3, GBP2, GBP1 | 0.0214 | 1.72 |
| GO:0032561~guanyl ribonucleotide binding | 21 | GTPBP2, GBP6, GBP5, RAB8B, DOCK9, PCK2, RRAGC, GCH1, RND3, RAB31, TUBE1, RAB12, GUF1, AGAP1, GBP4, MX1, RAB27B, MX2, GBP3, GBP2, GBP1 | 0.0214 | 1.72 |
| **Annotation Cluster 5 Enrichment Score: 1.84** | | | | |
| GO:0003714~transcription corepressor activity | 13 | SP100, CBX4, RYBP, TRIB3, MXD1, TRIM22, MSC, DDIT3, ATF3, HIPK2, CREG1, ID4, NFIL3 | 0.0024 | 2.80 |
| GO:0016564~transcription repressor activity | 20 | CIITA, SP100, NACC2, ARID5B, CBX4, RYBP, TRIB3, IFI16, MXD1, MSC, TRIM22, DDIT3, ATF3, ID1, HIPK2, CREG1, MDM2, ID4, BCL6, NFIL3 | 0.0064 | 1.98 |
| GO:0003712~transcription cofactor activity | 19 | CIITA, SP100, HTATIP2, NMI, EPAS1, RYBP, CBX4, TRIB3, MXD1, MSC, TRIM22, DDIT3, JMY, ATF3, NCOA3, HIPK2, CREG1, ID4, NFIL3 | 0.0442 | 1.63 |
| GO:0008134~transcription factor binding | 24 | CIITA, NBN, SP100, HTATIP2, NMI, EPAS1, CFB, CEBPG, RYBP, CBX4, TRIB3, MXD1, MSC, TRIM22, DDIT3, JMY, ATF3, NCOA3, BCL2, HIPK2, CREG1, ID4, FOXC1, NFIL3 | 0.0625 | 1.46 |
| **Annotation Cluster 6 Enrichment Score: 1.67** | | | | |
| GO:0042803~protein homodimerization activity | 20 | GRPEL2, CARS, SP100, CEBPB, IL6ST, SMAD6, CSF1, ADH5, CALCOCO2, ASNS, GCH1, TGFB2, FLT3LG, GSR, RENBP, TAP2, BCL2, VEGFA, TAP1, CLIP1 | 0.0112 | 1.87 |
| GO:0046983~protein dimerization activity | 28 | GRPEL2, IL6ST, CSF1, ADH5, CALCOCO2, ASNS, GCH1, RRAGC, TGFB2, FLT3LG, GSR, BCL2, TAP2, TAP1, NFIL3, CARS, SP100, CEBPB, EPAS1, SCUBE1, CEBPG, SMAD6, DDIT3, RENBP, ATF3, VEGFA, CLIP1, NFE2L2 | 0.0150 | 1.61 |

Table S6 Functional annotation clustering - Gene Ontology Terms for Molecular Functions: MSC-γ vs. UT-MSC (downregulated genes)

| **Term** | **Count** | **Genes** | **P-Value** | **Fold Enrichment** |
| --- | --- | --- | --- | --- |
| **Annotation Cluster 1 Enrichment Score: 2.30** | | | | |
| GO:0019798~procollagen-proline dioxygenase activity | 4 | LEPRE1, LEPREL2, P4HA1, P4HA3 | 8.10E-05 | 42.15 |
| GO:0031543~peptidyl-proline dioxygenase activity | 4 | LEPRE1, LEPREL2, P4HA1, P4HA3 | 1.28E-04 | 36.88 |
| GO:0019842~vitamin binding | 8 | LEPRE1, LEPREL2, P4HA1, P4HA3, CRABP2, FASN, GCAT, PC | 0.0019 | 4.54 |
| GO:0031418~L-ascorbic acid binding | 4 | LEPRE1, LEPREL2, P4HA1, P4HA3 | 0.0027 | 14.05 |
| GO:0016706~oxidoreductase activity, acting on paired donors, with incorporation or reduction of molecular oxygen, 2-oxoglutarate as one donor, and incorporation of one atom each of oxygen into both donors | 4 | LEPRE1, LEPREL2, P4HA1, P4HA3 | 0.0062 | 10.54 |
| GO:0031406~carboxylic acid binding | 7 | LEPRE1, LEPREL2, P4HA1, P4HA3, CRABP2, FASN, PC | 0.0133 | 3.59 |
| **Annotation Cluster 2 Enrichment Score: 2.17** | | | | |
| GO:0030246~carbohydrate binding | 14 | GALNT1, ENPP1, ANG, LGALS1, CD248, CCDC80, VCAN, DCN, CALR, THBS2, FUCA1, COL5A1, CLEC11A, PRELP | 0.0010 | 2.92 |
| GO:0001871~pattern binding | 8 | ENPP1, ANG, CCDC80, VCAN, DCN, THBS2, COL5A1, PRELP | 0.0048 | 3.83 |
| GO:0030247~polysaccharide binding | 8 | ENPP1, ANG, CCDC80, VCAN, DCN, THBS2, COL5A1, PRELP | 0.0048 | 3.83 |
| GO:0005539~glycosaminoglycan binding | 7 | ANG, CCDC80, VCAN, DCN, THBS2, COL5A1, PRELP | 0.0117 | 3.69 |
| **Annotation Cluster 3 Enrichment Score: 2.10** | | | | |
| GO:0003756~protein disulfide isomerase activity | 3 | PDIA6, PDIA5, PDIA4 | 0.0061 | 24.59 |
| GO:0016864~intramolecular oxidoreductase activity, transposing S-S bonds | 3 | PDIA6, PDIA5, PDIA4 | 0.0061 | 24.59 |
| GO:0016862~intramolecular oxidoreductase activity, interconverting keto- and enol-groups | 3 | PDIA6, PDIA5, PDIA4 | 0.0076 | 22.13 |
| GO:0016860~intramolecular oxidoreductase activity | 4 | EBP, PDIA6, PDIA5, PDIA4 | 0.0144 | 7.76 |

Table S7. Functional annotation clustering- Gene Ontology Terms for Cellular components: MSC-γ vs. UT-MSC (upregulated genes)

| **Term** | **Count** | **Genes** | **P-Value** | **Fold Enrichment** |
| --- | --- | --- | --- | --- |
| **Annotation Cluster 1 Enrichment Score: 2.69** | | | | |
| GO:0005615~extracellular space | 39 | CCL2, IL6ST, C3, CSF1, CLU, CXCL9, KITLG, CCL8, TIMP4, IL32, CXCL11, CXCL10, FLT3LG, TGFB2, LGALS3BP, ISG15, IL15RA, C2, FGF2, SECTM1, ICAM1, YARS, IL6, C4A, LY96, C4B, SCUBE1, SERPING1, TNFSF10, CCL13, APOL1, TNFSF13B, CXCL16, VEGFA, C1RL, GDF15, TNFAIP2, IGFBP5, VLDLR | 6.27E-05 | 1.99 |
| GO:0044421~extracellular region part | 43 | FMOD, CCL2, IL6ST, C3, CSF1, CLU, CXCL9, KITLG, CCL8, TIMP4, IL32, CXCL11, CXCL10, FLT3LG, TGFB2, LGALS3BP, ISG15, IL15RA, C2, FGF2, SECTM1, ICAM1, YARS, IL6, C4A, LY96, C4B, SCUBE1, NTN4, SERPING1, ADAMTS9, TNFSF10, CCL13, APOL1, TNFSF13B, CXCL16, VEGFA, C1RL, GDF15, TNFAIP2, ADAMTS5, IGFBP5, VLDLR | 0.0035 | 1.57 |

Table S8. Functional annotation clustering- Gene Ontology Terms for Cellular components: MSC-γ vs. UT-MSC (downregulated genes)

| **Term** | **Count** | **Genes** | **P-Value** | **Fold Enrichment** |
| --- | --- | --- | --- | --- |
| **Annotation Cluster 1 Enrichment Score: 12.76** | | | | |
| GO:0005578~proteinaceous extracellular matrix | 34 | CTHRC1, CD248, LUM, COL3A1, ELN, SPOCK1, DCN, CALR, HMCN1, ANG, TGFBI, COL6A1, COL12A1, ADAMTS12, COL11A1, LOXL1, COL10A1, COL4A1, LGALS1, CCDC80, SPARC, COL5A2, COL5A1, PRELP, EMILIN1, LEPRE1, COL14A1, COL1A2, VCAN, MFAP2, COL1A1, MFAP4, MFAP5, ADAMTS2 | 1.87E-18 | 6.89 |
| GO:0031012~extracellular matrix | 35 | CTHRC1, LUM, CD248, COL3A1, ELN, SPOCK1, DCN, CALR, HMCN1, ANG, TGFBI, COL6A1, COL12A1, ADAMTS12, COL11A1, LOXL1, PRSS12, COL10A1, COL4A1, LGALS1, CCDC80, SPARC, COL5A2, COL5A1, PRELP, EMILIN1, LEPRE1, COL14A1, COL1A2, VCAN, MFAP2, COL1A1, MFAP4, MFAP5, ADAMTS2 | 2.23E-18 | 6.58 |
| GO:0044420~extracellular matrix part | 20 | COL4A1, LUM, COL3A1, CCDC80, SPARC, COL5A2, COL5A1, COL14A1, HMCN1, ANG, COL1A2, COL12A1, COL6A1, MFAP2, COL1A1, MFAP4, COL11A1, MFAP5, PRSS12, COL10A1 | 1.30E-14 | 11.09 |
| GO:0044421~extracellular region part | 41 | CTHRC1, FGF5, LDLR, ENPP1, LUM, CD248, COL3A1, ELN, SPOCK1, DCN, CALR, HMCN1, ANG, TGFBI, COL6A1, COL12A1, ADAMTS12, COL11A1, LOXL1, PRSS12, COL10A1, PLAT, COL4A1, LGALS1, CCDC80, SPARC, COL5A2, COL5A1, PRELP, SLIT3, EMILIN1, LEPRE1, COL14A1, COL1A2, GDF11, MFAP2, VCAN, COL1A1, MFAP4, MFAP5, ADAMTS2 | 4.56E-09 | 2.77 |
| GO:0005576~extracellular region | 59 | CTHRC1, FGF5, LDLR, VCL, OLFML3, WISP1, HMCN1, ANG, TGFBI, COL12A1, COL11A1, LOXL1, COL10A1, OLFML2B, PLAUR, SLIT3, PRELP, COL1A2, KRTAP1-1, MFAP2, VCAN, COL1A1, MFAP4, ADAM12, MFAP5, ADAMTS2, GALNT1, ENPP1, LUM, CD248, COL3A1, ELN, SPOCK1, DCN, CALR, MANF, FNDC1, GLIPR1, CDA, COL6A1, ADAMTS12, THBS2, PRSS12, PLAT, COL4A1, LGALS1, CCDC80, SPARC, COL5A2, COL5A1, CLEC11A, EMILIN1, C19ORF10, LEPRE1, COL14A1, SRPX2, PENK, CD59, GDF11 | 6.18E-07 | 1.90 |
| **Annotation Cluster 2 Enrichment Score: 11.16** | | | | |
| GO:0044420~extracellular matrix part | 20 | COL4A1, LUM, COL3A1, CCDC80, SPARC, COL5A2, COL5A1, COL14A1, HMCN1, ANG, COL1A2, COL12A1, COL6A1, MFAP2, COL1A1, MFAP4, COL11A1, MFAP5, PRSS12, COL10A1 | 1.30E-14 | 11.09 |
| GO:0005581~collagen | 12 | COL14A1, COL4A1, LUM, COL3A1, COL1A2, COL6A1, COL12A1, COL1A1, COL5A2, COL11A1, COL5A1, COL10A1 | 2.52E-12 | 22.25 |
| GO:0005583~fibrillar collagen | 7 | LUM, COL3A1, COL1A2, COL1A1, COL5A2, COL11A1, COL5A1 | 1.03E-08 | 37.85 |
| **Annotation Cluster 3 Enrichment Score: 2.01** | | | | |
| GO:0005694~chromosome | 18 | MKI67, HIST1H1C, HIST1H1B, BIRC5, AURKB, CDK2, RAD51, SPC24, HIST1H2BO, HIST2H2AB, NCAPH, CDCA8, HIST1H2BM, HJURP, SEC13, HIST1H2AJ, ASF1B, TOP2A | 7.23E-04 | 2.54 |
| GO:0044427~chromosomal part | 16 | MKI67, HIST1H1C, HIST1H1B, BIRC5, AURKB, CDK2, SPC24, HIST1H2BO, HIST2H2AB, NCAPH, CDCA8, HIST1H2BM, HJURP, SEC13, HIST1H2AJ, ASF1B | 9.01E-04 | 2.69 |
| GO:0000786~nucleosome | 6 | HIST1H2BO, HIST2H2AB, HIST1H2BM, HIST1H1C, HIST1H1B, HIST1H2AJ | 0.0028 | 6.18 |
| GO:0032993~protein-DNA complex | 6 | HIST1H2BO, HIST2H2AB, HIST1H2BM, HIST1H1C, HIST1H1B, HIST1H2AJ | 0.0103 | 4.53 |
| GO:0000775~chromosome, centromeric region | 7 | SPC24, CDCA8, MKI67, HJURP, SEC13, BIRC5, AURKB | 0.0121 | 3.66 |
| GO:0000793~condensed chromosome | 7 | SPC24, NCAPH, MKI67, HJURP, AURKB, CDK2, RAD51 | 0.0145 | 3.52 |
| **Annotation Cluster 4 Enrichment Score: 1.52** | | | | |
| GO:0015630~microtubule cytoskeleton | 18 | CDC6, KIFC1, KIF11, PRC1, TUBB2A, DLGAP5, TPX2, CDC20, BIRC5, AURKB, GTSE1, CCNB1, CDCA8, PLK1, TUBA1A, TUBA1B, TOP2A, KIF20A | 0.0047 | 2.13 |
| GO:0044430~cytoskeletal part | 25 | KIFC1, PRC1, LMNB1, TUBB2A, ANLN, AURKB, GTSE1, CDCA8, TUBA1A, TOP2A, TUBA1B, CDC6, KIF11, DLGAP5, TPX2, BIRC5, CDC20, KRTAP10-5, CCNB1, CORO1A, PLK1, FLG, KRT14, KRTAP1-1, KIF20A | 0.0109 | 1.70 |
| GO:0005856~cytoskeleton | 32 | KIFC1, PRC1, LMNB1, TUBB2A, ANLN, AURKB, GTSE1, VCL, CDCA8, TUBA1A, TUBA1B, STK38L, TOP2A, CDC6, KIF11, ACTA2, DLGAP5, MICAL2, FSCN1, TPX2, KRTAP10-5, BIRC5, CDC20, CCNB1, JUP, CORO1A, PLK1, FLG, KRT14, KRTAP1-1, FHOD1, KIF20A | 0.0191 | 1.50 |

**Table S9. Unmapped genes from the gene list entry for DAVID: MSC-17 vs. UT-MSC**

| **Gene Symbol** | **Gene name** | **mRNA Accession** | **Fold Change** | **P-value** | **Gene type** |
| --- | --- | --- | --- | --- | --- |
| **Upregulated genes:** | | | | | |
| [lnc-SULF2-2:2, lnc-SULF2-2:1](http://www.lncipedia.org/db/transcript/lnc-SULF2-2:2) |  | TCONS_00028418-XLOC_013771 | 2.66 | 0.0227 | lncRNA (non coding) |
| [lnc-GINS1-2:1](http://www.lncipedia.org/db/transcript/lnc-GINS1-2:1) |  | ENST00000376445 | 2.6 | 0.0373 | lncRNA (non coding) |
| [AC116562.1](https://database.riken.jp/sw/en/id/crib178s1rib178u221301i) |  | ENST00000408374 | 2.53 | 0.0260 | miRNA |
| RNA5SP191 | RNA, 5S ribosomal pseudogene 191 | ENST00000362585 | 2.52 | 0.0447 | rRNA (non coding) |
| RNY4P23 | RNA, Ro-associated Y4 pseudogene 23 | ENST00000364507 | 2.51 | 0.0470 |  |
| RNA5SP234 | RNA, 5S ribosomal pseudogene 234 | ENST00000363916 | 2.32 | 0.0164 |  |
| [U3](https://database.riken.jp/sw/en/id/crib178s1rib178u252805i) | Small nucleolar RNA U3 | ENST00000516996 | 2.14 | 0.0168 | snoRNA (non coding) |
| [Y_RNA](https://database.riken.jp/sw/en/id/crib178s1rib178u251986i) | Y RNA | [ENST00000516177](https://database.riken.jp/sw/en/id/crib178u3rib178u516177i) | 2.12 | 0.0414 | ncRNA (non coding) |
| [AC007365.2](https://database.riken.jp/sw/en/id/crib178s1rib178u253082i) |  | ENST00000517273 | 2.11 | 0.0120 | miRNA |
| RNA5SP249 | RNA, 5S ribosomal pseudogene 249 | ENST00000516127 | 2.11 | 0.0353 |  |
| SNORA70.1-201 | Small nucleolar RNA SNORA70 | ENST00000363367 | 2.09 | 0.0398 | snoRNA (non coding) |
| [U6](https://database.riken.jp/sw/en/id/crib178s1rib178u206944i) | U6 spliceosomal RNA | [ENST00000384217](https://database.riken.jp/sw/en/id/crib178u3rib178u384217i) | 2.08 | 0.0477 | snRNA |
| [BACH1](https://database.riken.jp/sw/en/id/crib178s1rib178u156273i) | BTB and CNC homology 1, basic leucine zipper transcription factor 1 | [ENST00000551628](https://database.riken.jp/sw/en/id/crib178u3rib178u551628i) | 2.06 | 0.0404 |  |
| RNA5SP176 | RNA, 5S ribosomal pseudogene 176 | ENST00000390956 | 2.06 | 0.0358 |  |
| RNU5E-9P | RNA, U5E small nuclear 9, pseudogene | ENST00000411164 | 2.05 | 0.0257 |  |
| **Downregulated Genes:** | | | | | |
| [RP11-265D17.2](https://database.riken.jp/sw/en/id/crib178s1rib178u254680i) |  | ENST00000527288 | -2.1 | 0.0415 | non coding |
|  |  | TCONS_l2_00026700-XLOC_l2_013898 | -2.29 | 0.0033 |  |
| OTTHUMG00000177628 | NULL; differential display clone 8 | ENST00000586713 | -2.3 | 0.0030 |  |
| MIR4476 | microRNA 4476 | NR_039687 | -2.33 | 0.0160 |  |
| RNU7-193P | U7 small nuclear RNA | ENST00000516723 | -2.41 | 0.0466 | snRNA (non coding) |
| [U6](https://database.riken.jp/sw/en/id/crib178s1rib178u252463i) | U6 spliceosomal RNA | ENST00000516654 | -2.57 | 0.0377 | non coding |
| [U6](https://database.riken.jp/sw/ja/id/crib178s1rib178u223037i) | U6 spliceosomal RNA | ENST00000411105 | -2.7 | 0.0349 | non coding |
| [AL109750.1](https://database.riken.jp/sw/en/id/crib178s1rib178u252430i) |  | ENST00000516621 | -2.87 | 0.0453 | miRNA |
| [Y_RNA](https://database.riken.jp/sw/en/id/crib178s1rib178u206651i) |  | [ENST00000383924](https://database.riken.jp/sw/en/id/crib178u3rib178u383924i) | -3.29 | 0.0432 | ncRNA (non coding) |

| **Table S10. Functional annotation clustering: Gene enrichment analysis on DAVID’s Default settings: MSC-17 vs. UT-MSC** | | | | | |
| --- | --- | --- | --- | --- | --- |
| **Category** | **Term** | **Count** | **Genes** | **P-Value** | **Fold Enrichment** |
| **Annotation Cluster 1 Enrichment Score: 3.58** | | | | | |
| GOTERM_CC_FAT | extracellular space | 10 | **Upregulated:** IL6, CCL2, C3, SAA1, SFRP4, CCL8, STC1, CXCL6, LBP, MMP13 | 1.09E-07 | 9.82 |
| GOTERM_CC_FAT | extracellular region part | 11 | **Upregulated:** IL6, CCL2, C3, SAA1, SFRP4, CCL8, STC1, CXCL6, LBP, MMP13, MMP1 | 1.37E-07 | 7.71 |
| GOTERM_BP_FAT | inflammatory response | 8 | **Upregulated:** NFKBIZ, IL6, CCL2, C3, SAA1, CCL8, CXCL6, LBP | 5.42E-07 | 14.48 |
| SP_PIR_KEYWORDS | Secreted | 12 | **Upregulated:** IL6, CCL2, C3, SAA1, SFRP4, CCL8, STC1, CXCL6, LBP, VMO1, MMP13, MMP1 | 1.97E-06 | 5.47 |
| GOTERM_BP_FAT | response to wounding | 8 | **Upregulated:** NFKBIZ, IL6, CCL2, C3, SAA1, CCL8, CXCL6, LBP | 1.39E-05 | 8.88 |
| GOTERM_CC_FAT | extracellular region | 12 | **Upregulated:** IL6, CCL2, C3, SAA1, SFRP4, CCL8, STC1, CXCL6, LBP, VMO1, MMP13, MMP1 | 1.54E-05 | 4.02 |
| GOTERM_BP_FAT | defense response | 8 | **Upregulated:** NFKBIZ, IL6, CCL2, C3, SAA1, CCL8, CXCL6, LBP | 3.64E-05 | 7.65 |
| GOTERM_BP_FAT | taxis | 5 | **Upregulated:** IL6, CCL2, SAA1, CCL8, CXCL6 | 1.17E-04 | 18.38 |
| GOTERM_BP_FAT | chemotaxis | 5 | **Upregulated:** IL6, CCL2, SAA1, CCL8, CXCL6 | 1.17E-04 | 18.38 |
| SP_PIR_KEYWORDS | signal | 13 | **Upregulated:** IL6, CCL2, SAA1, C3, SFRP4, CCL8, STC1, CXCL6, LBP, VMO1, MMP13, MMP1  **Downregulated:** ITGA6 | 1.92E-04 | 3.08 |
| UP_SEQ_FEATURE | signal peptide | 13 | **Upregulated:** IL6, CCL2, SAA1, C3, SFRP4, CCL8, STC1, CXCL6, LBP, VMO1, MMP13, MMP1  **Downregulated:** ITGA6, | 2.04E-04 | 3.06 |
| GOTERM_BP_FAT | locomotory behavior | 5 | **Upregulated:** IL6, CCL2, SAA1, CCL8, CXCL6 | 9.03E-04 | 10.73 |
| UP_SEQ_FEATURE | disulfide bond | 11 | **Upregulated:** IL6, CCL2, C3, SFRP4, CCL8, STC1, CXCL6, VMO1, MMP13, MMP1  **Downregulated:** ITGA6, | 0.0013 | 2.98 |
| SP_PIR_KEYWORDS | disulfide bond | 11 | **Upregulated:** IL6, CCL2, C3, SFRP4, CCL8, STC1, CXCL6, VMO1, MMP13, MMP1  **Downregulated:** ITGA6, | 0.0016 | 2.89 |
| GOTERM_BP_FAT | immune response | 6 | **Upregulated:** IL6, CCL2, C3, CCL8, CXCL6, LBP | 0.0043 | 5.11 |
| GOTERM_BP_FAT | behavior | 5 | **Upregulated:** IL6, CCL2, SAA1, CCL8, CXCL6 | 0.0063 | 6.27 |
| GOTERM_BP_FAT | positive regulation of multicellular organismal process | 4 | **Upregulated:** IL6, CCL2, SAA1, LBP | 0.0069 | 9.64 |
| UP_SEQ_FEATURE | glycosylation site:N-linked (GlcNAc...) | 11 | **Upregulated:** IL6, CCL2, C3, CH25H, SFRP4, SLC22A3, STC1, LBP, MMP13, MMP1  **Downregulated:** ITGA6, | 0.0213 | 2.04 |
| SP_PIR_KEYWORDS | glycoprotein | 11 | **Upregulated:** CCL2, C3, CH25H, SFRP4, SLC22A3, STC1, LBP, MMP13, MMP1  **Downregulated:** ITGA6 | 0.0276 | 1.96 |
| **Annotation Cluster 2 Enrichment Score: 2.58** | | | | | |
| GOTERM_BP_FAT | leukocyte migration | 4 | **Upregulated:** IL6, CCL2, SAA1  **Downregulated:** ITGA6, | 1.03E-04 | 41.28 |
| GOTERM_BP_FAT | taxis | 5 | **Upregulated:** IL6, CCL2, SAA1, CCL8, CXCL6 | 1.17E-04 | 18.38 |
| GOTERM_BP_FAT | chemotaxis | 5 | **Upregulated:** IL6, CCL2, SAA1, CCL8, CXCL6 | 1.17E-04 | 18.38 |
| GOTERM_BP_FAT | locomotory behavior | 5 | **Upregulated:** IL6, CCL2, SAA1, CCL8, CXCL6 | 9.03E-04 | 10.73 |
| GOTERM_BP_FAT | GO:0030595~leukocyte chemotaxis | 3 | **Upregulated:** IL6, CCL2, SAA1 | 0.0016 | 47.69 |
| GOTERM_BP_FAT | GO:0060326~cell chemotaxis | 3 | **Upregulated:** IL6, CCL2, SAA1 | 0.0018 | 45.24 |
| GOTERM_BP_FAT | GO:0007610~behavior | 5 | **Upregulated:** IL6, CCL2, SAA1, CCL8, CXCL6 | 0.0063 | 6.27 |
| GOTERM_BP_FAT | GO:0051240~positive regulation of multicellular organismal process | 4 | **Upregulated:** IL6, CCL2, SAA1, LBP | 0.0069 | 9.64 |
| GOTERM_BP_FAT | GO:0051240~cell migration | 4 | **Upregulated:** IL6, CCL2, SAA1  **Downregulated:** ITGA6, | 0.0097 | 8.52 |
| GOTERM_BP_FAT | GO:0051674~localization of cell | 4 | **Upregulated:** IL6, CCL2, SAA1  **Downregulated:** ITGA6, | 0.0129 | 7.66 |
| GOTERM_BP_FAT | GO:0048870~cell motility | 4 | **Upregulated:** IL6, CCL2, SAA1  **Downregulated:** ITGA6, | 0.0129 | 7.66 |
| GOTERM_BP_FAT | GO:0042592~homeostatic process | 5 | **Upregulated:** IL6, CCL2, SAA1, STC1  **Downregulated:** RPS24 | 0.0310 | 3.92 |
| GOTERM_BP_FAT | GO:0006928~cell motion | 4 | **Upregulated:** IL6, CCL2, SAA1  **Downregulated:** ITGA6, | 0.0404 | 4.95 |
| **Annotation Cluster 3 Enrichment Score: 2.48** | | | | | |
| GOTERM_BP_FAT | GO:0002675~positive regulation of acute inflammatory response | 3 | **Upregulated:** IL6, C3, LBP | 1.65E-04 | 147.04 |
| GOTERM_BP_FAT | GO:0050727~regulation of inflammatory response | 4 | **Upregulated:** IL6, C3, SAA1, LBP | 2.43E-04 | 30.96 |
| GOTERM_BP_FAT | GO:0002526~acute inflammatory response | 4 | **Upregulated:** IL6, C3, SAA1, LBP | 5.14E-04 | 24.01 |
| GOTERM_BP_FAT | GO:0002673~regulation of acute inflammatory response | 3 | **Upregulated:** IL6, C3, LBP | 5.20E-04 | 84.02 |
| SP_PIR_KEYWORDS | acute phase | 3 | **Upregulated:** IL6, C3, SAA1 | 5.93E-04 | 79.59 |
| GOTERM_BP_FAT | GO:0050729~positive regulation of inflammatory response | 3 | **Upregulated:** IL6, C3, LBP | 0.0011 | 58.82 |
| GOTERM_BP_FAT | GO:0032101~regulation of response to external stimulus | 4 | **Upregulated:** IL6, C3, SAA1, LBP | 0.0021 | 14.80 |
| GOTERM_BP_FAT | GO:0006955~immune response | 6 | **Upregulated:** IL6, CCL2, C3, CCL8, CXCL6, LBP | 0.0043 | 5.11 |
| GOTERM_BP_FAT | GO:0032103~positive regulation of response to external stimulus | 3 | **Upregulated:** IL6, C3, LBP | 0.0048 | 27.57 |
| GOTERM_BP_FAT | GO:0031349~positive regulation of defense response | 3 | **Upregulated:** IL6, C3, LBP | 0.0062 | 24.17 |
| GOTERM_BP_FAT | GO:0002697~regulation of immune effector process | 3 | **Upregulated:** IL6, C3, LBP | 0.0116 | 17.47 |
| GOTERM_BP_FAT | immune effector process | 3 | **Upregulated:** IL6, C3, LBP | 0.0198 | 13.17 |
| **Annotation Cluster 4 Enrichment Score: 2.38** | | | | | |
| GOTERM_BP_FAT | GO:0050727~regulation of inflammatory response | 4 | **Upregulated:** IL6, C3, SAA1, LBP | 2.43E-04 | 30.96 |
| GOTERM_BP_FAT | GO:0002526~acute inflammatory response | 4 | **Upregulated:** IL6, C3, SAA1, LBP | 5.14E-04 | 24.01 |
| GOTERM_BP_FAT | GO:0006953~acute-phase response | 3 | **Upregulated:** IL6, SAA1, LBP | 0.0019 | 44.11 |
| GOTERM_BP_FAT | GO:0032101~regulation of response to external stimulus | 4 | **Upregulated:** IL6, C3, SAA1, LBP | 0.0021 | 14.80 |
| GOTERM_BP_FAT | GO:0051240~positive regulation of multicellular organismal process | 4 | **Upregulated:** IL6, CCL2, SAA1, LBP | 0.0069 | 9.64 |
| GOTERM_BP_FAT | GO:0001819~positive regulation of cytokine production | 3 | **Upregulated:** IL6, SAA1, LBP | 0.0093 | 19.61 |
| GOTERM_BP_FAT | GO:0001817~regulation of cytokine production | 3 | **Upregulated:** IL6, SAA1, LBP | 0.0345 | 9.75 |
| **Annotation Cluster 5 Enrichment Score: 2.31** | | | | | |
| GOTERM_BP_FAT | GO:0042330~taxis | 5 | **Upregulated:** IL6, CCL2, SAA1, CCL8, CXCL6 | 1.17E-04 | 18.38 |
| GOTERM_BP_FAT | GO:0006935~chemotaxis | 5 | **Upregulated:** IL6, CCL2, SAA1, CCL8, CXCL6 | 1.17E-04 | 18.38 |
| GOTERM_BP_FAT | GO:0007626~locomotory behavior | 5 | **Upregulated:** IL6, CCL2, SAA1, CCL8, CXCL6 | 9.03E-04 | 10.73 |
| INTERPRO | IPR001811:Small chemokine, interleukin-8-like | 3 | **Upregulated:** CCL2, CCL8, CXCL6 | 0.0013 | 53.00 |
| SP_PIR_KEYWORDS | cytokine | 4 | **Upregulated:** IL6, CCL2, CCL8, CXCL6 | 0.0014 | 17.00 |
| SMART | SM00199:SCY | 3 | **Upregulated:** CCL2, CCL8, CXCL6 | 0.0017 | 44.29 |
| GOTERM_MF_FAT | GO:0008009~chemokine activity | 3 | **Upregulated:** CCL2, CCL8, CXCL6 | 0.0020 | 42.34 |
| GOTERM_MF_FAT | GO:0042379~chemokine receptor binding | 3 | **Upregulated:** CCL2, CCL8, CXCL6 | 0.0023 | 39.74 |
| GOTERM_MF_FAT | GO:0005125~cytokine activity | 4 | **Upregulated:** IL6, CCL2, CCL8, CXCL6 | 0.0027 | 13.32 |
| SP_PIR_KEYWORDS | chemotaxis | 3 | **Upregulated:** CCL2, CCL8, CXCL6 | 0.0036 | 32.06 |
| GOTERM_BP_FAT | GO:0006955~immune response | 6 | **Upregulated:** IL6, CCL2, C3, CCL8, CXCL6, LBP | 0.0043 | 5.11 |
| KEGG_PATHWAY | hsa04621:NOD-like receptor signaling pathway | 3 | **Upregulated:** IL6, CCL2, CCL8 | 0.0062 | 22.37 |
| GOTERM_BP_FAT | GO:0007610~behavior | 5 | **Upregulated:** IL6, CCL2, SAA1, CCL8, CXCL6 | 0.0063 | 6.27 |
| GOTERM_BP_FAT | GO:0051240~positive regulation of multicellular organismal process | 4 | **Upregulated:** IL6, CCL2, SAA1, LBP | 0.0069 | 9.64 |
| GOTERM_MF_FAT | GO:0008201~heparin binding | 3 | **Upregulated:** CCL2, CCL8, CXCL6 | 0.0098 | 18.91 |
| KEGG_PATHWAY | hsa04060:Cytokine-cytokine receptor interaction | 4 | **Upregulated:** IL6, CCL2, CCL8, CXCL6 | 0.0124 | 7.06 |
| GOTERM_BP_FAT | GO:0007267~cell-cell signaling | 5 | **Upregulated:** IL6, CCL8, SLC22A3, STC1, CXCL6 | 0.0148 | 4.90 |
| GOTERM_MF_FAT | GO:0005539~glycosaminoglycan binding | 3 | **Upregulated:** CCL2, CCL8, CXCL6 | 0.0175 | 13.91 |
| GOTERM_MF_FAT | GO:0001871~pattern binding | 3 | **Upregulated:** CCL2, CCL8, CXCL6 | 0.0209 | 12.65 |
| GOTERM_MF_FAT | GO:0030247~polysaccharide binding | 3 | **Upregulated:** CCL2, CCL8, CXCL6 | 0.0209 | 12.65 |
| BBID | 109.Chemokine_families | 3 | **Upregulated:** CCL2, CCL8, CXCL6 | 0.0412 | 7.16 |
| KEGG_PATHWAY | hsa04062:Chemokine signaling pathway | 3 | **Upregulated:** CCL2, CCL8, CXCL6 | 0.0498 | 7.42 |
| **Annotation Cluster 6 Enrichment Score: 1.76** | | | | | |
| GOTERM_BP_FAT | GO:0006955~immune response | 6 | **Upregulated:** IL6, CCL2, C3, CCL8, CXCL6, LBP | 0.0043 | 5.11 |
| GOTERM_BP_FAT | GO:0051240~positive regulation of multicellular organismal process | 4 | **Upregulated:** IL6, CCL2, SAA1, LBP | 0.0069 | 9.64 |
| GOTERM_BP_FAT | GO:0002237~response to molecule of bacterial origin | 3 | **Upregulated:** IL6, CCL2, LBP | 0.0085 | 20.52 |
| GOTERM_BP_FAT | GO:0010033~response to organic substance | 5 | **Upregulated:** IL6, CCL2, LBP, STEAP2, MMP13 | 0.0272 | 4.08 |
| GOTERM_BP_FAT | GO:0009617~response to bacterium | 3 | **Upregulated:** IL6, CCL2, LBP | 0.0388 | 9.14 |
| **Annotation Cluster 7 Enrichment Score: 1.60** | | | | | |
| GOTERM_BP_FAT | GO:0006955~immune response | 6 | **Upregulated:** IL6, CCL2, C3, CCL8, CXCL6, LBP | 0.0043 | 5.11 |
| GOTERM_BP_FAT | GO:0006959~humoral immune response | 3 | **Upregulated:** IL6, CCL2, C3 | 0.0072 | 22.34 |
| BBID | 18.Cytokine_astocytes | 3 | **Upregulated:** IL6, CCL2, C3 | 0.0173 | 11.19 |
| **Annotation Cluster 8 Enrichment Score: 1.56** | | | | | |
| GOTERM_BP_FAT | GO:0009725~response to hormone stimulus | 4 | **Upregulated:** IL6, CCL2, STEAP2, MMP13 | 0.0208 | 6.41 |
| GOTERM_BP_FAT | GO:0009719~response to endogenous stimulus | 4 | **Upregulated:** IL6, CCL2, STEAP2, MMP13 | 0.0269 | 5.81 |
| GOTERM_BP_FAT | GO:0010033~response to organic substance | 5 | **Upregulated:** IL6, CCL2, LBP, STEAP2, MMP13 | 0.0272 | 4.08 |
| GOTERM_BP_FAT | GO:0048545~response to steroid hormone stimulus | 3 | **Upregulated:** IL6, CCL2, MMP13 | 0.0384 | 9.19 |

Table S11. Functional annotation clustering - Gene Ontology Terms for Biological Processes: MSC-17 vs. UT-MSC

| **GOTERM ID** | **GO Term** | **Count** | **Genes** | **Fold Enrichment** | **P-Value** |
| --- | --- | --- | --- | --- | --- |
| **Annotation Cluster 1 Enrichment Score: 3.13** | | | | | |
| GO:0006954 | inflammatory response | 8 | **Upregulated:** NFKBIZ, IL6, CCL2, C3, SAA1, CCL8, CXCL6, LBP | 14.48 | 5.42E-07 |
| GO:0009611 | response to wounding | 8 | **Upregulated:** NFKBIZ, IL6, CCL2, C3, SAA1, CCL8, CXCL6, LBP | 8.88 | 1.39E-05 |
| GO:0006952 | defense response | 8 | **Upregulated:** NFKBIZ, IL6, CCL2, C3, SAA1, CCL8, CXCL6, LBP | 7.65 | 3.64E-05 |
| GO:0006935 | chemotaxis | 5 | **Upregulated:** IL6, CCL2, SAA1, CCL8, CXCL6 | 18.38 | 1.17E-04 |
| GO:0042330 | taxis | 5 | **Upregulated:** IL6, CCL2, SAA1, CCL8, CXCL6 | 18.38 | 1.17E-04 |
| GO:0050727 | regulation of inflammatory response | 4 | **Upregulated:** IL6, C3, SAA1, LBP | 30.96 | 2.43E-04 |
| GO:0002526 | acute inflammatory response | 4 | **Upregulated:** IL6, C3, SAA1, LBP | 24.01 | 5.14E-04 |
| GO:0007626 | locomotory behavior | 5 | **Upregulated:** IL6, CCL2, SAA1, CCL8, CXCL6 | 10.73 | 9.03E-04 |
| GO:0006953 | acute-phase response | 3 | **Upregulated:** IL6, SAA1, LBP | 44.11 | 0.0019 |
| GO:0032101 | regulation of response to external stimulus | 4 | **Upregulated:** IL6, C3, SAA1, LBP | 14.80 | 0.0021 |
| GO:0006955 | immune response | 6 | **Upregulated:** IL6, CCL2, C3, CCL8, CXCL6, LBP | 5.11 | 0.0043 |
| GO:0007610 | behavior | 5 | **Upregulated:** IL6, CCL2, SAA1, CCL8, CXCL6 | 6.27 | 0.0063 |
| GO:0051240 | positive regulation of multicellular organismal process | 4 | **Upregulated:** IL6, CCL2, SAA1, LBP | 9.64 | 0.0069 |
| GO:0001819 | positive regulation of cytokine production | 3 | **Upregulated:** IL6, SAA1, LBP | 19.61 | 0.0093 |
| GO:0001817 | regulation of cytokine production | 3 | **Upregulated:** IL6, SAA1, LBP | 9.75 | 0.0345 |
| **Annotation Cluster 2 Enrichment Score: 2.51** | | | | | |
| GO:0050900 | leukocyte migration | 4 | **Upregulated:** IL6, CCL2, SAA1  **Downregulated:** ITGA6, | 41.28 | 1.03E-04 |
| GO:0006935 | chemotaxis | 5 | **Upregulated:** IL6, CCL2, SAA1, CCL8, CXCL6 | 18.38 | 1.17E-04 |
| GO:0042330 | taxis | 5 | **Upregulated:** IL6, CCL2, SAA1, CCL8, CXCL6 | 18.38 | 1.17E-04 |
| GO:0007626 | locomotory behavior | 5 | **Upregulated:** IL6, CCL2, SAA1, CCL8, CXCL6 | 10.73 | 9.03E-04 |
| GO:0030595 | leukocyte chemotaxis | 3 | **Upregulated:** IL6, CCL2, SAA1 | 47.69 | 0.0016 |
| GO:0060326 | cell chemotaxis | 3 | **Upregulated:** IL6, CCL2, SAA1 | 45.24 | 0.0018 |
| GO:0060326 | immune response | 6 | **Upregulated:** IL6, CCL2, C3, CCL8, CXCL6, LBP | 5.11 | 0.0043 |
| GO:0007610 | behavior | 5 | **Upregulated:** IL6, CCL2, SAA1, CCL8, CXCL6 | 6.27 | 0.0063 |
| GO:0051240 | positive regulation of multicellular organismal process | 4 | **Upregulated:** IL6, CCL2, SAA1, LBP | 9.64 | 0.0069 |
| GO:0016477 | cell migration | 4 | **Upregulated:** IL6, CCL2, SAA1  **Downregulated:** ITGA6, | 8.52 | 0.0097 |
| GO:004887 | cell motility | 4 | **Upregulated:** IL6, CCL2, SAA1  **Downregulated:** ITGA6 | 7.66 | 0.0129 |
| GO:0051674 | localization of cell | 4 | **Upregulated:** IL6, CCL2, SAA1  **Downregulated:** ITGA6 | 7.66 | 0.0129 |
| GO:000726 | cell-cell signaling | 5 | **Upregulated:** IL6, CCL8, SLC22A3, STC1, CXCL6 | 4.90 | 0.0148 |
| GO:0042592 | homeostatic process | 5 | **Upregulated:** IL6, CCL2, SAA1, STC1  **Downregulated:** RPS24 | 3.92 | 0.0310 |
| GO:0006928 | cell motion | 4 | **Upregulated:** IL6, CCL2, SAA1  **Downregulated:** ITGA6 | 4.95 | 0.0404 |
| **Annotation Cluster 3 Enrichment Score: 2.41** | | | | | |
| GO:0002675 | positive regulation of acute inflammatory response | 3 | **Upregulated:** IL6, C3, LBP | 147.04 | 1.65E-04 |
| GO:0050727 | regulation of inflammatory response | 4 | **Upregulated:** IL6, C3, SAA1, LBP | 30.96 | 2.43E-04 |
| GO:0002526 | acute inflammatory response | 4 | **Upregulated:** IL6, C3, SAA1, LBP | 24.01 | 5.14E-04 |
| GO:0002673 | regulation of acute inflammatory response | 3 | **Upregulated:** IL6, C3, LBP | 84.02 | 5.20E-04 |
| GO:0050729 | positive regulation of inflammatory response | 3 | **Upregulated:** IL6, C3, LBP | 58.82 | 0.0011 |
| GO:0032101 | regulation of response to external stimulus | 4 | **Upregulated:** IL6, C3, SAA1, LBP | 14.80 | 0.0021 |
| GO:0006955 | immune response | 6 | **Upregulated:** IL6, CCL2, C3, CCL8, CXCL6, LBP | 5.11 | 0.0043 |
| GO:0032103 | positive regulation of response to external stimulus | 3 | **Upregulated:** IL6, C3, LBP | 27.57 | 0.0048 |
| GO:0031349 | positive regulation of defense response | 3 | **Upregulated:** IL6, C3, LBP | 24.17 | 0.0062 |
| GO:005124 | positive regulation of multicellular organismal process | 4 | **Upregulated:** IL6, CCL2, SAA1, LBP | 9.64 | 0.0069 |
| GO:0002697 | regulation of immune effector process | 3 | **Upregulated:** IL6, C3, LBP | 17.47 | 0.0116 |
| GO:0002252 | immune effector process | 3 | **Upregulated:** IL6, C3, LBP | 13.17 | 0.0198 |
| **Annotation Cluster 4 Enrichment Score: 1.76** | | | | | |
| GO:0006955 | immune response | 6 | **Upregulated:** IL6, CCL2, C3, CCL8, CXCL6, LBP | 5.11 | 0.0043 |
| GO:0051240 | positive regulation of multicellular organismal process | 4 | **Upregulated:** IL6, CCL2, SAA1, LBP | 9.64 | 0.0069 |
| GO:0002237 | response to molecule of bacterial origin | 3 | **Upregulated:** IL6, CCL2, LBP | 20.52 | 0.0085 |
| GO:0010033 | response to organic substance | 5 | **Upregulated:** IL6, CCL2, LBP, STEAP2, MMP13 | 4.08 | 0.0272 |
| GO:0009617 | response to bacterium | 3 | **Upregulated:** IL6, CCL2, LBP | 9.14 | 0.0388 |
| **Annotation Cluster 5 Enrichment Score: 1.56** | | | | | |
| GO:0009725 | response to hormone stimulus | 4 | **Upregulated:** IL6, CCL2, STEAP2, MMP13 | 6.41 | 0.0208 |
| GO:0009719 | response to endogenous stimulus | 4 | **Upregulated:** IL6, CCL2, STEAP2, MMP13 | 5.81 | 0.0269 |
| GO:0010033 | response to organic substance | 5 | **Upregulated:** IL6, CCL2, LBP, STEAP2, MMP13 | 4.08 | 0.0272 |
| GO:0048545 | response to steroid hormone stimulus | 3 | **Upregulated:** IL6, CCL2, MMP13 | 9.19 | 0.0384 |

Table S12 Functional annotation clustering - Gene Ontology Terms for Molecular Functions: MSC-17 vs. UT-MSC

| **GOTERM ID** | **Term** | **Count** | **Genes** | **Fold Enrichment** | **P-Value** |
| --- | --- | --- | --- | --- | --- |
| **Annotation Cluster 1 Enrichment Score: 2.01** | | | | | |
| GO:0008009 | chemokine activity | 3 | **Upregulated:** CCL2, CCL8, CXCL6 | 42.34 | 0.0020 |
| GO:0042379 | chemokine receptor binding | 3 | **Upregulated:** CCL2, CCL8, CXCL6 | 39.74 | 0.0023 |
| GO:0005125~ | cytokine activity | 4 | **Upregulated:** IL6, CCL2, CCL8, CXCL6 | 13.32 | 0.0027 |
| GO:0008201 | heparin binding | 3 | **Upregulated:** CCL2, CCL8, CXCL6 | 18.91 | 0.0098 |
| GO:0005539 | glycosaminoglycan binding | 3 | **Upregulated:** CCL2, CCL8, CXCL6 | 13.91 | 0.0175 |
| GO:0001871 | pattern binding | 3 | **Upregulated:** CCL2, CCL8, CXCL6 | 12.65 | 0.0209 |
| GO:0030247 | polysaccharide binding | 3 | **Upregulated:** CCL2, CCL8, CXCL6 | 12.65 | 0.0209 |

Table S13 Functional annotation clustering – Gene Ontology Terms for Cellular Components: MSC-17 vs. UT-MSC

| **GOTERM ID** | **Term** | **Count** | **Genes** | **Fold Enrichment** | **P-Value** |
| --- | --- | --- | --- | --- | --- |
| **Annotation Cluster 1 Enrichment Score: 6.21** | | | | | |
| GO:0005615 | extracellular space | 10 | **Upregulated:** IL6, CCL2, C3, SAA1, SFRP4, CCL8, STC1, CXCL6, LBP, MMP13 | 9.82 | 1.09E-07 |
| GO:0044421 | extracellular region part | 11 | **Upregulated:** IL6, CCL2, C3, SAA1, SFRP4, CCL8, STC1, CXCL6, LBP, MMP13, MMP1 | 7.71 | 1.37E-07 |
| GO:0005576 | extracellular region | 12 | **Upregulated:** IL6, CCL2, C3, SAA1, SFRP4, CCL8, STC1, CXCL6, LBP, VMO1, MMP13, MMP1 | 4.02 | 1.54E-05 |

| **Table S14 Functional annotation clustering- Gene Ontology Terms for Biological Process: MSC-17 vs. MSC-γ (upregulated genes)** | | | | | |
| --- | --- | --- | --- | --- | --- |
| **GOTERM ID** | **GO TERM** | **Count** | **Genes** | **Fold Enrichment** | **P-Value** |
| **Annotation Cluster 1 Enrichment Score: 7.35** | | | | | |
| GO:0006334 | nucleosome assembly | 14 | HIST1H2AB, HIST4H4, HIST1H4L, HIST1H4K, HIST1H2AG, HIST1H2AD, HIST1H2AE, HIST2H4A, HIST2H4B, HIST1H2BO, HIST2H2AB, HIST1H2BM, HIST1H2BN, HIST1H4A, HIST1H4B, HIST1H2BI, HIST1H4E, HIST1H4F, HIST1H4C, HIST1H4D, HIST1H4I, HIST1H4J, HIST1H4H, HIST2H3A, HIST1H2BB, HIST1H3J, HIST1H2BC, HIST1H1E, HIST1H2BE, HIST1H1C, HIST1H2BF, HIST1H1B, HIST1H2BG, HIST2H3C, HIST2H3D, HIST1H3A, HIST1H2AI, HIST1H3B, HIST1H2AH, HIST1H3C, HIST1H2AK, HIST1H3D, HIST1H3E, HIST1H2AJ, HIST1H2AM, HIST1H3F, HIST1H2AL, HIST1H3G, HIST1H3H, HIST1H3I | 13.75 | 2.08E-11 |
| GO:0031497 | chromatin assembly | 14 | HIST1H2AB, HIST4H4, HIST1H4L, HIST1H4K, HIST1H2AG, HIST1H2AD, HIST1H2AE, HIST2H4A, HIST2H4B, HIST1H2BO, HIST2H2AB, HIST1H2BM, HIST1H2BN, HIST1H4A, HIST1H4B, HIST1H2BI, HIST1H4E, HIST1H4F, HIST1H4C, HIST1H4D, HIST1H4I, HIST1H4J, HIST1H4H, HIST2H3A, HIST1H2BB, HIST1H3J, HIST1H2BC, HIST1H1E, HIST1H2BE, HIST1H1C, HIST1H2BF, HIST1H1B, HIST1H2BG, HIST2H3C, HIST2H3D, HIST1H3A, HIST1H2AI, HIST1H3B, HIST1H2AH, HIST1H3C, HIST1H2AK, HIST1H3D, HIST1H3E, HIST1H2AJ, HIST1H2AM, HIST1H3F, HIST1H2AL, HIST1H3G, HIST1H3H, HIST1H3I | 13.27 | 3.30E-11 |
| GO:0065004 | protein-DNA complex assembly | 14 | HIST1H2AB, HIST4H4, HIST1H4L, HIST1H4K, HIST1H2AG, HIST1H2AD, HIST1H2AE, HIST2H4A, HIST2H4B, HIST1H2BO, HIST2H2AB, HIST1H2BM, HIST1H2BN, HIST1H4A, HIST1H4B, HIST1H2BI, HIST1H4E, HIST1H4F, HIST1H4C, HIST1H4D, HIST1H4I, HIST1H4J, HIST1H4H, HIST2H3A, HIST1H2BB, HIST1H3J, HIST1H2BC, HIST1H1E, HIST1H2BE, HIST1H1C, HIST1H2BF, HIST1H1B, HIST1H2BG, HIST2H3C, HIST2H3D, HIST1H3A, HIST1H2AI, HIST1H3B, HIST1H2AH, HIST1H3C, HIST1H2AK, HIST1H3D, HIST1H3E, HIST1H2AJ, HIST1H2AM, HIST1H3F, HIST1H2AL, HIST1H3G, HIST1H3H, HIST1H3I | 12.69 | 5.93E-11 |
| GO:0034728 | nucleosome organization | 14 | HIST1H2AB, HIST4H4, HIST1H4L, HIST1H4K, HIST1H2AG, HIST1H2AD, HIST1H2AE, HIST2H4A, HIST2H4B, HIST1H2BO, HIST2H2AB, HIST1H2BM, HIST1H2BN, HIST1H4A, HIST1H4B, HIST1H2BI, HIST1H4E, HIST1H4F, HIST1H4C, HIST1H4D, HIST1H4I, HIST1H4J, HIST1H4H, HIST2H3A, HIST1H2BB, HIST1H3J, HIST1H2BC, HIST1H1E, HIST1H2BE, HIST1H1C, HIST1H2BF, HIST1H1B, HIST1H2BG, HIST2H3C, HIST2H3D, HIST1H3A, HIST1H2AI, HIST1H3B, HIST1H2AH, HIST1H3C, HIST1H2AK, HIST1H3D, HIST1H3E, HIST1H2AJ, HIST1H2AM, HIST1H3F, HIST1H2AL, HIST1H3G, HIST1H3H, HIST1H3I | 12.42 | 7.86E-11 |
| GO:0006323 | DNA packaging | 14 | HIST1H2AB, HIST4H4, HIST1H4L, HIST1H4K, HIST1H2AG, HIST1H2AD, HIST1H2AE, HIST2H4A, HIST2H4B, HIST1H2BO, HIST2H2AB, HIST1H2BM, HIST1H2BN, HIST1H4A, HIST1H4B, HIST1H2BI, HIST1H4E, HIST1H4F, HIST1H4C, HIST1H4D, HIST1H4I, HIST1H4J, HIST1H4H, HIST2H3A, HIST1H2BB, HIST1H3J, HIST1H2BC, HIST1H1E, HIST1H2BE, HIST1H1C, HIST1H2BF, HIST1H1B, HIST1H2BG, HIST2H3C, HIST2H3D, HIST1H3A, HIST1H2AI, HIST1H3B, HIST1H2AH, HIST1H3C, HIST1H2AK, HIST1H3D, HIST1H3E, HIST1H2AJ, HIST1H2AM, HIST1H3F, HIST1H2AL, HIST1H3G, HIST1H3H, HIST1H3I | 9.87 | 1.46E-09 |
| GO:0006333 | chromatin assembly or disassembly | 14 | HIST1H2AB, HIST4H4, HIST1H4L, HIST1H4K, HIST1H2AG, HIST1H2AD, HIST1H2AE, HIST2H4A, HIST2H4B, HIST1H2BO, HIST2H2AB, HIST1H2BM, HIST1H2BN, HIST1H4A, HIST1H4B, HIST1H2BI, HIST1H4E, HIST1H4F, HIST1H4C, HIST1H4D, HIST1H4I, HIST1H4J, HIST1H4H, HIST2H3A, HIST1H2BB, HIST1H3J, HIST1H2BC, HIST1H1E, HIST1H2BE, HIST1H1C, HIST1H2BF, HIST1H1B, HIST1H2BG, HIST2H3C, HIST2H3D, HIST1H3A, HIST1H2AI, HIST1H3B, HIST1H2AH, HIST1H3C, HIST1H2AK, HIST1H3D, HIST1H3E, HIST1H2AJ, HIST1H2AM, HIST1H3F, HIST1H2AL, HIST1H3G, HIST1H3H, HIST1H3I | 9.09 | 4.05E-09 |
| GO:0034622 | cellular macromolecular complex assembly | 19 | HIST4H4, TUBB2A, HIST2H4A, HIST2H4B, HIST1H2BO, HIST2H2AB, HIST1H2BM, HIST1H2BN, ANG, HIST1H2BI, TUBA1A, HIST1H1E, HIST1H1C, HIST1H1B, HIST1H2AB, HIST1H4L, HIST1H4K, HIST1H2AG, HIST1H2AD, HIST1H2AE, CALR, HIST1H4A, HIST1H4B, HIST1H4E, HIST1H4F, HIST1H4C, HIST1H4D, HIST1H4I, NEFL, HIST1H4J, HIST1H4H, HIST2H3A, HIST1H2BB, HIST1H3J, HIST1H2BC, HIST1H2BE, HIST1H2BF, HIST1H2BG, HIST2H3C, HIST2H3D, HIST1H3A, HIST1H2AI, HIST1H3B, HIST1H2AH, HIST1H3C, HIST1H2AK, HIST1H3D, HIST1H2AJ, HIST1H3E, HIST1H2AM, HIST1H3F, HIST1H2AL, HIST1H3G, HIST1H3H, HIST1H3I | 4.93 | 5.30E-08 |
| GO:0034621 | cellular macromolecular complex subunit organization | 19 | HIST4H4, TUBB2A, HIST2H4A, HIST2H4B, HIST1H2BO, HIST2H2AB, HIST1H2BM, HIST1H2BN, ANG, HIST1H2BI, TUBA1A, HIST1H1E, HIST1H1C, HIST1H1B, HIST1H2AB, HIST1H4L, HIST1H4K, HIST1H2AG, HIST1H2AD, HIST1H2AE, CALR, HIST1H4A, HIST1H4B, HIST1H4E, HIST1H4F, HIST1H4C, HIST1H4D, HIST1H4I, NEFL, HIST1H4J, HIST1H4H, HIST2H3A, HIST1H2BB, HIST1H3J, HIST1H2BC, HIST1H2BE, HIST1H2BF, HIST1H2BG, HIST2H3C, HIST2H3D, HIST1H3A, HIST1H2AI, HIST1H3B, HIST1H2AH, HIST1H3C, HIST1H2AK, HIST1H3D, HIST1H2AJ, HIST1H3E, HIST1H2AM, HIST1H3F, HIST1H2AL, HIST1H3G, HIST1H3H, HIST1H3I | 4.39 | 3.00E-07 |
| GO:0065003 | macromolecular complex assembly | 24 | HIST4H4, TUBB2A, SKAP2, HIST2H4A, HIST2H4B, HIST1H2BO, HIST2H2AB, HIST1H2BM, HIST1H2BN, ANG, HIST1H2BI, TUBA1A, HIST1H1E, HIST1H1C, HIST1H1B, JUP, RRM2, HIST1H2AB, HIST1H4L, HIST1H4K, HIST1H2AG, HIST1H2AD, HIST1H2AE, CALR, HIST1H4A, HIST1H4B, C1QTNF1, HIST1H4E, HIST1H4F, HIST1H4C, HIST1H4D, HIST1H4I, NEFL, HIST1H4J, HIST1H4H, HIST2H3A, HIST1H2BB, HIST1H3J, HIST1H2BC, HIST1H2BE, HIST1H2BF, HIST1H2BG, HIST2H3C, SOD2, HIST2H3D, HIST1H3A, HIST1H2AI, HIST1H3B, HIST1H2AH, HIST1H3C, HIST1H2AK, HIST1H3D, HIST1H2AJ, HIST1H3E, HIST1H2AM, HIST1H3F, HIST1H2AL, HIST1H3G, HIST1H3H, HIST1H3I | 2.98 | 4.77E-06 |
| GO:0043933 | macromolecular complex subunit organization | 24 | HIST4H4, TUBB2A, SKAP2, HIST2H4A, HIST2H4B, HIST1H2BO, HIST2H2AB, HIST1H2BM, HIST1H2BN, ANG, HIST1H2BI, TUBA1A, HIST1H1E, HIST1H1C, HIST1H1B, JUP, RRM2, HIST1H2AB, HIST1H4L, HIST1H4K, HIST1H2AG, HIST1H2AD, HIST1H2AE, CALR, HIST1H4A, HIST1H4B, C1QTNF1, HIST1H4E, HIST1H4F, HIST1H4C, HIST1H4D, HIST1H4I, NEFL, HIST1H4J, HIST1H4H, HIST2H3A, HIST1H2BB, HIST1H3J, HIST1H2BC, HIST1H2BE, HIST1H2BF, HIST1H2BG, HIST2H3C, SOD2, HIST2H3D, HIST1H3A, HIST1H2AI, HIST1H3B, HIST1H2AH, HIST1H3C, HIST1H2AK, HIST1H3D, HIST1H2AJ, HIST1H3E, HIST1H2AM, HIST1H3F, HIST1H2AL, HIST1H3G, HIST1H3H, HIST1H3I | 2.79 | 1.39E-05 |
| GO:0006325 | chromatin organization | 14 | HIST1H2AB, HIST4H4, HIST1H4L, HIST1H4K, HIST1H2AG, HIST1H2AD, HIST1H2AE, HIST2H4A, HIST2H4B, HIST1H2BO, HIST2H2AB, HIST1H2BM, HIST1H2BN, HIST1H4A, HIST1H4B, HIST1H2BI, HIST1H4E, HIST1H4F, HIST1H4C, HIST1H4D, HIST1H4I, HIST1H4J, HIST1H4H, HIST2H3A, HIST1H2BB, HIST1H3J, HIST1H2BC, HIST1H1E, HIST1H2BE, HIST1H1C, HIST1H2BF, HIST1H1B, HIST1H2BG, HIST2H3C, HIST2H3D, HIST1H3A, HIST1H2AI, HIST1H3B, HIST1H2AH, HIST1H3C, HIST1H2AK, HIST1H3D, HIST1H3E, HIST1H2AJ, HIST1H2AM, HIST1H3F, HIST1H2AL, HIST1H3G, HIST1H3H, HIST1H3I | 3.06 | 6.61E-04 |
| GO:0051276 | chromosome organization | 14 | HIST1H2AB, HIST4H4, HIST1H4L, HIST1H4K, HIST1H2AG, HIST1H2AD, HIST1H2AE, HIST2H4A, HIST2H4B, HIST1H2BO, HIST2H2AB, HIST1H2BM, HIST1H2BN, HIST1H4A, HIST1H4B, HIST1H2BI, HIST1H4E, HIST1H4F, HIST1H4C, HIST1H4D, HIST1H4I, HIST1H4J, HIST1H4H, HIST2H3A, HIST1H2BB, HIST1H3J, HIST1H2BC, HIST1H1E, HIST1H2BE, HIST1H1C, HIST1H2BF, HIST1H1B, HIST1H2BG, HIST2H3C, HIST2H3D, HIST1H3A, HIST1H2AI, HIST1H3B, HIST1H2AH, HIST1H3C, HIST1H2AK, HIST1H3D, HIST1H3E, HIST1H2AJ, HIST1H2AM, HIST1H3F, HIST1H2AL, HIST1H3G, HIST1H3H, HIST1H3I | 2.38 | 5.86E-03 |
| **Annotation Cluster 2 Enrichment Score: 5.01** | | | | | |
| GO:0030199 | collagen fibril organization | 8 | COL14A1, COL3A1, COL1A2, COL12A1, COL1A1, COL5A2, COL11A1, COL5A1 | 22.76 | 4.05E-08 |
| GO:0030198 | extracellular matrix organization | 12 | CSGALNACT1, COL14A1, ELN, COL3A1, COL1A2, COL12A1, COL1A1, VMO1, COL5A2, COL11A1, COL5A1, EMILIN1 | 9.52 | 4.70E-08 |
| GO:0043062 | extracellular structure organization | 12 | CSGALNACT1, COL14A1, ELN, COL3A1, COL1A2, COL12A1, COL1A1, VMO1, COL5A2, COL11A1, COL5A1, EMILIN1 | 6.07 | 4.39E-06 |
| GO:0043588 | skin development | 6 | COL3A1, COL1A2, COL1A1, COL5A2, COL5A1, DHCR24 | 17.07 | 2.24E-05 |
| GO:0008544 | epidermis development | 9 | LCE5A, CRABP2, KRT14, COL3A1, COL1A2, COL1A1, COL5A2, COL5A1, DHCR24 | 4.03 | 1.73E-03 |
| GO:0007398 | ectoderm development | 9 | LCE5A, CRABP2, KRT14, COL3A1, COL1A2, COL1A1, COL5A2, COL5A1, DHCR24 | 3.73 | 2.83E-03 |
| **Annotation Cluster 3 Enrichment Score: 3.57** | | | | | |
| GO:0030199 | collagen fibril organization | 8 | COL14A1, COL3A1, COL1A2, COL12A1, COL1A1, COL5A2, COL11A1, COL5A1 | 22.76 | 4.05E-08 |
| GO:0043588 | skin development | 6 | COL3A1, COL1A2, COL1A1, COL5A2, COL5A1, DHCR24 | 17.07 | 2.24E-05 |
| GO:0032963 | collagen metabolic process | 5 | COL3A1, COL1A1, MMP13, COL5A1, MMP1 | 14.73 | 3.32E-04 |
| GO:0044259 | multicellular organismal macromolecule metabolic process | 5 | COL3A1, COL1A1, MMP13, COL5A1, MMP1 | 13.30 | 4.96E-04 |
| GO:0044236 | multicellular organismal metabolic process | 5 | COL3A1, COL1A1, MMP13, COL5A1, MMP1 | 11.15 | 9.84E-04 |
| GO:0032964 | collagen biosynthetic process | 3 | COL3A1, COL1A1, COL5A1 | 49.49 | 0.001409 |
| GO:0009628 | response to abiotic stimulus | 6 | KRT14, COL3A1, COL1A1, COL11A1, MMP13, SOD2 | 1.34 | 0.456955 |
| **Annotation Cluster 4 Enrichment Score: 3.00** | | | | | |
| GO:0001568 | blood vessel development | 13 | PLAT, ANG, DHCR7, COL3A1, ITGA7, COL1A2, ZC3H12A, RHOB, COL1A1, CXCL12, COL5A1, PLAU, THY1 | 4.38 | 4.13E-05 |
| GO:0001944 | vasculature development | 13 | PLAT, ANG, DHCR7, COL3A1, ITGA7, COL1A2, ZC3H12A, RHOB, COL1A1, CXCL12, COL5A1, PLAU, THY1 | 4.27 | 5.22E-05 |
| GO:0048514 | blood vessel morphogenesis | 8 | PLAT, ANG, ITGA7, ZC3H12A, RHOB, CXCL12, PLAU, THY1 | 3.13 | 0.014027 |
| GO:0001525 | angiogenesis | 6 | ANG, ZC3H12A, RHOB, CXCL12, PLAU, THY1 | 3.34 | 3.35E-02 |
| **Annotation Cluster 5 Enrichment Score: 2.81** | | | | | |
| GO:0008610 | lipid biosynthetic process | 16 | EBP, MVD, FADS1, SCD, FADS2, ACLY, LPCAT3, PLAUR, ANG, SQLE, CH25H, DHCR7, FASN, FABP3, DHCR24, PC | 4.09 | 8.36E-06 |
| GO:0016126 | sterol biosynthetic process | 6 | EBP, MVD, SQLE, DHCR7, CH25H, DHCR24 | 14.14 | 5.79E-05 |
| GO:0006694 | steroid biosynthetic process | 6 | EBP, MVD, SQLE, DHCR7, CH25H, DHCR24 | 5.82 | 3.61E-03 |
| GO:0006695 | cholesterol biosynthetic process | 4 | EBP, MVD, DHCR7, DHCR24 | 12.69 | 3.64E-03 |
| GO:0008203 | cholesterol metabolic process | 6 | EBP, MVD, SQLE, DHCR7, CH25H, DHCR24 | 5.38 | 5.06E-03 |
| GO:0016125 | sterol metabolic process | 6 | EBP, MVD, SQLE, DHCR7, CH25H, DHCR24 | 4.90 | 0.007476 |
| GO:0008202 | steroid metabolic process | 6 | EBP, MVD, SQLE, DHCR7, CH25H, DHCR24 | 2.45 | 0.096138 |
| **Annotation Cluster 6 Enrichment Score: 1.62** | | | | | |
| GO:0009611 | response to wounding | 14 | PLAT, C3, COL3A1, CCL7, COL5A1, SOD2, PLAUR, ITGA5, SAA1, VCAN, REG3G, LBP, NEFL, PLAU | 2.18 | 0.011839 |
| GO:0016477 | cell migration | 9 | PLAT, SAA1, ITGA5, ANG, VCAN, NTN1, CXCL12, COL5A1, PLAU | 2.69 | 0.018702 |
| GO:0042060 | wound healing | 7 | PLAT, SAA1, ITGA5, COL3A1, COL5A1, PLAU, PLAUR | 3.02 | 2.80E-02 |
| GO:0006928 | cell motion | 12 | PLAT, SAA1, ITGA5, ANG, VCAN, GAS1, NTN1, CXCL12, COL5A1, PLAU, SLIT3, PLAUR | 2.08 | 0.028828 |
| GO:0048870 | cell motility | 9 | PLAT, SAA1, ITGA5, ANG, VCAN, NTN1, CXCL12, COL5A1, PLAU | 2.42 | 0.032545 |
| GO:0051674 | localization of cell | 9 | PLAT, SAA1, ITGA5, ANG, VCAN, NTN1, CXCL12, COL5A1, PLAU | 2.42 | 0.032545 |

| **Table S15 Functional annotation clustering – Gene Ontology Terms for Biological Process: MSC-17 vs. MSC-γ (downregulated genes)** | | | | | |
| --- | --- | --- | --- | --- | --- |
| **GO TERM ID** | **Term** | **Count** | **Genes** | **Fold Enrichment** | **P-Value** |
| **Annotation Cluster 1 Enrichment Score: 9.11** | | | | | |
| GO:0019882 | antigen processing and presentation | 31 | HLA-DQB1, HLA-DRB1, HLA-DRB3, IFI30, HLA-DMB, HLA-DMA, CD74, B2M, TAPBP, TAP2, HLA-DRB4, ERAP1, ERAP2, HLA-DPB1, MR1, HLA-DOA, ICAM1, HLA-L, HLA-A, HLA-C, HLA-B, HLA-E, HLA-DQA2, PSMB8, HLA-G, HLA-DQA1, HLA-F, PSMB9, PSME1, ULBP1, HLA-DPA1, TAPBPL, HLA-DRA | 7.41 | 1.55E-18 |
| GO:0048002 | antigen processing and presentation of peptide antigen | 17 | HLA-A, IFI30, HLA-C, HLA-B, HLA-E, HLA-DMA, HLA-G, CD74, B2M, HLA-F, TAPBP, TAP2, ERAP1, ERAP2, MR1, TAPBPL, HLA-DOA, HLA-DRA | 12.04 | 2.47E-14 |
| GO:0002474 | antigen processing and presentation of peptide antigen via MHC class I | 12 | TAP2, HLA-A, ERAP1, HLA-C, ERAP2, HLA-B, MR1, HLA-E, TAPBPL, HLA-G, TAPBP, B2M, HLA-F | 14.00 | 4.56E-11 |
| GO:0019883 | antigen processing and presentation of endogenous antigen | 6 | TAP2, ERAP1, ERAP2, TAPBPL, CD74, TAPBP | 14.88 | 1.57E-05 |
| GO:0002483 | antigen processing and presentation of endogenous peptide antigen | 5 | TAP2, ERAP1, ERAP2, TAPBPL, TAPBP | 16.53 | 8.80E-05 |
| GO:0019885 | antigen processing and presentation of endogenous peptide antigen via MHC class I | 5 | TAP2, ERAP1, ERAP2, TAPBPL, TAPBP | 16.53 | 8.80E-05 |
| **Annotation Cluster 2 Enrichment Score: 5.61** | | | | | |
| GO:0002504 | ntigen processing and presentation of peptide or polysaccharide antigen via MHC class II | 13 | HLA-DQB1, HLA-DRB1, HLA-DRB3, IFI30, HLA-DMB, HLA-DMA, HLA-DQA2, HLA-DQA1, CD74, HLA-DRB4, HLA-DPA1, HLA-DPB1, HLA-DOA, HLA-DRA | 7.81 | 3.22E-08 |
| GO:0019884 | antigen processing and presentation of exogenous antigen | 9 | PSME1, TAP2, IFI30, HLA-DOA, HLA-DMA, CD74, TAPBP, HLA-DRA, B2M | 12.75 | 9.08E-08 |
| GO:0002478 | antigen processing and presentation of exogenous peptide antigen | 8 | TAP2, IFI30, HLA-DOA, HLA-DMA, CD74, TAPBP, HLA-DRA, B2M | 14.43 | 2.20E-07 |
| GO:0002495 | antigen processing and presentation of peptide antigen via MHC class II | 5 | IFI30, HLA-DOA, HLA-DMA, CD74, HLA-DRA | 12.40 | 3.79E-04 |
| GO:0019886 | antigen processing and presentation of exogenous peptide antigen via MHC class II | 5 | IFI30, HLA-DOA, HLA-DMA, CD74, HLA-DRA | 12.40 | 3.79E-04 |
| **Annotation Cluster 3 Enrichment Score: 4.19** | | | | | |
| GO:0034976 | response to endoplasmic reticulum stress | 12 | ATF6, HERPUD1, ATF4, AARS, ERN1, AMFR, NFE2L2, EIF2AK2, PPP1R15B, FAM129A, PPP1R15A, DDIT3 | 7.00 | 4.80E-07 |
| GO:0030968 | endoplasmic reticulum unfolded protein response | 9 | ATF6, HERPUD1, AARS, ERN1, AMFR, NFE2L2, EIF2AK2, PPP1R15A, DDIT3 | 8.50 | 4.49E-06 |
| GO:0034620 | cellular response to unfolded protein | 9 | ATF6, HERPUD1, AARS, ERN1, AMFR, NFE2L2, EIF2AK2, PPP1R15A, DDIT3 | 8.50 | 4.49E-06 |
| GO:0006984 | ER-nuclear signaling pathway | 10 | ATF6, HERPUD1, AARS, ERN1, AMFR, NFE2L2, EIF2AK2, PPP1R15B, PPP1R15A, DDIT3 | 5.67 | 4.27E-05 |
| GO:0006986 | response to unfolded protein | 10 | ATF6, HERPUD1, CFB, AARS, ERN1, AMFR, NFE2L2, EIF2AK2, PPP1R15A, DDIT3 | 2.79 | 0.00899 |
| GO:0051789 | response to protein stimulus | 12 | ATF6, HERPUD1, CFB, BCL2, MAP1B, AARS, ERN1, AMFR, NFE2L2, EIF2AK2, PPP1R15A, DDIT3 | 2.22 | 0.01874 |
| **Annotation Cluster 4 Enrichment Score: 3.19** | | | | | |
| GO:0045087 | innate immune response | 23 | CIITA, IL18R1, IFIH1, SP100, C4A, CFB, C4B, CEBPG, CLU, TLR3, CALCOCO2, SAMHD1, MALT1, SERPING1, C1R, TLR4, C1S, GCH1, DDX58, APOL1, C1RL, C2, DHX58 | 3.31 | 1.43E-06 |
| GO:0002252 | immune effector process | 21 | ICAM1, C4A, CFB, C4B, CEBPG, CLU, RSAD2, SAMHD1, SERPING1, C1R, C1S, HLA-DMA, CD74, TNFSF13B, BCL2, C1RL, PMS2, C2, PMS2CL, PTX3, HLA-DRA | 3.11 | 1.19E-05 |
| GO:0002460 | adaptive immune response based on somatic recombination of immune receptors built from immunoglobulin superfamily domains | 15 | ICAM1, C4A, C4B, CLU, SERPING1, C1R, TLR4, C1S, HLA-DMA, CD74, IL18BP, VEGFA, C1RL, C2, HLA-DRA | 3.86 | 2.58E-05 |
| GO:0002250 | adaptive immune response | 15 | ICAM1, C4A, C4B, CLU, SERPING1, C1R, TLR4, C1S, HLA-DMA, CD74, IL18BP, VEGFA, C1RL, C2, HLA-DRA | 3.86 | 2.58E-05 |
| GO:0050778 | positive regulation of immune response | 21 | C4A, C4B, CFB, CLU, TLR3, MALT1, SERPING1, C1R, TLR4, IDO1, C1S, IL15, POLR3C, HLA-DMA, B2M, TNFSF13B, TAP2, C1RL, RIPK2, C2, HLA-DRA | 2.87 | 3.86E-05 |
| GO:0006952 | defense response | 55 | A2M, TLR3, TLR4, IL15, CXCL11, CXCL10, TAPBP, CD97, HCP5, MX1, FOSL1, MX2, CIITA, SP100, C4A, C4B, SCUBE1, HLA-C, SERPING1, HLA-B, HLA-G, RIPK2, NGF, HLA-DRA, IFIH1, NMI, CLU, CXCL9, CALCOCO2, RSAD2, C1R, IL32, C1S, CD74, GCH1, LGALS3BP, TAP2, BCL2, TAP1, C2, PTX3, DHX58, LY75, IL18R1, CFB, CEBPG, SAMHD1, MALT1, IDO1, APOL2, DDX58, APOL3, CCL13, APOL1, NUPR1, C1RL, CD302 | 1.77 | 4.50E-05 |
| GO:0048584 | positive regulation of response to stimulus | 28 | OSMR, CLU, TLR3, TLR4, C1R, IL15, C1S, HLA-DMA, B2M, EDNRA, TAP2, C2, C4A, C4B, CFB, CEBPG, EDA2R, SERPING1, MALT1, IDO1, POLR3C, TNFSF13B, HIPK2, VEGFA, C1RL, RIPK2, JAK2, HLA-DRA | 2.35 | 5.76E-05 |
| GO:0002684 | positive regulation of immune system process | 28 | CLU, TLR3, C1R, TLR4, IL15, C1S, HLA-DMA, CD74, B2M, CD47, TAP2, BCL6, C2, ICAM1, C4A, CFB, C4B, MALT1, SERPING1, IDO1, POLR3C, NCK2, CBLB, TNFSF13B, VEGFA, C1RL, RIPK2, HLA-DRA | 2.33 | 6.65E-05 |
| GO:0002449 | lymphocyte mediated immunity | 13 | ICAM1, C4A, C4B, CEBPG, CLU, C1RL, C1R, SERPING1, C1S, C2, HLA-DMA, CD74, HLA-DRA | 3.68 | 1.74E-04 |
| GO:0016064 | immunoglobulin mediated immune response | 11 | C4A, C4B, CLU, C1RL, C1R, SERPING1, C1S, C2, HLA-DMA, CD74, HLA-DRA | 4.04 | 3.13E-04 |
| GO:0019724 | B cell mediated immunity | 11 | C4A, C4B, CLU, C1RL, C1R, SERPING1, C1S, C2, HLA-DMA, CD74, HLA-DRA | 3.90 | 4.25E-04 |
| GO:0006958 | complement activation, classical pathway | 8 | C4A, C4B, CLU, C1RL, C1R, SERPING1, C1S, C2 | 5.47 | 4.69E-04 |
| GO:0002455 | humoral immune response mediated by circulating immunoglobulin | 8 | C4A, C4B, CLU, C1RL, C1R, SERPING1, C1S, C2 | 5.12 | 7.24E-04 |
| GO:0006956 | complement activation | 9 | C4A, CFB, C4B, CLU, C1RL, C1R, SERPING1, C1S, C2 | 4.25 | 0.001019 |
| GO:0002541 | activation of plasma proteins involved in acute inflammatory response | 9 | C4A, CFB, C4B, CLU, C1RL, C1R, SERPING1, C1S, C2 | 4.15 | 0.001198 |
| GO:0002443 | leukocyte mediated immunity | 13 | ICAM1, C4A, C4B, CEBPG, CLU, C1RL, C1R, SERPING1, C1S, C2, HLA-DMA, CD74, HLA-DRA | 3.00 | 0.0012 |
| GO:0002526 | acute inflammatory response | 14 | A2M, C4A, C4B, CFB, CLU, TLR4, SERPING1, C1R, IDO1, C1S, APOL2, NUPR1, C1RL, C2 | 2.83 | 0.00123 |
| GO:0006959 | humoral immune response | 12 | PSMB10, BST2, C4A, CFB, C4B, BCL2, CLU, C1RL, C1R, SERPING1, C1S, C2 | 3.01 | 0.001929 |
| GO:0002253 | activation of immune response | 13 | C4A, C4B, CFB, CLU, TLR3, MALT1, TLR4, SERPING1, C1R, C1S, C1RL, RIPK2, C2 | 2.74 | 0.002601 |
| GO:0006954 | inflammatory response | 29 | LY75, CIITA, A2M, NMI, C4A, C4B, CFB, SCUBE1, CLU, CXCL9, TLR3, TLR4, C1R, SERPING1, IDO1, C1S, IL15, CXCL11, CXCL10, CD97, APOL2, APOL3, CCL13, NUPR1, C1RL, RIPK2, C2, PTX3, CD302, NGF | 1.77 | 0.003941 |
| GO:0051605 | protein maturation by peptide bond cleavage | 10 | C4A, CFB, C4B, CLU, C1RL, C1R, SERPING1, C1S, C2, NGF | 2.31 | 0.028677 |
| GO:0009611 | response to wounding | 37 | F2RL2, A2M, NMI, HPS5, CLU, CXCL9, TLR3, TLR4, C1R, IL15, C1S, CXCL11, MDK, CXCL10, CD97, BCL2, C2, PTX3, FGF2, CIITA, LY75, C4A, C4B, CFB, SCUBE1, MAP1B, SERPING1, IDO1, APOL2, APOL3, PLSCR1, CCL13, NUPR1, C1RL, RIPK2, JAK2, CD302, NGF | 1.38 | 0.041126 |
| GO:0016485 | protein processing | 11 | C2CD3, C4A, CFB, C4B, CLU, C1RL, C1R, SERPING1, C1S, C2, NGF | 1.95 | 0.055622 |
| GO:0051604 | protein maturation | 11 | C2CD3, C4A, CFB, C4B, CLU, C1RL, C1R, SERPING1, C1S, C2, NGF | 1.79 | 0.087608 |
| GO:0006957 | complement activation, alternative pathway | 3 | C4A, CFB, C2 | 3.97 | 0.172728 |
| Annotation Cluster 5 | Enrichment Score: 3.03 | |  |  |  |
| GO:0043038 | amino acid activation | 12 | IARS, WARS, TARS, YARS, CARS, ZNFX1, SARS, AARS, GARS, SARS2, KARS, MARS | 5.17 | 1.29E-05 |
| GO:0043039 | tRNA aminoacylation | 12 | IARS, WARS, TARS, YARS, CARS, ZNFX1, SARS, AARS, GARS, SARS2, KARS, MARS | 5.17 | 1.29E-05 |
| GO:0006418 | tRNA aminoacylation for protein translation | 12 | IARS, WARS, TARS, YARS, CARS, ZNFX1, SARS, AARS, GARS, SARS2, KARS, MARS | 5.17 | 1.29E-05 |
| GO:0006399 | tRNA metabolic process | 13 | IARS, WARS, TARS, YARS, CARS, ZNFX1, SARS, AARS, GARS, KIAA0391, SARS2, KARS, MARS | 2.19 | 0.015654 |
| GO:0006412 | translation | 24 | EGFR, YARS, CARS, ZNFX1, SARS, AARS, GARS, RPL37, RPS27L, EIF1B, RPLP0P2, MRRF, SARS2, KARS, QRSL1, IARS, WARS, TARS, CYLD, RPL23AP32, RPL26L1, EIF2AK2, EIF5A2, MARS | 1.44 | 0.073696 |
| GO:0034660 | ncRNA metabolic process | 15 | CARS, EXOSC8, YARS, EXOSC6, ZNFX1, SARS, AARS, GARS, KARS, SARS2, IARS, WARS, TARS, KIAA0391, MARS | 1.29 | 0.269742 |
| Annotation Cluster 6 | Enrichment Score: 2.91 | |  |  |  |
| GO:0043067 | regulation of programmed cell death | 66 | RTN4, SH3RF1, HTATIP2, PTGS2, CBX4, TLR4, RPS27L, PMAIP1, CASP8, NQO1, MX1, FGF2, FOSL1, KCNMA1, EGFR, ARHGEF2, AIFM2, DFFA, ARHGEF6, AARS, ADNP, ARHGEF12, STK4, DDIT3, DAPK1, JMY, TNFRSF10B, TNFSF13B, IGF2R, HIPK2, VEGFA, ERN1, RIPK2, FOXC1, NAIP, PRNP, NGF, CAMK1D, IFIH1, CLU, ASNS, GCLM, CD74, GCH1, SQSTM1, SOS1, BCL2, BCL6, HIP1, HSPA9, TXNIP, CFLAR, HERPUD1, ABR, SMAD6, CEBPG, MALT1, IDO1, STRADB, STAT1, ATF5, TNFSF10, NUPR1, JAK2, IFI6, TP53INP1 | 1.61 | 1.25E-04 |
| GO:0010941 | regulation of cell death | 66 | RTN4, SH3RF1, HTATIP2, PTGS2, CBX4, TLR4, RPS27L, PMAIP1, CASP8, NQO1, MX1, FGF2, FOSL1, KCNMA1, EGFR, ARHGEF2, AIFM2, DFFA, ARHGEF6, AARS, ADNP, ARHGEF12, STK4, DDIT3, DAPK1, JMY, TNFRSF10B, TNFSF13B, IGF2R, HIPK2, VEGFA, ERN1, RIPK2, FOXC1, NAIP, PRNP, NGF, CAMK1D, IFIH1, CLU, ASNS, GCLM, CD74, GCH1, SQSTM1, SOS1, BCL2, BCL6, HIP1, HSPA9, TXNIP, CFLAR, HERPUD1, ABR, SMAD6, CEBPG, MALT1, IDO1, STRADB, STAT1, ATF5, TNFSF10, NUPR1, JAK2, IFI6, TP53INP1 | 1.61 | 1.40E-04 |
| GO:0042981 | regulation of apoptosis | 65 | RTN4, SH3RF1, HTATIP2, PTGS2, CBX4, TLR4, RPS27L, PMAIP1, CASP8, NQO1, MX1, FOSL1, KCNMA1, EGFR, ARHGEF2, AIFM2, DFFA, ARHGEF6, AARS, ADNP, ARHGEF12, STK4, DDIT3, DAPK1, JMY, TNFRSF10B, TNFSF13B, IGF2R, HIPK2, VEGFA, ERN1, RIPK2, FOXC1, NAIP, PRNP, NGF, CAMK1D, IFIH1, CLU, ASNS, GCLM, CD74, GCH1, SQSTM1, SOS1, BCL2, BCL6, HIP1, HSPA9, TXNIP, CFLAR, HERPUD1, ABR, SMAD6, CEBPG, MALT1, IDO1, STRADB, STAT1, ATF5, TNFSF10, NUPR1, JAK2, IFI6, TP53INP1 | 1.60 | 1.69E-04 |
| GO:0008219 | cell death | 59 | RTN4, HTATIP2, PMAIP1, SETX, UNC5B, TPP1, CASP8, FGF2, SGPL1, YARS, ARHGEF2, AIFM2, DFFA, ARHGEF6, OPTN, ARHGEF12, STK4, AHR, DDIT4, DAPK1, EP300, TNFRSF10B, HIPK2, MGEA5, ERN1, RIPK2, NAIP, MDM4, SIAH2, EIF2AK2, GADD45A, PPP1R15A, NGF, CLU, TRIB3, RRAGC, SQSTM1, TRIM69, SOS1, BCL2, XAF1, HIP1, RNF144B, CFLAR, ABR, KLF11, GARS, FIG4, STAT1, ITPR1, ATXN3, TNFSF10, APOL1, SULF1, JAK2, SPG11, DRAM1, IFI6, TP53INP1 | 1.63 | 2.40E-04 |
| GO:0043065 | positive regulation of apoptosis | 40 | SH3RF1, HTATIP2, PTGS2, RPS27L, TLR4, PMAIP1, GCH1, SQSTM1, BCL2, SOS1, CASP8, BCL6, MX1, NQO1, FOSL1, HIP1, TXNIP, KCNMA1, CFLAR, ARHGEF2, ABR, AIFM2, DFFA, CEBPG, ARHGEF6, ARHGEF12, STAT1, STK4, DDIT3, DAPK1, JMY, TNFSF10, TNFRSF10B, NUPR1, HIPK2, ERN1, RIPK2, JAK2, NGF, TP53INP1 | 1.85 | 2.69E-04 |
| GO:0016265 | death | 59 | RTN4, HTATIP2, PMAIP1, SETX, UNC5B, TPP1, CASP8, FGF2, SGPL1, YARS, ARHGEF2, AIFM2, DFFA, ARHGEF6, OPTN, ARHGEF12, STK4, AHR, DDIT4, DAPK1, EP300, TNFRSF10B, HIPK2, MGEA5, ERN1, RIPK2, NAIP, MDM4, SIAH2, EIF2AK2, GADD45A, PPP1R15A, NGF, CLU, TRIB3, RRAGC, SQSTM1, TRIM69, SOS1, BCL2, XAF1, HIP1, RNF144B, CFLAR, ABR, KLF11, GARS, FIG4, STAT1, ITPR1, ATXN3, TNFSF10, APOL1, SULF1, JAK2, SPG11, DRAM1, IFI6, TP53INP1 | 1.62 | 2.83E-04 |
| GO:0043068 | positive regulation of programmed cell death | 40 | SH3RF1, HTATIP2, PTGS2, RPS27L, TLR4, PMAIP1, GCH1, SQSTM1, BCL2, SOS1, CASP8, BCL6, MX1, NQO1, FOSL1, HIP1, TXNIP, KCNMA1, CFLAR, ARHGEF2, ABR, AIFM2, DFFA, CEBPG, ARHGEF6, ARHGEF12, STAT1, STK4, DDIT3, DAPK1, JMY, TNFSF10, TNFRSF10B, NUPR1, HIPK2, ERN1, RIPK2, JAK2, NGF, TP53INP1 | 1.83 | 3.09E-04 |
| GO:0010942 | positive regulation of cell death | 40 | SH3RF1, HTATIP2, PTGS2, RPS27L, TLR4, PMAIP1, GCH1, SQSTM1, BCL2, SOS1, CASP8, BCL6, MX1, NQO1, FOSL1, HIP1, TXNIP, KCNMA1, CFLAR, ARHGEF2, ABR, AIFM2, DFFA, CEBPG, ARHGEF6, ARHGEF12, STAT1, STK4, DDIT3, DAPK1, JMY, TNFSF10, TNFRSF10B, NUPR1, HIPK2, ERN1, RIPK2, JAK2, NGF, TP53INP1 | 1.82 | 3.40E-04 |
| GO:0006915 | apoptosis | 49 | RTN4, HTATIP2, CLU, TRIB3, PMAIP1, RRAGC, UNC5B, TRIM69, SQSTM1, SOS1, BCL2, CASP8, XAF1, FGF2, HIP1, SGPL1, RNF144B, CFLAR, ARHGEF2, YARS, ABR, AIFM2, DFFA, ARHGEF6, KLF11, ARHGEF12, STAT1, STK4, AHR, DDIT4, DAPK1, TNFSF10, EP300, TNFRSF10B, HIPK2, SULF1, ERN1, RIPK2, NAIP, JAK2, MDM4, SIAH2, EIF2AK2, DRAM1, PPP1R15A, GADD45A, IFI6, NGF, TP53INP1 | 1.61 | 0.0011 |
| GO:0012501 | programmed cell death | 49 | RTN4, HTATIP2, CLU, TRIB3, PMAIP1, RRAGC, UNC5B, TRIM69, SQSTM1, SOS1, BCL2, CASP8, XAF1, FGF2, HIP1, SGPL1, RNF144B, CFLAR, ARHGEF2, YARS, ABR, AIFM2, DFFA, ARHGEF6, KLF11, ARHGEF12, STAT1, STK4, AHR, DDIT4, DAPK1, TNFSF10, EP300, TNFRSF10B, HIPK2, SULF1, ERN1, RIPK2, NAIP, JAK2, MDM4, SIAH2, EIF2AK2, DRAM1, PPP1R15A, GADD45A, IFI6, NGF, TP53INP1 | 1.59 | 0.0014 |
| GO:0006917 | induction of apoptosis | 29 | SH3RF1, HTATIP2, RPS27L, PMAIP1, GCH1, SQSTM1, SOS1, CASP8, MX1, HIP1, CFLAR, ARHGEF2, ABR, AIFM2, DFFA, CEBPG, ARHGEF6, STAT1, ARHGEF12, JMY, DAPK1, TNFSF10, TNFRSF10B, NUPR1, HIPK2, ERN1, JAK2, NGF, TP53INP1 | 1.80 | 0.0032 |
| GO:0012502 | induction of programmed cell death | 29 | SH3RF1, HTATIP2, RPS27L, PMAIP1, GCH1, SQSTM1, SOS1, CASP8, MX1, HIP1, CFLAR, ARHGEF2, ABR, AIFM2, DFFA, CEBPG, ARHGEF6, STAT1, ARHGEF12, JMY, DAPK1, TNFSF10, TNFRSF10B, NUPR1, HIPK2, ERN1, JAK2, NGF, TP53INP1 | 1.79 | 0.0033 |
| GO:0043066 | negative regulation of apoptosis | 30 | HTATIP2, CLU, CBX4, ASNS, GCLM, CD74, SQSTM1, BCL2, BCL6, HSPA9, EGFR, CFLAR, DFFA, SMAD6, AARS, ADNP, MALT1, IDO1, STRADB, DAPK1, ATF5, TNFSF13B, HIPK2, VEGFA, RIPK2, FOXC1, NAIP, PRNP, IFI6, NGF | 1.68 | 0.0068 |
| GO:0043069 | negative regulation of programmed cell death | 30 | HTATIP2, CLU, CBX4, ASNS, GCLM, CD74, SQSTM1, BCL2, BCL6, HSPA9, EGFR, CFLAR, DFFA, SMAD6, AARS, ADNP, MALT1, IDO1, STRADB, DAPK1, ATF5, TNFSF13B, HIPK2, VEGFA, RIPK2, FOXC1, NAIP, PRNP, IFI6, NGF | 1.66 | 0.0082 |
| GO:0060548 | negative regulation of cell death | 30 | HTATIP2, CLU, CBX4, ASNS, GCLM, CD74, SQSTM1, BCL2, BCL6, HSPA9, EGFR, CFLAR, DFFA, SMAD6, AARS, ADNP, MALT1, IDO1, STRADB, DAPK1, ATF5, TNFSF13B, HIPK2, VEGFA, RIPK2, FOXC1, NAIP, PRNP, IFI6, NGF | 1.65 | 0.0085 |
| GO:0006916 | anti-apoptosis | 19 | CFLAR, HTATIP2, CLU, CBX4, MALT1, STRADB, DAPK1, ATF5, TNFSF13B, SQSTM1, BCL2, VEGFA, RIPK2, NAIP, FOXC1, PRNP, IFI6, HSPA9, NGF | 1.83 | 0.0162 |
| **Annotation Cluster 7 Enrichment Score: 2.41** | | | | | |
| GO:0002237 | response to molecule of bacterial origin | 14 | PTGS2, TAP2, SOCS1, ADH5, RIPK2, MALT1, TIMP4, TLR4, IDO1, STAT1, B2M, VLDLR, GCH1, NGF | 3.23 | 3.46E-04 |
| GO:0032496 | response to lipopolysaccharide | 11 | PTGS2, SOCS1, ADH5, RIPK2, TIMP4, TLR4, IDO1, STAT1, VLDLR, GCH1, NGF | 2.83 | 0.0051 |
| GO:0009617 | response to bacterium | 17 | PTGS2, SOCS1, ADH5, TLR3, MALT1, TIMP4, TLR4, IDO1, STAT1, B2M, GCH1, TAP2, RIPK2, ERAP1, WASL, NGF, VLDLR | 1.75 | 0.0345 |
| **Annotation Cluster 8 Enrichment Score: 2.17** | | | | | |
| GO:0043122 | regulation of I-kappaB kinase/NF-kappaB cascade | 15 | CFLAR, SECTM1, SLC44A2, BST2, TLR3, MALT1, TLR4, TRIM38, APOL3, TNFSF10, TNFRSF10B, MAP3K3, SQSTM1, CASP8, RIPK2 | 2.78 | 9.30E-04 |
| GO:0043123 | positive regulation of I-kappaB kinase/NF-kappaB cascade | 14 | CFLAR, SECTM1, SLC44A2, BST2, TLR3, TLR4, MALT1, TRIM38, APOL3, TNFSF10, TNFRSF10B, MAP3K3, CASP8, RIPK2 | 2.86 | 0.0011 |
| GO:0010740 | positive regulation of protein kinase cascade | 17 | CFLAR, SECTM1, SLC44A2, BST2, EDA2R, TLR3, MALT1, TLR4, TRIM38, APOL3, TNFSF10, TNFRSF10B, MAP3K3, HIPK2, CASP8, RIPK2, JAK2 | 2.02 | 0.0101 |
| GO:0010627 | regulation of protein kinase cascade | 22 | SECTM1, CFLAR, SH3RF1, SLC44A2, BST2, SOCS1, TLR3, EDA2R, MALT1, TLR4, TRIM38, APOL3, TNFSF10, TNFRSF10B, AIDA, MAP3K3, SQSTM1, HIPK2, CASP8, RIPK2, JAK2, NCOR1 | 1.75 | 0.0144 |
| GO:0009967 | positive regulation of signal transduction | 24 | SECTM1, CFLAR, SLC44A2, BST2, CSF1, TLR3, EDA2R, MALT1, TLR4, TRIM16, JAG1, TRIM38, APOL3, TNFSF10, TNFRSF10B, NCOA3, MAP3K3, SOS1, HIPK2, VEGFA, CASP8, RIPK2, ZRANB1, JAK2 | 1.61 | 0.0251 |
| GO:0010647 | positive regulation of cell communication | 26 | SLC44A2, PTGS2, CSF1, TLR3, TRIM16, TLR4, JAG1, MAP3K3, SOS1, CASP8, EGFR, CFLAR, SECTM1, BST2, EDA2R, MALT1, TRIM38, APOL3, TNFSF10, TNFRSF10B, NCOA3, HIPK2, VEGFA, ZRANB1, RIPK2, JAK2 | 1.57 | 0.0263 |
| **Annotation Cluster 9 Enrichment Score: 2.09** | | | | | |
| GO:0009069 | serine family amino acid metabolic process | 9 | PDPR, CTH, SEPHS2, SHMT2, PHGDH, DMGDH, PSAT1, PSPH, GCLM | 6.87 | 2.75E-05 |
| GO:0009070 | serine family amino acid biosynthetic process | 5 | CTH, SEPHS2, PHGDH, PSAT1, PSPH | 9.02 | 0.0016 |
| GO:0008652 | cellular amino acid biosynthetic process | 9 | CTH, SEPHS2, GOT1, MAT2A, ASS1, PHGDH, ASNS, PSAT1, PSPH | 3.50 | 0.0037 |
| GO:0006563 | L-serine metabolic process | 4 | SHMT2, PHGDH, PSAT1, PSPH | 9.92 | 0.0059 |
| GO:0006564 | L-serine biosynthetic process | 3 | PHGDH, PSAT1, PSPH | 19.84 | 0.0073 |
| GO:0009309 | amine biosynthetic process | 10 | CTH, SEPHS2, GOT1, MAT2A, ASS1, PHGDH, ASNS, PSAT1, PSPH, GCH1 | 2.45 | 0.0203 |
| GO:0046394 | carboxylic acid biosynthetic process | 14 | SEPHS2, ASS1, MAT2A, PTGS2, ASNS, IDO1, PSPH, CD74, CTH, GOT1, PTGDS, PHGDH, LIAS, PSAT1 | 1.79 | 0.0491 |
| GO:0016053 | organic acid biosynthetic process | 14 | SEPHS2, ASS1, MAT2A, PTGS2, ASNS, IDO1, PSPH, CD74, CTH, GOT1, PTGDS, PHGDH, LIAS, PSAT1 | 1.79 | 0.0491 |
| **Annotation Cluster 10 Enrichment Score: 2.08** | | | | | |
| GO:0051091 | positive regulation of transcription factor activity | 12 | ICAM1, SP100, EP300, NCOA3, CEBPG, TLR3, RIPK2, EDA2R, MALT1, JAK2, TLR4, JMY | 3.97 | 1.74E-04 |
| GO:0043388 | positive regulation of DNA binding | 13 | ICAM1, SP100, EP300, NCOA3, CEBPG, HIPK2, TLR3, RIPK2, EDA2R, MALT1, JAK2, TLR4, JMY | 3.68 | 1.74E-04 |
| GO:0051099 | positive regulation of binding | 13 | ICAM1, SP100, EP300, NCOA3, CEBPG, HIPK2, TLR3, RIPK2, EDA2R, MALT1, JAK2, TLR4, JMY | 3.31 | 4.92E-04 |
| GO:0051101 | regulation of DNA binding | 16 | ICAM1, SP100, THRA, CEBPG, TLR3, EDA2R, MALT1, TLR4, DDIT3, JMY, EP300, NCOA3, HIPK2, RIPK2, TNKS, JAK2 | 2.62 | 0.0011 |
| GO:0051098 | regulation of binding | 18 | ICAM1, SP100, THRA, CEBPG, TLR3, EDA2R, MALT1, TLR4, DDIT3, JMY, EP300, AIDA, NCOA3, BCL2, HIPK2, RIPK2, JAK2, TNKS | 2.33 | 0.0018 |
| GO:0051090 | regulation of transcription factor activity | 14 | ICAM1, SP100, THRA, CEBPG, EDA2R, TLR3, MALT1, TLR4, DDIT3, JMY, EP300, NCOA3, RIPK2, JAK2 | 2.70 | 0.0019 |
| GO:0046330 | positive regulation of JNK cascade | 5 | HIPK2, TLR3, RIPK2, EDA2R, TLR4 | 5.83 | 0.0090 |
| GO:0070304 | positive regulation of stress-activated protein kinase signaling pathway | 5 | HIPK2, TLR3, RIPK2, EDA2R, TLR4 | 4.72 | 0.0192 |
| GO:0051092 | positive regulation of NF-kappaB transcription factor activity | 6 | ICAM1, TLR3, RIPK2, EDA2R, MALT1, TLR4 | 2.90 | 0.0535 |
| GO:0043392 | negative regulation of DNA binding | 6 | SP100, THRA, CEBPG, TNKS, JAK2, DDIT3 | 2.33 | 0.1124 |
| GO:0051100 | negative regulation of binding | 6 | SP100, THRA, CEBPG, TNKS, JAK2, DDIT3 | 2.02 | 0.1748 |
| GO:0043410 | positive regulation of MAPKKK cascade | 5 | HIPK2, TLR3, RIPK2, EDA2R, TLR4 | 2.07 | 0.2212 |
| GO:0043433 | negative regulation of transcription factor activity | 4 | SP100, THRA, CEBPG, DDIT3 | 1.76 | 0.3966 |
| **Annotation Cluster 11 Enrichment Score: 2.06** | | | | | |
| GO:0051603 | proteolysis involved in cellular protein catabolic process | 46 | PSMB10, CBX4, UBA7, RNF217, SENP5, MYCBP2, CYLD, ISG15, SQSTM1, WWP2, MAP1LC3B, CASP8, RBCK1, ERAP1, RNF149, FBXL17, USP31, RNF144B, FBXL20, SOCS1, HERC6, UBR4, UBE2L6, MALT1, UBR2, PSMB8, PSMB9, WSB1, CBLB, TULP4, SENP1, PSMA6, PSME1, PSME2, FBXO17, PSMA4, DET1, KLHL12, MDM2, ZRANB1, FBXL7, AMFR, SIAH2, RNF19B, USP42, RNF41 | 1.52 | 0.0048 |
| GO:0044257 | cellular protein catabolic process | 46 | PSMB10, CBX4, UBA7, RNF217, SENP5, MYCBP2, CYLD, ISG15, SQSTM1, WWP2, MAP1LC3B, CASP8, RBCK1, ERAP1, RNF149, FBXL17, USP31, RNF144B, FBXL20, SOCS1, HERC6, UBR4, UBE2L6, MALT1, UBR2, PSMB8, PSMB9, WSB1, CBLB, TULP4, SENP1, PSMA6, PSME1, PSME2, FBXO17, PSMA4, DET1, KLHL12, MDM2, ZRANB1, FBXL7, AMFR, SIAH2, RNF19B, USP42, RNF41 | 1.51 | 0.0052 |
| GO:0030163 | protein catabolic process | 47 | PSMB10, CBX4, UBA7, RNF217, SENP5, MYCBP2, CYLD, ISG15, SQSTM1, WWP2, MAP1LC3B, TPP1, CASP8, RBCK1, ERAP1, RNF149, FBXL17, USP31, RNF144B, FBXL20, SOCS1, HERC6, UBR4, UBE2L6, MALT1, UBR2, PSMB8, PSMB9, WSB1, TULP4, CBLB, SENP1, PSMA6, PSME1, PSME2, FBXO17, PSMA4, DET1, KLHL12, MDM2, ZRANB1, FBXL7, AMFR, SIAH2, RNF19B, USP42, RNF41 | 1.50 | 0.0056 |
| GO:0043632 | modification-dependent macromolecule catabolic process | 44 | PSMB10, CBX4, UBA7, RNF217, SENP5, MYCBP2, CYLD, ISG15, SQSTM1, MAP1LC3B, WWP2, RBCK1, RNF149, FBXL17, USP31, RNF144B, FBXL20, SOCS1, HERC6, UBR4, UBE2L6, MALT1, UBR2, PSMB8, PSMB9, WSB1, CBLB, TULP4, SENP1, PSMA6, PSME1, PSME2, FBXO17, PSMA4, DET1, KLHL12, MDM2, ZRANB1, FBXL7, AMFR, SIAH2, RNF19B, USP42, RNF41 | 1.52 | 0.0059 |
| GO:0019941 | modification-dependent protein catabolic process | 44 | PSMB10, CBX4, UBA7, RNF217, SENP5, MYCBP2, CYLD, ISG15, SQSTM1, MAP1LC3B, WWP2, RBCK1, RNF149, FBXL17, USP31, RNF144B, FBXL20, SOCS1, HERC6, UBR4, UBE2L6, MALT1, UBR2, PSMB8, PSMB9, WSB1, CBLB, TULP4, SENP1, PSMA6, PSME1, PSME2, FBXO17, PSMA4, DET1, KLHL12, MDM2, ZRANB1, FBXL7, AMFR, SIAH2, RNF19B, USP42, RNF41 | 1.52 | 0.0059 |
| **Annotation Cluster 12 Enrichment Score: 1.66** | | | | | |
| GO:0010033 | response to organic substance | 58 | ADCY3, A2M, THRA, PTGS2, OSMR, DICER1, TLR3, TLR4, PMAIP1, B2M, EDNRA, EIF4EBP1, GOT1, NDUFS4, CASP8, FOSL1, KCNMA1, EGFR, CIITA, IRS2, SP100, SOCS1, AARS, LIFR, DDIT3, EP300, MGEA5, ERN1, RIPK2, AMFR, NFE2L2, EIF2AK2, PPP1R15A, NGF, CYP1B1, ADH5, CALCOCO2, ASNS, TRIM16, TIMP4, C1S, GCH1, PLIN2, TAP2, BCL2, IDH1, TXNIP, HERPUD1, MAT2A, CFB, MAP1B, MALT1, IDO1, STAT1, ATF6, ADCY9, JAK2, VLDLR | 1.60 | 4.52E-04 |
| GO:0042493 | response to drug | 19 | TXNIP, GABRE, PTPRM, PTGS2, MAT2A, SNX27, MAP1B, SOCS1, TIMP4, STAT1, GCLM, CAPN3, DDIT3, EP300, PLIN2, BCL2, ABCC1, FOSL1, NGF | 1.74 | 0.0249 |

| **Table S16 Functional annotation clustering- Gene Ontology Term for Molecular Functions: MSC-17 vs. MSC-γ (upregulated genes)** | | | | | |
| --- | --- | --- | --- | --- | --- |
| **GOTERM ID** | **GO TERM** | **Count** | **Genes** | **Fold Enrichment** | **P-Value** |
| **Annotation Cluster 1 Enrichment Score: 5.19** | | | | | |
| GO:0005201 | extracellular matrix structural constituent | 11 | COL14A1, COL4A1, ELN, COL3A1, COL1A2, COL12A1, COL1A1, COL5A2, COL11A1, COL5A1, EMILIN1 | 10.19 | 1.09E-07 |
| GO:0048407 | platelet-derived growth factor binding | 5 | COL4A1, COL3A1, COL1A2, COL1A1, COL5A1 | 36.20 | 7.20E-06 |
| GO:0019838 | growth factor binding | 8 | WISP1, COL4A1, IGFBPL1, IGFBP6, COL3A1, COL1A2, COL1A1, COL5A1 | 6.07 | 3.38E-04 |
| **Annotation Cluster 2 Enrichment Score: 2.26** | | | | | |
| GO:0019842 | vitamin binding | 8 | LEPRE1, LEPREL2, PLOD2, P4HA3, CRABP2, FASN, GCAT, PC | 4.90 | 0.0012 |
| GO:0031418 | L-ascorbic acid binding | 4 | LEPRE1, LEPREL2, PLOD2, P4HA3 | 15.17 | 0.0022 |
| GO:0031406 | carboxylic acid binding | 8 | LEPRE1, LEPREL2, PLOD2, P4HA3, CRABP2, FABP3, FASN, PC | 4.43 | 0.0022 |
| GO:0019798 | procollagen-proline dioxygenase activity | 3 | LEPRE1, LEPREL2, P4HA3 | 34.14 | 0.0031 |
| GO:0031543 | peptidyl-proline dioxygenase activity | 3 | LEPRE1, LEPREL2, P4HA3 | 29.87 | 0.0041 |
| GO:0016706 | oxidoreductase activity, acting on paired donors, with incorporation or reduction of molecular oxygen, 2-oxoglutarate as one donor, and incorporation of one atom each of oxygen into both donors | 4 | LEPRE1, LEPREL2, PLOD2, P4HA3 | 11.38 | 0.0050 |
| GO:0005506 | iron ion binding | 11 | STEAP4, LEPRE1, LEPREL2, CYP2B6, PLOD2, CH25H, FADS1, RRM2, SCD, P4HA3, FADS2 | 2.84 | 0.0053 |
| GO:0016702 | oxidoreductase activity, acting on single donors with incorporation of molecular oxygen, incorporation of two atoms of oxygen | 4 | LEPRE1, LEPREL2, PLOD2, P4HA3 | 4.83 | 0.0493 |

| **Table S17 Functional annotation clustering- Gene Ontology Terms for Cellular Components: MSC-17 vs. MSC-γ (upregulated genes)** | | | | | |
| --- | --- | --- | --- | --- | --- |
| **GO TERM ID** | **GO TERM** | **Count** | **Genes** | **Fold Enrichment** | **P-Value** |
| **Annotation Cluster 1 Enrichment Score: 9.17** | | | | | |
| GO:0005578 | proteinaceous extracellular matrix | 25 | COL4A1, CD248, ELN, COL3A1, SPARC, CALR, MMP13, NTN1, COL5A2, COL5A1, MMP1, EMILIN1, LEPRE1, COL14A1, ANG, COL1A2, COL12A1, VCAN, MFAP2, ADAMTS12, COL1A1, MFAP4, COL11A1, ADAMTS4, COL10A1 | 5.87 | 5.02E-12 |
| GO:0031012 | extracellular matrix | 25 | COL4A1, CD248, ELN, COL3A1, SPARC, CALR, MMP13, NTN1, COL5A2, COL5A1, MMP1, EMILIN1, LEPRE1, COL14A1, ANG, COL1A2, COL12A1, VCAN, MFAP2, ADAMTS12, COL1A1, MFAP4, COL11A1, ADAMTS4, COL10A1 | 5.45 | 2.46E-11 |
| GO:0044420 | extracellular matrix part | 15 | COL4A1, COL3A1, SPARC, COL5A2, NTN1, COL5A1, COL14A1, ANG, COL1A2, COL12A1, MFAP2, COL1A1, MFAP4, COL11A1, COL10A1 | 9.64 | 4.10E-10 |
| GO:0005581 | collagen | 10 | COL14A1, COL4A1, COL3A1, COL1A2, COL12A1, COL1A1, COL5A2, COL11A1, COL5A1, COL10A1 | 21.48 | 5.24E-10 |
| GO:0044421 | extracellular region part | 39 | ENPP1, C3, CD248, IGFBP6, COL3A1, ELN, CALR, CXCL12, CCL7, MMP1, LIF, ANG, SAA1, C1QTNF1, COL12A1, ADAMTS12, LBP, COL11A1, COL10A1, PLAT, COL4A1, FIBCD1, SPARC, MMP13, COL5A2, NTN1, COL5A1, EMILIN1, SLIT3, LEPRE1, COL14A1, SFRP4, COL1A2, GDF11, MFAP2, VCAN, COL1A1, MFAP4, ADAMTS4 | 3.05 | 6.27E-10 |
| GO:0005576 | extracellular region | 57 | IGHG1, IGFBP6, IGHM, CXCL12, MMP1, WISP1, ANG, IGLV2-23, SAA1, COL12A1, REG3G, LBP, COL11A1, COL10A1, FIBCD1, MMP13, PLAUR, SLIT3, COL1A2, VCAN, MFAP2, EFNA4, COL1A1, VMO1, MFAP4, ADAM12, ADAMTS4, ENPP1, IGFBPL1, C3, CD248, COL3A1, ELN, CALR, CCL7, LIF, FNDC1, C1QTNF1, ADAMTS12, IGKC, THBS2, PLAT, COL4A1, RNASE4, SPARC, COL5A2, NTN1, COL5A1, CLEC11A, EMILIN1, C19ORF10, COL14A1, LEPRE1, SRPX2, SFRP4, GDF11, IGKV3-20, PLAU | 2.13 | 1.34E-08 |
| GO:0005583 | fibrillar collagen | 6 | COL3A1, COL1A2, COL1A1, COL5A2, COL11A1, COL5A1 | 37.59 | 2.80E-07 |
| **Annotation Cluster 2 Enrichment Score: 5.31** | | | | | |
| GO:0000786 | nucleosome | 14 | HIST1H2AB, HIST4H4, HIST1H4L, HIST1H4K, HIST1H2AG, HIST1H2AD, HIST1H2AE, HIST2H4A, HIST2H4B, HIST1H2BO, HIST2H2AB, HIST1H2BM, HIST1H2BN, HIST1H4A, HIST1H4B, HIST1H2BI, HIST1H4E, HIST1H4F, HIST1H4C, HIST1H4D, HIST1H4I, HIST1H4J, HIST1H4H, HIST2H3A, HIST1H2BB, HIST1H3J, HIST1H2BC, HIST1H1E, HIST1H2BE, HIST1H1C, HIST1H2BF, HIST1H1B, HIST1H2BG, HIST2H3C, HIST2H3D, HIST1H3A, HIST1H2AI, HIST1H3B, HIST1H2AH, HIST1H3C, HIST1H2AK, HIST1H3D, HIST1H3E, HIST1H2AJ, HIST1H2AM, HIST1H3F, HIST1H2AL, HIST1H3G, HIST1H3H, HIST1H3I | 16.71 | 1.40E-12 |
| GO:0032993 | protein-DNA complex | 14 | HIST1H2AB, HIST4H4, HIST1H4L, HIST1H4K, HIST1H2AG, HIST1H2AD, HIST1H2AE, HIST2H4A, HIST2H4B, HIST1H2BO, HIST2H2AB, HIST1H2BM, HIST1H2BN, HIST1H4A, HIST1H4B, HIST1H2BI, HIST1H4E, HIST1H4F, HIST1H4C, HIST1H4D, HIST1H4I, HIST1H4J, HIST1H4H, HIST2H3A, HIST1H2BB, HIST1H3J, HIST1H2BC, HIST1H1E, HIST1H2BE, HIST1H1C, HIST1H2BF, HIST1H1B, HIST1H2BG, HIST2H3C, HIST2H3D, HIST1H3A, HIST1H2AI, HIST1H3B, HIST1H2AH, HIST1H3C, HIST1H2AK, HIST1H3D, HIST1H3E, HIST1H2AJ, HIST1H2AM, HIST1H3F, HIST1H2AL, HIST1H3G, HIST1H3H, HIST1H3I | 12.24 | 8.95E-11 |
| GO:0000785 | chromatin | 14 | HIST1H2AB, HIST4H4, HIST1H4L, HIST1H4K, HIST1H2AG, HIST1H2AD, HIST1H2AE, HIST2H4A, HIST2H4B, HIST1H2BO, HIST2H2AB, HIST1H2BM, HIST1H2BN, HIST1H4A, HIST1H4B, HIST1H2BI, HIST1H4E, HIST1H4F, HIST1H4C, HIST1H4D, HIST1H4I, HIST1H4J, HIST1H4H, HIST2H3A, HIST1H2BB, HIST1H3J, HIST1H2BC, HIST1H1E, HIST1H2BE, HIST1H1C, HIST1H2BF, HIST1H1B, HIST1H2BG, HIST2H3C, HIST2H3D, HIST1H3A, HIST1H2AI, HIST1H3B, HIST1H2AH, HIST1H3C, HIST1H2AK, HIST1H3D, HIST1H3E, HIST1H2AJ, HIST1H2AM, HIST1H3F, HIST1H2AL, HIST1H3G, HIST1H3H, HIST1H3I | 5.26 | 2.48E-06 |
| GO:0044427 | chromosomal part | 18 | HIST4H4, AURKB, HIST2H4A, HIST2H4B, HIST1H2BO, HIST2H2AB, HIST1H2BM, HIST1H2BN, HIST1H2BI, HIST1H1E, HIST1H1C, HIST1H1B, HIST1H2AB, HIST1H4L, HIST1H4K, HIST1H2AG, HIST1H2AD, HIST1H2AE, SPC24, HIST1H4A, HIST1H4B, HIST1H4E, HIST1H4F, HIST1H4C, HIST1H4D, HIST1H4I, HIST1H4J, HIST1H4H, HIST2H3A, HIST1H3J, HIST1H2BB, HIST1H2BC, HIST1H2BE, HIST1H2BF, HIST1H2BG, BIRC5, HIST2H3C, HIST2H3D, HIST1H3A, HIST1H3B, HIST1H2AI, HIST1H2AH, HIST1H3C, SEC13, HIST1H2AK, HIST1H3D, HIST1H2AJ, HIST1H3E, HIST1H2AM, HIST1H3F, HIST1H2AL, HIST1H3G, HIST1H3H, HIST1H3I | 3.51 | 1.40E-05 |
| GO:0005694 | chromosome | 19 | CLSPN, HIST4H4, AURKB, HIST2H4A, HIST2H4B, HIST1H2BO, HIST2H2AB, HIST1H2BM, HIST1H2BN, HIST1H2BI, HIST1H1E, HIST1H1C, HIST1H1B, HIST1H2AB, HIST1H4L, HIST1H4K, HIST1H2AG, HIST1H2AD, HIST1H2AE, SPC24, HIST1H4A, HIST1H4B, HIST1H4E, HIST1H4F, HIST1H4C, HIST1H4D, HIST1H4I, HIST1H4J, HIST1H4H, HIST2H3A, HIST1H3J, HIST1H2BB, HIST1H2BC, HIST1H2BE, HIST1H2BF, HIST1H2BG, BIRC5, HIST2H3C, HIST2H3D, HIST1H3A, HIST1H2AI, HIST1H3B, HIST1H2AH, HIST1H3C, SEC13, HIST1H2AK, HIST1H3D, HIST1H2AJ, HIST1H3E, HIST1H2AM, HIST1H3F, HIST1H2AL, HIST1H3G, HIST1H3H, HIST1H3I | 3.11 | 3.77E-05 |

| **Table S18 Functional annotation clustering- Gene Ontology Terms for Cellular Components: MSC-17 vs. MSC-γ (downregulated genes)** | | | | | |
| --- | --- | --- | --- | --- | --- |
| **GOTERM ID** | **GO TERM** | **Count** | **Genes** | **Fold Enrichment** | **P-Value** |
| **Annotation Cluster 1 Enrichment Score: 9.54** | | | | | |
| GO:0071556 | integral component of lumenal side of endoplasmic reticulum membrane | 19 | HLA-DQB1, HLA-DQB2, HLA-DRB1, HLA-DRB3, HLA-A, HLA-C, HLA-B, HLA-E, HLA-DQA2, CD74, HLA-DQA1, HLA-G, TAPBP, HLA-F, PKD2, HLA-DRB4, HLA-DPA1, HLA-DPB1, HLA-DRA | 13.72 | 2.81E-17 |
| GO:0042613 | MHC class II protein complex | 16 | HLA-DQB1, HLA-DQB2, HLA-DRB1, HLA-DRB3, HLA-A, HLA-C, HLA-DMB, HLA-DMA, HLA-DQA2, CD74, HLA-DQA1, HLA-DRB4, HLA-DPA1, HLA-DPB1, HLA-DOA, HLA-DRA | 15.23 | 1.63E-15 |
| GO:0012507 | ER to Golgi transport vesicle membrane | 20 | HLA-DQB1, HLA-DQB2, HLA-DRB1, HLA-DRB3, HLA-A, VTI1B, HLA-C, HLA-B, HLA-E, HLA-DQA2, CD74, HLA-DQA1, HLA-G, HLA-F, B2M, HLA-DRB4, SEC22B, HLA-DPA1, HLA-DPB1, HLA-DRA | 8.06 | 1.10E-12 |
| GO:0042612 | MHC class I protein complex | 10 | HLA-DQB1, HLA-DRB1, HLA-A, HLA-C, MR1, HLA-B, HLA-E, HLA-G, HLA-F, B2M | 16.11 | 7.37E-10 |
| GO:0032588 | Gtrans-Golgi network membrane | 19 | HLA-DQB1, STX6, HLA-DQB2, RAB9A, HLA-DRB1, MYO1B, HLA-DRB3, HLA-DQA2, CD74, HLA-DQA1, KIF13A, COG5, RAB31, IGF2R, HLA-DRB4, HLA-DPA1, HLA-DPB1, HLA-DRA, RHOBTB3 | 4.79 | 5.45E-08 |
| GO:0030658 | transport vesicle membrane | 13 | HLA-DQB1, HLA-DQB2, HLA-DRB1, HLA-DRB3, HLA-DQA2, HLA-DQA1, CD74, HLA-DRB4, SNTB2, HLA-DPA1, HLA-DPB1, CEACAM1, HLA-DRA | 7.17 | 1.10E-07 |
| GO:0030666 | endocytic vesicle membrane | 14 | HLA-DQB1, HLA-DQB2, HLA-DRB1, HLA-DRB3, HLA-DQA2, CD74, HLA-DQA1, CAMK2D, HLA-DRB4, MDM2, HLA-DPA1, WASL, HLA-DPB1, HLA-DRA | 4.44 | 1.18E-05 |
| GO:0030669 | clathrin-coated endocytic vesicle membrane | 11 | HLA-DQB1, HLA-DQB2, HLA-DRB1, HLA-DRB3, HLA-DRB4, HLA-DPA1, HLA-DPB1, HLA-DQA2, CD74, HLA-DQA1, HLA-DRA | 5.62 | 1.68E-05 |
| **Annotation Cluster 2 Enrichment Score: 1.57** | | | | | |
| GO:0005839 | proteasome core complex | 5 | PSMB10, PSMA6, PSMA4, PSMB8, PSMB9 | 5.24 | 0.0135 |
| GO:1990111 | spermatoproteasome complex | 3 | PSMB10, PSMB8, PSMB9 | 12.57 | 0.0206 |
